# Confirmatory Factor Analysis and Propensity to Cheat Scale Validation in the Ethiopian Public Higher Education Institutions

**DOI:** 10.12688/f1000research.150357.1

**Published:** 2024-08-23

**Authors:** Dame Taye, Tesfaye Semela, Samuel Assefa

**Affiliations:** 1 School of Teacher Education, College of Education, Hawassa University, Hawassa, Hawassa, 8090, Ethiopia; 2 School of Teacher education, College of Education and Institute of Policy & Development Research (IPDR), Hawassa University, Awassa, Southern Nations, Nationalities, and People's Region, 8090, Ethiopia; 3 School of Teacher Education, College of Education, Hawassa University, Awassa, Southern Nations, Nationalities, and People's Region, 8090, Ethiopia

**Keywords:** exam cheating; propensity to cheats; university students; confirmatory factor analysis; construct validity

## Abstract

**Background:**

This study was primarily intended to develop and validate a comprehensive and psychometrically acceptable measure of students’ propensity to cheat (PTC) behavior among undergraduate students in Ethiopian universities by assessing their engagement in different types of cheating behavior.

**Methods:**

The present study employed an explanatory research design using a questionnaire based on the Propensity to Cheat Scale (PCS). The questionnaire was administered to 500 university students (male = 367 [73.4%]; female = 133 [26.6%]) selected from three Ethiopian public universities between November and January 2022. In order to measure the underlying variables of propensity towards cheating, a factor model is developed using exploratory factor analysis, and confirmatory factor analysis was employed to validate the students’ perceived PTC. The internal consistency of the PTC scale was assessed using reliability analysis, and validity evaluations were conducted to confirm the scale’s discriminant and convergent validity.

**Results:**

Confirmatory factor analysis (CFA) results revealed a good fit to the data, and the internal consistency of the PCS was found to be strong, providing a reliable measure of students’ propensity for cheating. Validity evaluations, including discriminant validity and convergent validity, confirmed the validity of the scale. The average variance extracted (AVE) and composite reliability values also supported the scale’s convergent validity. The multidimensional concept of the PTC was supported by a four-factor solution consisting of 26 reliable and valid items.

**Conclusion:**

The findings of the study demonstrate that the scale has also provided sufficient evidence of convergent and discriminant validity. By establishing discriminant and convergent validity, as well as reliability, through different validation procedures, the study has provided strong evidence for the effectiveness of the PCS as an instrument for determining whether university students are likely to engage in cheating behavior.

## 1. Introduction

### 1.1 Background of the study

Academic dishonesty is a broad term that encapsulates various detrimental behaviors within educational environments, such as plagiarism and falsification of information. Although difficult to define precisely ( Lambert et al., 2003), academic dishonesty involves a variety of damaging activities, such as plagiarism or making false excuses ( Yazici et al., 2011). The concept of academic cheating was initially described by Rich (1984) as deceitful actions related to coursework, including cheating on tests, exams, and assignments, along with activities like obtaining exams illegally, plagiarizing content, manipulating data, and tampering with library resources.

According to Davis et al. (2009), academic cheating involves deceiving or defrauding another’s through misleading or dishonest actions. They emphasize that it entails students engaging in behaviors that trick instructors into believing that the academic work submitted is their original creation. Academic cheating can be defined as “intentionally unethical behaviour” ( Von Dran et al., 2001, pp. 40) and “using deception (fraud) in academic work” ( Cochran, 2016, pp. 814), both of which result in a breach of the defined rules and accepted standards, granting cheaters an unfair benefit over those who do not cheat ( Dick et al., 2003). ensuring quality outcomes. In order to guarantee quality outcomes, student assessment should actually be seen as a complex and multifaceted activity that requires alignment, balance, and rigor ( Joughin & Macdonald, 2004). Academic integrity is paramount to upholding the quality of education, and reducing academic fraud is essential for a thorough evaluation process.

Academic dishonesty by students, which this study defines as plagiarism and cheating on assignments and tests ( Adesile et al., 2016; Ives et al., 2017; Ives and Nehrkorn, 2019; Jensen et al., 2002; Lin and Wen, 2007; Owunwanne et al., 2010), undermines the goals of higher education institutions. It compromises assessment validity since results cannot be generalized to represent trustworthy estimates of students’ grasp of course-work content ( Bouville, 2010; Muñoz-García and Aviles-Herrera, 2014).

The topic of propensity to cheat (PTC) among students is a significant concern in higher education institutions. A student’s propensity is the likelihood that they will engage in a specific action or behavior. Propensity is defined in the literature as the tendency or predisposition to participate in a particular behavior, in this case, cheating. Cheating tendencies are the inclinations of an individual or a group of individuals to commit crimes prior to, during, or following an exam with the intention of unfairly benefiting from an unfair advantage over other students ( Offor, 2009). Cheating propensity is defined as a tendency, disposition, or likelihood that a student may commit examination malpractice in light of these definitions. For the objectives of this study, understanding this broader concept of cheating propensity is essential for developing effective strategies to prevent academic misconduct.

### 1.2 Prevalence of academic dishonesty

Various studies indicate that academic dishonesty is more prevalent than ever, and actions need to be taken by universities to educate students as well as faculty members about academic integrity and ethical professionalism ( Diego, 2017; Feday, 2017; Jepngetich et al., 2017; Mebratu, 2014; Nelson et al., 2012). According to studies, dishonest behavior that began in high school can continue in college, and similarly, college students who engage in academic dishonest behavior are more likely to engage in dishonest behavior outside of the classroom ( Harding et al., 2004). This suggests that there is a chance that dishonest behavior will continue over time and in different circumstances.

Academic cheating is a widespread problem around the world, with high rates of cheating reported among students in many countries. In the United States, surveys have revealed that up to 86 percent of college students have engaged in dishonesty in the classroom ( McCabe et al. 2006). Bernardi et al. (2008) conducted a study to compare the perspectives of students from industrialized countries on cheating behavior. They discovered that 51% of the people in the samples admitted to cheating. According to Yussof and Ismail (2018), high rates of Malaysian students have cheated, primarily in assignments and quizzes that carry a lower weight in the final grade and are subject to less supervision and punishment. In South Korea, Ahmed (2018) discovered that 65 percent of pupils misbehaved using electronic media, and 80 percent of students committed academic fraud.

In Africa, academic dishonesty is equally prevalent. According to Umar (2004), cheating on exams was almost a standard practice in Nigeria. According to Munachonga (2015), examination misconduct has become increasingly common in Ghana. This is mostly because candidates are afraid of failing, lack confidence, are lazy, don’t prepare well, and, most importantly, are unable to apply themselves to their studies. Munachonga (2015) noted that officials of the Test Council of Zambia, the nation’s supervisory organization for exams, have occasionally been involved in examination malpractices.

When compared to various other studies carried out worldwide, the prevalence rate found in Ethiopia is high. Even though there is little to no evidence on the subject, cheating has from time to time increased in Ethiopia ( Tefera & Kinde, 2009). Chala (2021) discovered that, with an incidence a rate ranging from 53 to 96 percent, academic dishonesty is a severe problem impacting university students in Ethiopia. Research conducted locally has, in one form or another, verified the growing epidemic of academic cheating in Ethiopian education at all levels ( Feday, 2017; Hailu, 2015; Mebratu, 2014; Tefera & Kinde, 2010; Wondifraw, 2021). Furthermore, according to the Ethiopian Education Development Roadmap (2018–30), students frequently cheat on tests, and teachers are afraid that aggressive student evaluation may lead to an inaccurate assessment of their work and inflated grades ( MoE, 2018).

Academic dishonesty can be justified in various ways in an academic setting. The majority of the time, in Ethiopia, the behaviors take the form of plagiarism and exam cheating (papers and assignments). To mention a few results of research done in the Ethiopian setting, Feday (2017) and Mebratu (2016) found that plagiarism in written work and exam cheating are the most common forms of academic dishonesty. Furthermore, a number of lecturers have expressed concern about the increased incidence of plagiarism, assignment, and exam cheating, which suggests that Ethiopia’s public universities agree with the researchers’ concerns based on their observations and teaching experience. In particular, some Hawassa and Wolkite Universities College of Science and Engineering students engage in academic dishonesty, according to the researchers’ experience from several years of teaching students in a course. Additionally, some students are opposing the growing practice of exam cheating and plagiarism in coursework. Based on our observations, a major contributing factor to exam cheating is a student’s lack of academic proficiency in a particular area. In this sense, the chance of earning a high cumulative grade point average is increased by cheating. Closing the gap and validating a scale that might possess the required psychometric properties to specify measurement accuracy was, thus, the primary objective of this work.

### 1.3 Problem statements

Numerous measures have been developed to measure university students’ tendencies toward academic dishonesty, according to a review of previous studies on the subject. Academic cheating is a multidimensional issue that affects people all around the world ( Imran & Nordin, 2013; Iberahim et al., 2013; Nazir & Aslam, 2010; Thomas, 2017; Tadesse & Getachew, 2010; Saidin & Isa, 2013; Yang et al., 2013). The academic practices survey is a two-dimensional construct with plagiarism (items related to written work) and cheating (items associated with class tests and exams), as discovered by Roig and DeTommaso (1995) and Ferrari (2005). Additionally, Adesile et al. (2016) developed a three-dimensional scale for measuring academic integrity that takes into account plagiarism, cheating, and research misconduct. Finally, in light of the above-mentioned differences in the dimensions of academic dishonesty, the dimensions of PTC that are the subject of this study are cheating on tests, assignments, research work (plagiarism), and mutilation/theft of library resources.

The main issue with this is the legitimacy of how the PTC is measured in a setting where academic dishonesty is common. Few studies have thus far particularly addressed the PTC, but none of these studies are known to have used a well-developed and validated questionnaire. More specifically, except for a brief mention of the reliability coefficient, not much is known about confirmatory component analysis ( Adesile et al., 2016). However, as Imran and Nordin (2013) pointed out, the dimensionalities of most of the measures were not carefully examined, and there was little indication that they have meaningful psychometric properties. Moreover, discriminant validity refers to a scale’s ability to discern between the relevant concept and other related constructs. Because previous scales were unable to show this differentiation, there is confusion regarding interpretation ( Bashir & Bala, 2018). By conducting validity and reliability tests on older scales, researchers can assess these difficulties and recommend additional research.

Therefore, a valid and culturally reliable propensity to cheat scale (PCS) is needed. It is therefore necessary to develop, assess its dimensionality, and validate an instrument measuring the PTC constructs. In order to guarantee that psychological and behavioral constructsare suitably defined and evaluated, it is imperative that instruments undergo validation and evaluation ( Hair et al., 2010). Hair et al. also recommended that researchers continuously confirm the validity and unidimensionality of their conceptions, even when employing instruments that are well accepted. Regarding this, the measure’s discriminant and convergent validity were allegedly determined using a Multimethods Multitraits (MMMT) investigation, but there was no solid evidence to support this claim.

The main reason for the emergence of this study is the absence of an instrument to measure the tendency for academic dishonesty in Ethiopian higher education institutions. Despite the researchers’ experience in the study field, no prior research has been done on the psychometric qualities and validity of the PCS employed by these institutions. Therefore, the researchers sought to address this gap in the literature by conducting a study to evaluate the psychometric properties of the PCS in the context of Ethiopian higher education institutions.

To achieve this, a confirmatory factor analysis (CFA) was conducted to determine the scale’s applicability and assess the convergent and discriminant validity of students’ propensity for academic fraud. This study utilized a sample of public university students in Ethiopia, where no previous research of this kind had been conducted. Therefore, this research sought to contribute to existing knowledge by examining the effectiveness of the PCS in measuring academic dishonesty tendencies among Ethiopian higher education students and addressing the research gap in this area.

### 1.4 Research questions

In order to assess the convergent and discriminant validity of the scale, this study attempted to ascertain the construct validity of a locally developed PCS. The objective of this research was to assess the measurement model validity of the PCS among science and engineering students in order to add to the body of data supporting its construct validity. Analyze the PCS’s convergent and discriminant validity in particular.

The major focus of this study was on the latent construction of students’ propensity for academic misconduct in the higher education setting in Ethiopia, which was represented in four dimensions: (1) corresponding to cheating on tests and examinations, (2) cheating on assignments, (3) cheating on research work (plagiarism), and (4) theft and mutilation of library materials. The researchers concluded that it would be appropriate to validate the locally developed PCS in order to assess its convergent and discriminant validity. Two distinct research questions were formulated in order to achieve the research objectives:

1. To what extent does the PCS demonstrate convergent validity?
2. To what extent do the items in the instrument of PCS show discriminant validity?

## 2. Methods

In Ethiopia’s public universities, this study was conducted among students studying Science and Engineering. The researchers carried out the factorial structure, exploratory factor analysis, and measuring tool reliability in the initial stage. In the current study, the researchers looked at the degree to which CFA on the same set of observations can support the findings from the EFA study.

### 2.1 Research design

According to Clark and Creswell (2008, p. 58), a research design is the “procedures for collecting, analysing,interpreting, and reporting data inresearch studies.” It is the overarching strategy for linking theoretical research issues with relevant (and doable) empirical research. In other words, the study design determines how the necessary data will be collected, how it will be analysed, and how it will be used to address the research question ( Grey, 2014). There are three different types of study designs that can be used: exploratory, descriptive, and explanatory ( Robson, 1993). His classification system is based on the goals of the research field because each design has a distinct ultimate goal. By using an explanatory research design in this study, we aim to understand the relationship between students’ reported PTC and their academic self-concept and academic accomplishment through a quantitative approach. A correlational research methodology used for explanatory purposes allows the researchers to assess the degree of association between two or more variables. Additionally, using this style of research design enables the researcher to gather data all at once ( Creswell, 2015).

### 2.2 Population of the study

The study focused on fourth-year science and engineering regular undergraduate students attending Ethiopia’s public universities. The researchers selected this specific group from the target population of first- to fifth-year science and engineering undergraduate students in the participating universities. The target population consisted of 6,524 students, including 4,901 males and 1,623 females.

### 2.3 Samples and sampling procedure

In this study, the sample selection procedure was conducted in a systematic manner. First, three public universities, namely Hawassa University, Ambo University, and Wolkite University, were selected using the purposive sampling technique. These universities were chosen to represent both old and new public universities. Next, a purposive sample strategy was employed to select the colleges within the participating universities. Specifically, the colleges of Natural and Computational Science and Engineering/Institute of Technology were chosen. Following the selection of the colleges, departments were randomly chosen from among the selected colleges. This random selection ensured a diverse representation of departments within the chosen colleges. Finally, a simple random selection procedure was used to select students from the chosen departments for the study. This ensured that the sample of students was representative of the larger student population within the universities.

The researcher converted Gomez’s (2013) Sloven’s Formula into a formula to compute the sample size for this study. The sample size was determined using the following formula. The following is how this formula is laid out:

$$N=\frac{N}{\left(1+\left(N\right)\left(e^{2}\right)\right)}$$

Where, *N* is the target population size

*n* is the sample size and

*e* is the level of precision (acceptable margin of error at 5% (95% confidence level), P = .5 are assumed for equation

For this study the target population ( *N*=4120) then the required sample size is calculated as

$$N=\frac{6524}{\left(1+6524\right)\;{\left(0.05\right)}^{2}}=377$$

When selecting a sample size for the study, several criteria need to be considered. These include the size of the population, the level of confidence, the margin of error (confidence interval), the number of variables used in the study, the statistical analysis technique to be employed, as well as time, money, and effort constraints. In this regard, Ellen (2014) asserts that a formula must be employed to account for the margin of error and confidence level when a sample is drawn from a population. Further, research claims that Sloven’s formula should be used when there is little information available about how a population will behave (such as in the case of this study polling college students to assess their opinions and likelihood to cheat online), other than its size ( Ellen, 2014; Kavai, 2014; Tanty & Rahayu, 2014; Abun & Cajindos, 2012). This equation enables the researcher to accurately sample the desired population ( Ellen, 2014). It is thought reasonable to select a sample size using Slovin’s formula, which was developed in 1960. This is true in particular when there is uncertainty regarding the behavior of the population ( Isip, 2014).

Of the 550 students invited to participate in the study, only 500 completed the questionnaire correctly. The universities, their respective colleges, and the study participants are summarized in Table 1.

**Table 1.** Universities, Colleges, and Number of Fourth Year Undergraduate Students Included in the Study.

Name of your University			Gender		
			Male	Female	Total
Ambo University	Name of College/Institute	College of natural and computational science	60	26	86
		Engineering	51	26	77
	Total		111	52	163
Wolkite university	Name of College/Institute	College of natural and computational science	35	16	51
		Engineering	61	14	75
	Total		96	30	126
Hawassa University	Name of College/Institute	College of natural and computational science	64	36	100
		Engineering	96	15	111
	Total		160	51	211
Total	Name of College/Institute	College of natural and computational science	159	78	237
		Engineering	208	55	263
	Total		367	133	500

### 2.4 Research instruments

This study employed a self-administered questionnaire for the measurement of PTC by four first-order factors: mutilation of library materials, cheating on tests or exams, cheating on assignments, and cheating on research work. All of the dimensions and their subdomains have been constructed, and this study included the reliability and validity (convergent and discriminant validity) of the scale. Notion validity in this context refers to how well a scale’s items are suited to measuring a certain theoretical construct ( DeVon *et al*., 2007; Kane, 2001). Discriminant and convergent validity are two fundamental features of construct validity, according to most arguments ( Cronbach & Meehl, 1955; Campbell & Fiske, 1959; Pike, 1992, 2006). The construct validity of the PCS was then investigated in this study by examining both its convergent and discriminant validity.

To assess academic self-concept, Liu and Wang (2005) developed a scale consisting of 20 items. These items are designed to measure students’ confidence and motivation in their academic abilities within a university environment. Participants rated each item on a five-point Likert scale, ranging from strongly disagree (1) to stronglyagree (5). The internal consistency reliability of this scale is 0.85.

**2.4.1 Validation of the measures**

Following the pilot study validation of face validity, content validity, and reliability, more testing of other types of validity and reliability is necessary before they can be fully implemented and taken into account during the PCS validation process in this study on an adequate number of participants. Through CFAs and EFAs, the construct validity validation procedures as well as the dimensional structure of the PCS were assessed in terms of exploration, item reduction, confirmation, and validation.

In a validation study, 550 students from three public universities were given a questionnaire to determine their PTC and their perception of their academic self-concept. Out of the initial 550 participants, 500 students fully answered the PTC and academic self-concept items and were included in the analysis. Among these 500 students, there were 367 males and 133 females, with ages ranging from 18 to 40 years (M = 1.18, SD = .49). Additionally, 50 students who partially answered the items were disqualified from the study.

Prior to data processing, the frequency distribution and the minimum and maximum scores for each item were used to verify that the database had been entered accurately. Additionally, each measure’s underlying assumptions for CFA were examined, and they were confirmed to be valid for the analysis.

### 2.5 Data collection procedures

In November 2022, data collection for this research began immediately following the enrolment of fourth-year students for the first semester of the academic year. The measures were translated into Amharic and back-translated by three translators from the English and psychology departments of Wolkite University to validate the instrument (PSC). Approval was obtained from each college dean before the commencement of data collection for this study. The college deans informed the department heads and instructors of the individual institutions about the study’s objectives. Subsequently, arrangements were made for the researchers and data collectors to recruit participants in the classroom. Teachers at the universities were tasked with informing and motivating students to participate in the study and scheduling times for the researchers to speak with them during regular class hours. Before delivering the questionnaires, the fourth-year students selected for the study provided their verbal informed consent.

The study began with a thorough briefing for the participants, which included the goal and objective of the research, the focus of the questionnaire, and the potential benefits of the study for society and individuals. The participants were then asked for their consent to participate voluntarily, with the assurance that they could withdraw at any time without consequences and that all information provided would be kept confidential. Following the briefing, the researchers distributed the Amharic revised version questionnaires to students at Hawassa University, Ambo University, and Wolkite University from November to January 2022 during regular class time.

### 2.6 Methods of data analysis

The data processing procedures began by analyzing incomplete questionnaire responses and discarding any that were not complete. The remaining questionnaires were then coded and entered into a computer for further analysis. The data was cleaned to remove any errors that may have occurred during the coding process. Additional checks were conducted to ensure the accuracy of the data entry and measurement scales. The data was then examined for outliers, normality, skewness, and kurtosis to determine if it was at a normal level.

CFA is another kind of measurement-related structural equation modeling (SEM) ( Brown, 2015). CFA should be used to substantiate the connections made between the items and the corresponding factors. This gives metrics to evaluate how well the suggested theoretical model fits the collected data and allows these relationships to be fixed in the measurement model ( Stevens, 2012). According to Brown (2015), CFA is therefore seen as a crucial instrument for validation in the social and behavioral sciences. Regarding this, structural equation modeling (SEM) with CFA was used to verify the measurement and structural model.

The Statistical Package for the Social Sciences (SPSS) and STATA 14 were used for the analysis of the data. Cronbach’s alpha was used to evaluate internal consistency and reliability. The convergent and discriminant validity of the PCS were also examined to evaluate construct validity. CFA was utilized to address research questions such as whether the sample’s data matched the established PTC measurement model.

In addition, research questions like how much the recently developed PCS demonstrates convergent validity were addressed using Composite Reliability (CR), Average Variance Extracted (AVE), Maximum Shared Squared Variance (MSV), and Average Shared Squared Variance (ASV). While the research question, such as how much the newly constructed PTC instrument demonstrates discriminant validity with other validated factors (the academic self-concept), was addressed using Pearson correlation analysis.

## 3. Analysis and results

Data from the SPSS software version 25 was input and validated for missing data before being transferred to STATA Version 14 for CFA analysis. CFA was used to further validate the factorial structure of the PTC questionnaire that was initially identified through exploratory factor analysis (EFA) with a sample size of n = 550.

To analyze whether or not the established PTC measurement model fits to the data, CFA was used, and to analyze both convergent validity and discriminant validity, composite reliability (CR), average variance extracted (AVE), maximum shared squared variance (MSV), and average shared squared variance (ASV) were used.

### 3.1 Confirmatory Factor Analysis (CFA) and overall construct validity

After the exploratory factor analysis conducted in Study 1, CFA is used to establish the data set’s factor structure. The exploration of factor structure (how variables link and are grouped based on inter-variable correlations) in the CFA confirms the factor structure that was extracted in the exploratory factor analysis (EFA) ( Brown, 2015). To evaluate model parameters and fit indices across the clustering of independent variables, a CFA was also carried out for all data sets to look into the general concept validity ( Chuang, Weng, & Huang, 2015).

**Figure 1.**
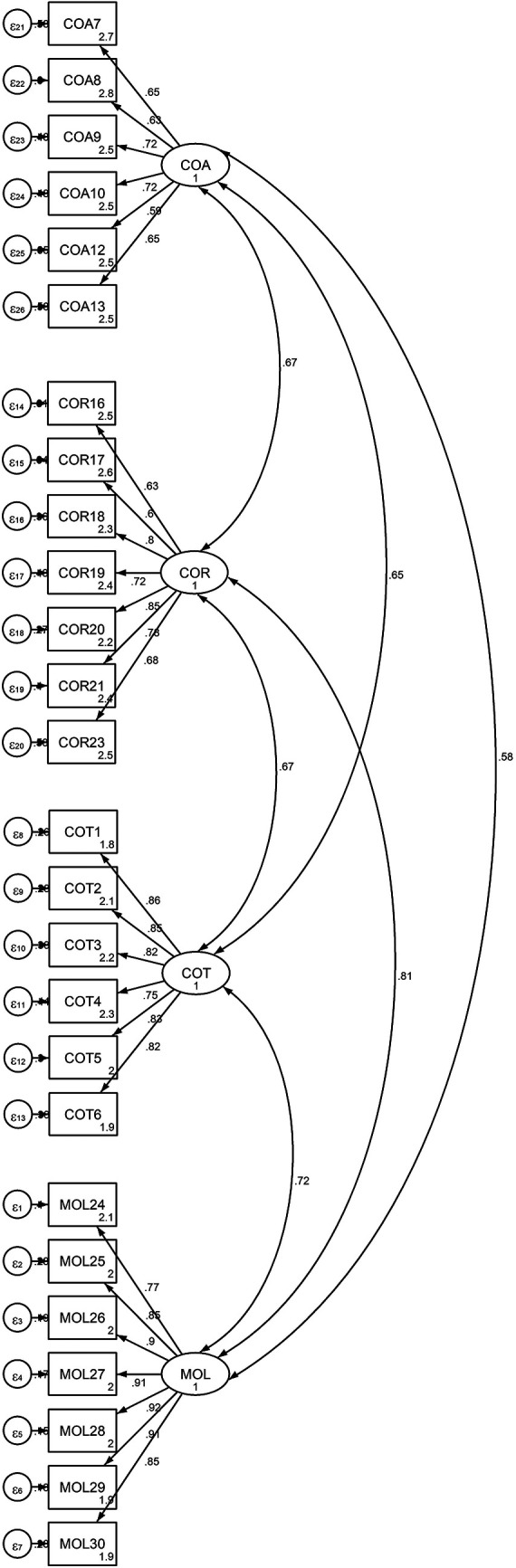
Output of the measurement model for Latent Variable “Four-Factor of PTC” and Observed Indicators.

Both the first-order (measurement model) and the second-order (structural model) were used to validate the model. The findings, as shown in Figure 1 and Table 1, strongly support the measurement of PTC using four first-order factors: cheating on tests or examinations, cheating on assignments, cheating on research work, and mutilation of library materials, with a total of 26 items based on the PSC. These items were designed to elicit responses from university students regarding how they perceived their academic cheating.

### 3.2 Testing the measurement model

The measurement model was put to the test, and then the structural model was examined using the standards outlined in the literature (see Hair et al., 2016). The measurement models assess the fit of the data to the model and the link between the latent variable and its indicators ( Henseler et al., 2009). The first-order CFA model has been developed to measure the relationships between the four dimensions (cheating on tests, cheating on assignments, cheating on research work, and theft and mutilation of library materials) and the twenty-six associated items that fit the empirical data well. The measurement models for the unobservable (latent) variables were generated and validated using the CFA approach in this study. Because the observed variables of the PTC subscales were determined in advance based on a review of the literature and interviews with experts, a CFA was used to assess the link between cheating on tests, cheating on assignments, cheating on research work, and theft and mutilation of library materials and each of their observed variables in order to develop a measurement model that fit the empirical data well. Figure 1 below shows the measurement models for the four-factor PTC. Figure 1 is available in the data repository ( Gizaw, 2024).

Multiple fit indices were employed to assess the appropriateness of the CFA model’s fit to the data, including the χ ^2^/df ratio, Tucker Lewis Index (TLI), comparative fit index (CFI), and root mean square error of approximation (RMSEA). The goodness-of-fit indices of the CFA model for the PCS are shown in Table 2. Despite the fact that the chi-square statistic has a significant value (χ ^2^/df = 3.877, p =.000), alternative fit indices are encouraging due to the highly sensitive nature of this statistic to large samples ( Bentler & Bonett, 1980; Stevens, 2002). The CFA results demonstrate that the model had fit statistics such as RMSEA = 0.076, RMR = 0.045, TLI = 0.907, and CFI = 0.916. According to the literature, the chi-square value of less than 5 is acceptable, and less than 3 is good ( Marsh & Hocevar, 1985). Hu and Bentler (1999) and Browne and Cudeck (1992) proposed (RMSEA less than .08, RMR less than .05, TLI and CFI greater than .90) as the recommended values for this fit statistic. Based on the indices obtained after CFA, the results indicated that each dimension’s factor model fit each dimension well. Finally, the coefficient of determination for the entire model is extremely high (CD = 1.000).

**Table 2.** Fit Indices Statistics Output for Measurement and Structural Model Analyses of PCS.

Goodness fit indices	Recommended value	Source(s)	Obtained value of First-order factor model	Obtained value of second-order factor model
Comparative Fit Index (CFI)	>0.90	Bentler, 1990	0.916	0.913
Tucker-Lewis Index (TLI)	>0.90	Tucker and Lewis, 1973 Hair et al., 2010	0.907	0.904
Root mean square error of approximation (RMSEA)	between 0.05 and 0.08		0.076	0.077
Standardized Root-Mean Square Residual (SRMR)	<0.05	Hu and Bentler (1999)	0.045	0.049
Coefficient of determination (CD)			1.000	0.912

### 3.3 Testing the structural model

It is required to move on to the structural model after developing the measurement model. A structural model explains how the many constructs in a model relate to one another. The four measurement models become structural models in the second-order CFA model by linking them collectively or through PTC, as shown below. In order to validate the four-factor structure developed by the exploratory factor analysis (EFA), a second-order CFA was applied. The next phase in structural equation modeling (SEM) analysis is to examine the goodness of fit of the full structural equation model and determine if an acceptable full structural model is obtained after acquiring an acceptable measurement model that fits the empirical data. A set of goodness-of-fit analyses was carried out for this purpose.

**Figure 2.**
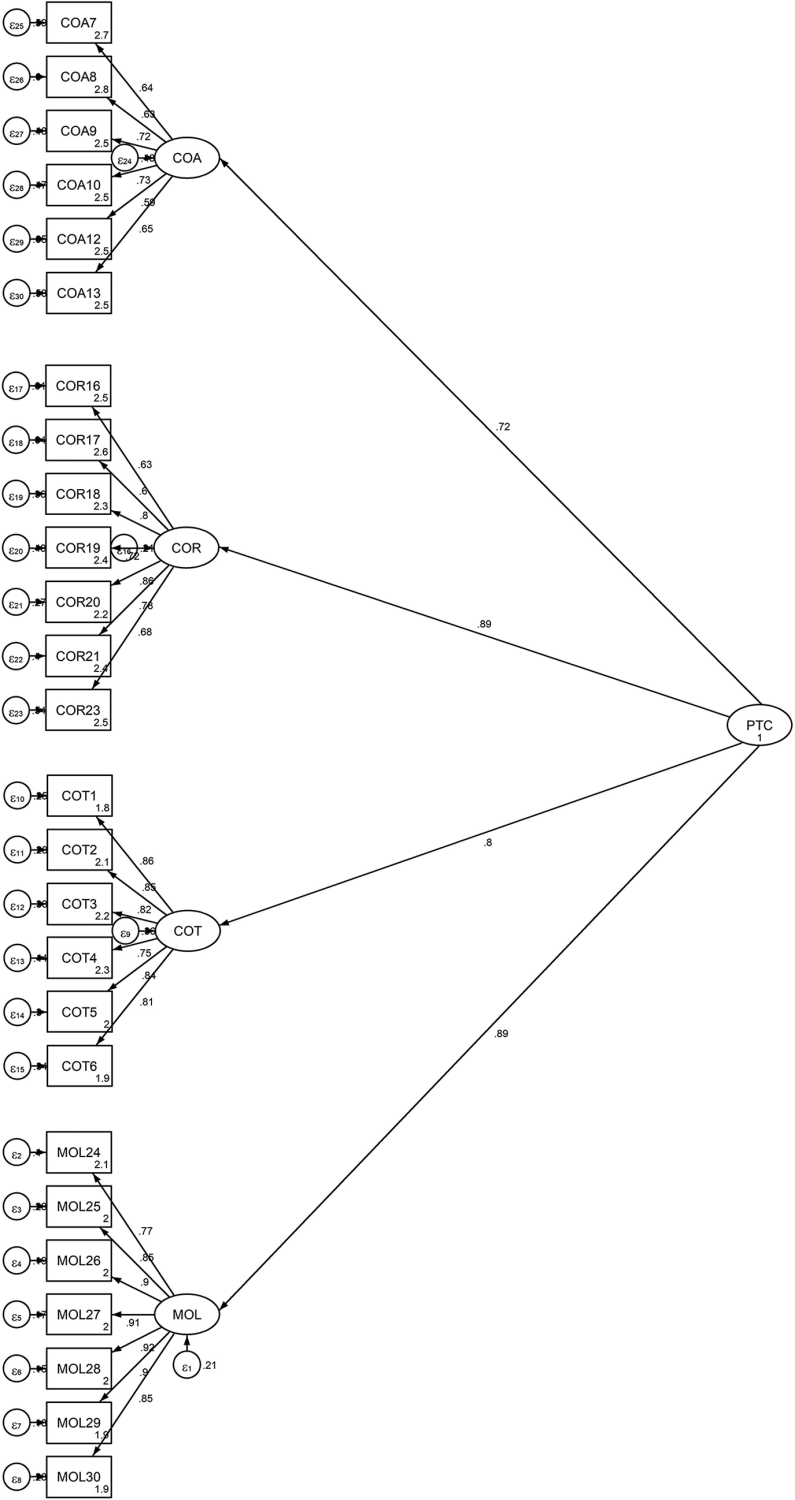
Output of the Structural Model for the Students’ Propensity to Cheat.

Figure 2 is shows the factor distributions and values obtained from the second-order CFA model. Figure 2 available in the data repository ( Gizaw, 2024).

To examine the relationships, a structural equation model created with STATA 14 was utilized. A good fitting model is acceptable if the CMIN/df value is less than 5; the Tucker Lewis Index (TLI) ( Tucker and Lewis, 1973); and the confirmatory fit index (CFI) ( Bentler, 1990) is greater than 0.90 ( Hair et al., 2010). In addition, an adequate fitting model was accepted if the standardized Root-Mean Square Residual (SRMR) estimated by STATA was less than 0.05 and the Root Mean Square Error of Approximation (RMSEA) was between 0.05 and 0.08 ( Hair et al., 2010). The alternative fit indices are encouraging due to the sensitivity of the chi-square statistic to sample size: χ ^2^/df = 3.966, CFI = 0.913, TLI = 0.904, RMSEA = 0.077 and RMR = 0.049. The chi-square statistic has a significant value, χ2 (295) = 1170.069, p. <000. In this regard, the second-order CFA model was suitable since all of the indices met the threshold values of the requirements and because their values were comparable to those of the first-order CFA model. Finally, the total model’s coefficient of determination (CD = 0.912) is very high.

The study used CFA to explore the underlying latent variable structure of the PCS from the same sample on which the EFA was administered in order to evaluate the goodness of fit of the four-factor structure produced from the EFA.

Table 3 and Figure 2 demonstrate that each factor loading was significant at p<.05. Additionally, the results of the standardized factor loading coefficients showed that the subscales measuring cheating on exams and tests ranged from .75 to .86, the subscales measuring cheating on assignments and research work from .59 to .72, and the subscale measuring theft and mutilation of library materials from .77 to .92.

**Table 3.** Confirmatory Factor Analysis Results for Propensity to Cheat (N = 500).

Latent variable	coding of items	Observed and unobserved variables	Standardized factor loadings	Standard errors	R ^2^ values
Cheating on tests	COT5	I rely on bribes with an instructor to get test information.	.86 *	.01	.74
	COT6	I copy answers from a classmate’s test paper during an exam while the instructor is not looking.	.85 *	.01	.72
	COT8	I use “signals” to ask my classmates for answers during a test.	.82 *	.02	.67
	COT9	I utilize “signal’ to share answers to classmates during an examination.	.75 *	.02	.56
	COT10	I cheat by writing the answers to questions on a soft or handkerchief and pretending to cough while using the soft.	.83 *	.02	.70
	COT11	I personally exchange test papers with someone during a test.	.82 *	.02	.67
Cheating on assignments	COA22	I would cheat on an assignment if I can get an opportunity	.65 *	.03	.42
	COA23	I am likely to cheat on an assignment in the future.	.63 *	.03	.40
	COA26	I submit the project/assignment/paper in my name after getting it prepared by my friends.	.72 *	.03	.52
	COA27	I am resubmitting an assignment from a previous subject in a new subject.	.72 *	.03	.52
	COA29	I provide false justifications to get an extension of a deadline for submitting an assignment.	.59 *	.03	.35
	COA30	I copy a homework assignment from a different portion of the class.	.65 *	.03	.42
Cheating on research work	COR45	I copy and modify a few phrases or sentences from a published work for inclusion in a written research paper without providing acknowledgment to the author.	.63 *	.03	.39
	COR46	I create or falsifying research data, using a secondary source as a primary source.	.60 *	.03	.36
	COR47	I fabricate or falsifying a bibliography.	.80 *	.02	.64
	COR48	I am working on a research paper for another student.	.72 *	.02	.52
	COR49	I pay for a research paper to be written for me.	.85 *	.02	.73
	COR50	I submit a research paper prepared by someone else as my own work, in part or in whole.	.78 *	.02	.60
	COR52	I present a study paper that I got from a "Web site, or online sources," as my own work.	.68 *	.03	.47
Theft and Mutilation of library Material	MOL55	I take out library books so that my classmates do not get required content.	.77 *	.02	.60
	MOL56	I take material from the library without first checking them out.	.85 *	.01	.72
	MOL57	I cut pages out of journals or books in the university library.	.90 *	.01	.81
	MOL58	I eliminate a reference from the library shelf to prevent other students from gaining access to the information.	.91 *	.01	.83
	MOL59	I hide library material in my pocket, handbag, and exercise book.	.92 *	.01	.85
	MOL60	Confusing/diverting the attention of people at the circulation desk.	.91 *	.01	.82
	MOL61	Smuggling it out of the library with the help of library workers.	.85 *	.01	.72
PTC	COT	Cheating on tests	.89 *	.02	.64
	COA	Cheating on assignments	.72 *	.03	.52
	COR	Cheating on research work	.89 *	.02	.79
	MOL	Theft and mutilation of library material	.89 *	.02	.79

* p < 0.05.

Furthermore, the squared multiple correlation coefficients also provide the coefficient of determination (R2), which illustrates the extent to which a factor may account for the variance in an item. In this regard, the COT5 item has the highest R2 (.74), meaning that the latent variable test-cheating accounts for 74% of the variation in the COT5 item.

The model was able to explain 64 percent, 52 percent, 79 percent, and 79 percent, respectively, of the factors related to cheating on tests, cheating on assignments, cheating on research work, and theft and mutilation of library materials ( Table 3 below). The results showed that, after exam cheating, the two most important factors were theft of library items and exam cheating. Cheating on exams, assignments, research work, and mutilating library materials collectively account for 91% of the variance in the PTC, as indicated by the squared multiple correlation for the PTC of 0.91 (see Figure 2).

## 4. Validity assessment

Analyzing both convergent validity and discriminant validity allows for the examination of construct validity. The construct validity of a scale is examined after its dimensionality and reliability have been confirmed to be appropriate. The CFA also considered the validity of both convergent and divergent findings.

### 4.1 Convergent validity

In this study, the PTC measure was evaluated for convergent validity by examining the internal consistency of the indicators measuring the same construct. In Bagozzi’s (1981) definition of convergent validity, the emphasis is on the internal consistency of indicators measuring the same construct. Researchers utilized reliability measures as one of the criteria to assess convergent validity ( Fornell & Larcker, 1981). However, they recognized that evaluating convergent validity just on the basis of reliability is insufficient. To further assess convergent validity, a measurement model was estimated in which all indicators were related to the constructs they were intended to measure and not directly related to constructs they were not intended to measure ( Fornell & Larcker, 1981; Hair et al., 2009). All indicators converge well on their own construct when the predicted measurement model sufficiently matches the data. Since model fit does not ensure measurement quality, researchers have argued that an appropriate model fit is insufficient to support convergent validity ( Fornell & Larcker, 1981). As a result, additional criteria have been proposed to ensure that the indicators truly measure the intended construct.

The standardized factor loading, extracted average variance (AVE), and composite reliability (CR) tests were also used to assess convergent validity. Numerous studies have proposed evaluating convergent validity by looking at the statistical significance of standardized factor loadings (e.g., Dunn et al., 1994). For instance, Hair et al. (2009) claimed that all standardized factor loadings should be at least 0.5 and, ideally, at least 0.7, while Stevens (2002) suggested that the value of a factor loading should be more than 0.4 for interpretation purposes. The factor loadings were all greater than 0.59, and some loadings were greater than 0.70 (see Table 3). Some studies have utilized the Fornell and Larcker (1981) criterion for evaluating convergent validity in addition to looking at the standardized factor loadings (for instance, Yu et al., 2021; Zahoor et al., 2022). According to Fornell and Larcker (1981), convergent validity is demonstrated when a latent construct explains at least half of the variance in the indicators it is related to. To indicate the average amount of variance that a construct explains in its indicators relative to the sum variance of its indicators, they suggested using the average variance extracted (AVE).

The value of AVE for the PTC subscale equals the sum of the average square of factor loadings across all of its indicators divided by the number of items. In order to determine whether the items logged under each facet or domain were estimating the same concept, composite reliability (CR) values of 0.7 or higher and average variance extracted (AVE) values of 0.5 or greater were employed ( Hair et al., 2019). Convergent validity, according to Hair et al. (2009), is seen when the CR is greater than the AVE and the AVE is greater than 0.5.

According to the table, all of the AVE and the items’ total standardized factor loading were both greater than 0.59, which is a sign of good convergent validity ( Hair, Sarstedt, Ringle, & Gudergan, 2017). For this reason, the value of each PTC subscale’s composite reliability was higher than the value of the average variance extracted (see Table 4). The cheating on research work and cheating on assignments variables’ AVE values (0.37 and 0.38) are below but around the suggested cutoff limit of 0.50 ( Malhotra, 2019), as shown in Table 4. The construct has acceptable convergent validity if AVE is less than 0.5 but the composite reliability is greater than 0.6 ( Fornell & Larcker, 1981). Exam cheating and library material theft value components show acceptable AVEs of 0.56 and 0.61, respectively, exceeding the 0.5 cutoff and confirming the convergence validity of their latent construct.

**Table 4.** CR, AVE, MSV, and ASV Values for PTC Subscales.

Propensity to Cheat Subscale	CR	AVE	MSV	ASV
Cheating on tests	0.88	0.56	0.37	0.29
Cheating on assignment	0.78	0.38	0.20	0.27
Cheating on research work	0.80	0.37	0.41	0.31
Theft and Mutilation of library Material	0.92	0.61	0.37	0.33

Note. CR = composite reliability; AVE = average variance extracted; MSV = maximum shared squared variance; ASV = average shared square variance.

Convergent validity is also demonstrated by the fact that the Maximum Shared Variance for all three variables is less than the pertinent Average Variance Extracted. Our variables have a high level of internal consistency, as seen by the table’s composite reliability of all factors, which is greater than 0.70 ( Gefen et al., 2000).

### 4.2 Discriminant validity of the propensity to cheat scale

The first condition for discriminant validity is establishing convergent validity ( Bagozzi, 1981). This means ensuring that a construct is sufficiently represented by its indicators.

The objective of this stage was to establish further evidence based on relationships to other variables, i.e., evidence pertaining to the construct validity of the instrument ( Balkin, 2017). Construct validity is a vital aspect of validating a tool, as it includes establishing the theoretical link between the variables being measured and other relevant constructs ( DeVellis, 2016). One of the five main forms of validity evidence that focuses on showing connections between assessment scores and crucial criteria is evidence based on linkages to other variables (AERA, APA, & NCME, 2014). The strength and direction of the association between theoretically pertinent constructs are determined using discriminant validity, a kind of construct validity ( DeVellis, 2016). The degree to which there are insignificant correlations between measures of theoretically unrelated constructs is referred to as discriminant validity. Discriminant validity is demonstrated, in accordance with Gefen and Straub (2005), “when each measurement item weakly correlates with another construct other than those to which it is theoretically associated.”

Discriminant validity is conducted to ensure that the PCS measures a construct that is distinct from other constructs. Scales that measure theoretically unrelated constructs should, according to discriminant validity, have a low correlation ( DeVellis, 2003; DeVon *et al*., 2007). Discriminant validity testing is done primarily to demonstrate how different an item or set of items is from others in this study. In other words, the objective of this study is to demonstrate low correlations between items measuring various constructs or variables. This study examined the association between the PCS and other established measures, such as the academic self-concept scale. By assessing these associations, the researchers purpose to reveal the unique contribution of the PCS in measuring PTC in academic settings.

The perceived propensity of students to cheat and their academic self-concept were examined using a bivariate correlational analysis. Bivariate correlation was also used to assess the link between academic achievement and students’ perceived PTC. For the purposes of data analysis, the continuous composite scores on all scales were regarded as interval levels.

Based on a review of the literature, it was predicted that there would be little link between the PTC and academic self-concept. Table 5 displays the relationships between the PTC subscales (MOL, COT, COR, and COA) and academic self-concept (ASC), with the bolded red items denoting discriminant validity. The correlation between the ASC and the subscales (MOL, COT, COR, and COA, respectively) was found to be lower than the correlation between the items of the same construct, such as the respective PTC subscales for MOL, COT, COR, and COA were.678,.741,.586,.520,.571, and.578. This indicates a strong link between items on the PTC subscale alone, but weak correlations between those same items and academic self-concept. To sum up, the results indicate that there is a low relationship between the PTC and academic self-concept, as expected.

**Table 5.** Bivariate Correlations between Academic Self-Concept, PTC Scores, and their Respective Subscales.

PTC Subscales and Academic Self-concept	MOL	COT	COR	COA	ASC
MOL	1				
COT	.678 **	1			
COR	.741 **	.586 **	1		
COA	.520 **	.571 **	.578 **	1	
ASC	.067	.051	.088 *	.121 **	1

Note. MOL = Mutilation of Library Material, COT = Cheating on Test, COR = Cheating on Research Work, COA = Cheating on Assignment, ASC = Academic Self-concept.

** p < 0.01.

* p < 0.05.

Furthermore, research on cheating has shown that there is only a weak negative correlation ( Cizek, 1999) between cheating and grade point average (GPA). Higher GPA students are less likely to report or cheat, but students with lower grades are more likely to do both. For this reason, Table 6 displays that the correlations between the students’ cumulative grade point averages (CGPA) and their PTC subscales (MOL, COT, COR, and COA) are lower compared to the correlations between the items of the same construct (PTC subscales). This indicates discriminant validity, which is represented by the red-bolded items, as the relationships between the CGPA and the PTC subscales are not as strong as the relationships within the PTC subscales themselves. Table 6 indicates that there is a lower negative relationship between the CGPA and the MOL, COT, COR, and COA subscales than there is between the items of the same construct, such as the PTC subscales, which have correlations of .678, .741, .586, .520, .571, and .578 (MOL, COT, COR, and COA, respectively).

**Table 6.** Bivariate Correlations between Cumulative Grade Point Average, PTC Scores, and their Corresponding Subscales.

PTC Subscales and Cumulative Grade Point Average	MOL	COT	COR	COA	CGPA
MOL	1				
COT	.678 **	1			
COR	.741 **	.586 **	1		
COA	.520 **	.571 **	.578 **	1	
CGPA	-.004	-.043	-.106 *	-.055	1

Note. MOL = Mutilation of Library Material, COT = Cheating on Test, COR = Cheating on Research Work, COA = Cheating on Assignment, CGPA = Cumulative Grade Point Average.

** p < 0.01.

* p < 0.05.

In short, the PTC and academic self-concept showed a positive, although weak, relationship, indicating discriminant validity. In addition to this, the PCS’s discriminant validity is supported by the low, negative correlations between students’ academic achievement and PTC.

## 5. Discussion

### 5.1 Confirmatory factor analysis and model fit

A CFA was conducted for this purpose in order to strengthen the validity of the suggested model of PTC and validate the 4-factor solution obtained from the EFA. Several model fit indices were used to assess the goodness of fit of the CFA model. Brown (2015) divided model fit indices into three categories as a result: “absolute fit, fit adjusting for model parsimony, and comparative or incremental fit” (p. 71). According to Brown (2015), absolute fit indices, which include the chi-square statistic (χ ^2^) and the standardized root mean square residual (SRMR) indices, evaluate model fit at an absolute level. Collier (2020) offers a relative chi-square test, which is the chi-square value divided by the degrees of freedom (χ ^2^/df), as a better method to utilize because the chi-square statistic is more sensitive to sample size. According to Collier (2020), a relative chi-square value (χ ^2^/df) that is considered acceptable may range from 3 to 5. Any number below 3 denotes an excellent model fit, while values between 3 and 5 demonstrate an acceptable level. As a result, the first-order CFA model’s outcomes demonstrated that it had a chi-square statistic (χ ^2^ = 3.877, p = .000).

Parsimony correction indices, sometimes also referred to as absolute fit indices, are the second category of model fit indices ( Brown, 2015). The root-mean-square error of approximation (RMSEA) is the most widely used and advised index in this area, according to Brown (2015). The RMSEA is referred to as an error of approximation index since it measures how well a model matches the population ( Brown, 2015). The purpose of this study is not to determine whether the model applies to the population exactly. The RMSEA is hence “sensitive to the number of model parameters” but “relatively insensitive to sample size” ( Brown, 2015, p. 71). Comparative fit indices, which are also referred to as incremental fit indices and assess the fit of a user-specified solution against a nested baseline model, are the third category of model fit indices, according to Brown’s (2015) taxonomy. The CFI and TLI are the most popular and suggested indices in this category, according to Brown (2015).

In this study, the models were assessed to determine if they accurately reflected the data using a number of model fit indices. The measurement model outputs of the first-order CFA model-based fit indices in this analysis were CFI = .916, TLI = 0.907, SRMR = .045, and RMSEA = .076; these values suggest that the model had a good fit to the observed data (χ ^2^/df = 3.88, p = .000). In regard to this, the CFI value was 0.92 indicated a satisfactory fit, whereas CFI values are 0.90 and higher as considered acceptable ( Kline, 2023; Thompson, 2004). RMSEA = .077, CFI = .913, RMR = .049, and TLI = .904 are the results of the second-order CFA model run on the scale structure consisting of four factors and 26 items (χ ^2^/df = 3.966, p = .000). The PCS has a valid and reliable factor structure as a result of the structural model’s adequate fit to the sample data. Thus, both the structural and measurement models demonstrated good fit to the empirical data, indicating that the PCS has a valid and reliable factor structure.

### 5.2 Construct validity assessment of propensity to cheat scale

Discriminant validity and convergent validity were used to investigate construct validity. Convergent validity is usually examined first, followed by discriminant validity. According to Collier (2020), this type of construct validity describes the extent to which all indicators of a certain construct are measuring the same thing (construct) that they are supposed to measure. “Evidence that different indicators of theoretically similar or overlapping constructs are strongly interrelated” ( Brown, 2015, p. 2) serves as the basis for this determination.

The term “convergent validity” refers to the closely connected variables inside a single component ( Kaplan & Saccuzzo, 1993). The convergent validity was investigated using factor loading, average variance extracted (AVE), and composite reliability (CR) ( Hair et al., 2017). All factor loadings were higher than 0.59, as shown in the result section. The standard for an item to be included within a factor is having factor loads above .40 ( DeVellis, 2014; Hair, 2009). In addition, if the factor loading was greater than 0.5, it was considered acceptable ( Hidayat et al., 2018). The AVE abbreviation stands for the “average of the squared standardized pattern coefficients for indicators that depend on the same factor but are specified to measure no other factors” ( Kline, 2023, p. 313). All CR was greater than.85, while AVE was over 0.37.

Accordingly, Hair et al. (2010) proposed that convergent validity is achieved when composite reliability is more than average variance extracted (AVE), with both values exceeding .70 and .50, respectively. The AVE values for plagiarism and theft of library resources are 0.56 and 0.61, respectively, which are both higher than the cutoff value of 0.5 and indicate the convergence validity of their latent construct. However, the AVE values for plagiarism in research work and cheating on assignments are slightly below but near the recommended cutoff of 0.50, at 0.37 and 0.38 ( Malhotra, 2019). The construct has adequate convergent validity if the mean variance is less than 0.5 and the composite reliability is greater than 0.6 ( Fornell & Larcker, 1981). In light of this, all subscales in this study satisfied the convergent validity of the constructs ( Fornell & Larcker, 1981). Therefore, these results demonstrate that the scale has achieved convergent validity.

The test of discriminant validity in this study showed that the PCS, academic self-concept, and CGPA are distinct constructs, as evidenced by the low correlations between them. As demonstrated by Gholami et al. (2013), discriminant validity is reached when a construct measures how many indicators are used to indicate only one construct while being genuinely unique from the other constructs. Two latent variables representing various theoretical concepts are statistically different when they have discriminant validity.

In this study, the correlations between the PCS, academic self-concept, and CGPA were tested. A low negative correlation between the CGPA and the PCS would be indicative of discriminant validity. Similarly, it was supposed that evidence of discriminant validity would come from a low correlation between academic self-concept and PCS. The correlation between the PTC subscale, academic self-concept, and CGPA is less than 0.12, as indicated in the results section, indicating that discriminant validity was demonstrated. The findings of this study align with previous research suggesting that Comas-Forgas and Sureda-Negre (2010) suggest that students who struggle academically are more likely to commit plagiarism. As one of the most significant types of PTC, plagiarism, this evidence can be used to demonstrate the validity of the discriminant. Similarly, studies have indicated that students who perform well academically or have higher cumulative grade point averages are less likely to plagiarize ( Guo, 2011).

Based on the CFA’s findings, the updated versions of the PCS’s four subscales had high support for the scale’s reliability, discriminant validity, and convergent validity. This study provides evidence that the PCS is a valuable instrument for measuring different aspects of PTC.

## 6. Conclusions and practical implications

In this study, a validated assessment scale known as the student’s PCS was developed. This scale may be beneficial to assess students’ intent to cheat and to enable further intervention for the widespread practice of cheating in universities. On the basis of examining the fit index values obtained from the first-order CFA, the scale can be considered to have adequate fit values (χ ^2^/df = 3.877, RMSEA = .076, CFI = .916, SRMR = .045) and TLI = 0.907, whereas in the second-order CFA, the scale can be considered to have adequate fit values (χ ^2^/df = 3.966, CFI = 0.913, TLI = 0.904, RMSEA = 0.077, and RMR = 0.049).

In this study, the convergent and discriminant validity of the four PCS factors’ were examined. Convergent validity was assessed based on the factor loading, extracted average variance (AVE), and composite reliability (CR) values of the scale. The AVE values for the factors were as follows: The first factor had a value of 0.61, the second factor had a value of 0.56, the third factor had a value of 0.38, and the fourth factor had a value of 0.37, the CR values were .92, .88, .78, and .80 for the first, second, third, and fourth factors respectively. The scale demonstrated acceptable convergent validity based on these results.

Discriminant validity was assessed by exploring the relationship between the PTC subscale scores, academic self-concept, and grade point average (GPA). In connection to this, the correlation coefficients between the PTC subscales, students’ academic self-concept, and cumulative grade point average (CGPA) were, respectively, (.678, .741, .586, .520, .571 & .578), (.067, .051, .088 & .121), and (.004, .043, .106 & .055). It was observed that there were low correlations between the PTC subscale items, academic self-concept, and GPA. However, there were significant inter-factor correlations within the PTC subscale items.

Based on the findings, the discriminant validity of the PCS was confirmed. Post-analysis, it is clear that the scale’s convergent and discriminant validity were both supported by the data.

The reliability of the scale was examined using Cronbach’s alpha reliability coefficients. Cronbach’s alpha coefficients were calculated for the scale (total score) and subscales of the PTC in order to evaluate the internal consistency. The scale has a high level of internal consistency, as seen by its overall Cronbach’s alpha (.96) ( Field, 2013). The PTC subscales of cheating on tests, cheating on assignments, cheating on research, and theft and mutilation of library materials all had reliability analyses that were, respectively, .93, .85, .90, and .96.

All four factors of PTC have demonstrated satisfactory validity in terms of convergent validity, discriminant validity, and model fit in the final PCS. A high degree of estimation reliability has also been demonstrated by the PCS.

In conclusion, this study has established the multidimensional character of the concept of PTC. The final PCS now includes 26 and 4 reliable and valid items and factors that may be used to evaluate various PTC aspects.

According to the study’s practical implications, academic measurement and evaluation researchers and practitioners can measure the extent of four common PTC types in Ethiopian higher education contexts, especially among university students, using the PCS. This could help professionals accurately assess issues of academic misconduct related to PTC. The PCS was designed and validated because of the study’s contributions to our understanding of academic and professional environments, cheating behavior, and prevention and intervention measures.

## 7. Limitations and recommendations for future research

Although this study offers academics, practitioners, and students working in the measuring and assessment sector practical advantages, it also has significant limitations that future researchers should address,

Ethiopian public universities serve as the setting for the development and validation of this PCS. Therefore, we suggest researchers at other Ethiopian private universities to examine the validity and reliability of our instrument in relation to their respective academic environments.

Moreover, the purpose of the recently developed and validated instrument was initially to assess a student’s PTC during assessments or examinations. Unfortunately, the study did not include this demographic data (family status and socioeconomic status). It is believed that these factors may have an influence on an individual’s PTC. Lastly, in order to measure PTC effectively, researchers and educators need more valid and reliable tools to study and assess PTC.

## Consent of participants

To begin the process of obtaining participants’ willingness to participate in the study, the researchers introduced themselves and provided further explanations about the study. This included details about the participants’ degree of involvement, the objective of the study, the focus of the questionnaire, and the potential benefits of the research for society and individuals.

After receiving verbal consent from the participants, the researchers explained that participation was voluntary. Participants were informed that they could choose to leave the study at any time without facing any penalties or losing any advantages. Additionally, participants were assured that all information provided would be kept confidential.

Following this verbal consent, written informed consent was then obtained from all participants. The written consent form is available in the data repository ( Gizaw, 2024). This process ensured that participants understood and agreed to their participation in the study, while also emphasizing the importance of confidentiality and voluntary participation. Consent of participants available in the data repository ( Gizaw, 2024).

### Ethical issues

The study was conducted in compliance with pertinent standards and regulations and was approved by Hawassa University. After the PhD dissertation proposal was approved and ethical clearance was obtained from Hawassa University’s Office of Vice President for Research and Technology Transfer (VPRTT), the study was carried out. The School of Teacher Education granted approval in this regard on 09/02/2014, with reference number COE-REC/017/24. The data repository contains the ethical approval certificate ( Gizaw, 2024).

## Data Availability

OSF: Propensity to Cheat and Academic Self-concept. https://doi.org/10.17605/OSF.IO/7G9JN ( Gizaw, 2024) This project comprises the following: •
**
Figure 1. JPEG** output of the measurement model for Latent Variable "Four-Factor of PTC" and Observed Indicators •
**
Figure 2. JPEG** output of the Structural Model for the Students’ Propensity to Cheat. • SPSS data.xlsx OSF: Data and materials for propensity to cheat scale. This project comprises the following:
- Questionnaire.docx - Informed Consent.docx - Participation Consent Form - Permission Certificate - Ethical Approval Certificate - Acknowledgement Certifiicate Data are accessible under the terms of the Creative Commons Attribution 4.0 International license (CC-BY 4.0)
