# Confirmatory Factor Analysis and Propensity to Cheat Scale Validation in the Ethiopian Public Higher Education Institutions

**DOI:** 10.12688/f1000research.150357.4

**Published:** 2025-08-29

**Authors:** Dame Taye, Tesfaye Semela, Samuel Assefa

**Affiliations:** 1 School of Teacher Education, College of Education, Hawassa University, Hawassa, Hawassa, 8090, Ethiopia; 2 School of Teacher education, College of Education and Institute of Policy & Development Research (IPDR), Hawassa University, Awassa, Southern Nations, Nationalities, and People's Region, 8090, Ethiopia; 3 School of Teacher Education, College of Education, Hawassa University, Awassa, Southern Nations, Nationalities, and People's Region, 8090, Ethiopia

**Keywords:** exam cheating; propensity to cheats; university students; confirmatory factor analysis; construct validity

## Abstract

**Background:**

This study aimed to validate a comprehensive and psychometrically sound instrument—the Propensity to Cheat Scale (PCS)—designed to measure undergraduate students’ propensity toward academic dishonesty in Ethiopian universities. Based on Ajzen’s Theory of Planned Behavior, the PCS was validated to assess students’ attitudes, subjective norms, and perceived behavioral control related to various forms of cheating, including cheating on tests and examinations, cheating on assignments, cheating on research work (plagiarism), and theft and mutilation of library materials.

**Methods:**

The present study employed an explanatory research design using a questionnaire based on the Propensity to Cheat Scale (PCS). The questionnaire was administered to 500 university students (male = 367 [73.4%]; female = 133 [26.6%]) selected from three Ethiopian public universities between November and January 2022. In order to measure the underlying variables of propensity towards cheating, a factor model is developed using exploratory factor analysis (EFA), and confirmatory factor analysis was employed to validate the students’ perceived PTC. The internal consistency of the PTC scale was assessed using reliability analysis, and validity evaluations were conducted to confirm the scale’s discriminant and convergent validity.

**Results:**

Confirmatory factor analysis (CFA) results revealed a good fit to the data, and the internal consistency of the PCS was found to be strong, providing a reliable measure of students’ propensity for cheating. Validity evaluations, including discriminant validity and convergent validity, confirmed the validity of the scale. The average variance extracted (AVE) and composite reliability values also supported the scale’s convergent validity. The multidimensional concept of the PTC was supported by a four-factor solution consisting of 26 reliable and valid items.

**Conclusion:**

The findings of the study demonstrate that the scale has also provided sufficient evidence of convergent and discriminant validity. By establishing discriminant and convergent validity, as well as reliability, through different validation procedures, the study has provided strong evidence for the effectiveness of the PCS as an instrument for determining whether university students are likely to engage in cheating behavior.

## 1. Introduction

### 1.1 Background of the study

Academic dishonesty is a broad term that encapsulates various detrimental behaviors within educational environments, such as plagiarism and falsification of information. Although difficult to define precisely ( Lambert et al., 2003), academic dishonesty involves a variety of damaging activities, such as plagiarism or making false excuses ( Yazici et al., 2011). The concept of academic cheating was initially described by Rich (1984) as deceitful actions related to coursework, including cheating on tests, exams, and assignments, along with activities like obtaining exams illegally, plagiarizing content, manipulating data, and tampering with library resources.

According to Davis et al. (2009), academic cheating involves deceiving or defrauding another’s through misleading or dishonest actions. They emphasize that it entails students engaging in behaviors that trick instructors into believing that the academic work submitted is their original creation. Academic cheating can be defined as “intentionally unethical behaviour” ( Von Dran et al., 2001, pp. 40) and “using deception (fraud) in academic work” ( Cochran, 2016, pp. 814), both of which result in a breach of the defined rules and accepted standards, granting cheaters an unfair benefit over those who do not cheat ( Dick et al., 2003). ensuring quality outcomes. In order to guarantee quality outcomes, student assessment should actually be seen as a complex and multifaceted activity that requires alignment, balance, and rigor ( Joughin & Macdonald, 2004). Academic integrity is paramount to upholding the quality of education, and reducing academic fraud is essential for a thorough evaluation process.

Academic dishonesty by students, which this study defines as plagiarism and cheating on assignments and tests ( Adesile et al., 2016; Ives et al., 2017; Ives and Nehrkorn, 2019; Jensen et al., 2002; Lin and Wen, 2007; Owunwanne et al., 2010), undermines the goals of higher education institutions. It compromises assessment validity since results cannot be generalized to represent trustworthy estimates of students’ grasp of course-work content ( Bouville, 2010; Muñoz-García and Aviles-Herrera, 2014).

The validation of the Propensity to Cheat Scale (PCS) is grounded in social-cognitive theories, particularly Ajzen’s (1991) Theory of Planned Behavior (TPB). This theory posits that an individual’s behavioral intentions—and ultimately their actions—are shaped by three key factors: attitudes toward the behavior, perceived social norms, and perceived behavioral control. Within the context of academic dishonesty, TPB offers a robust framework to understand why students may engage in cheating, especially when their academic self-concept is low ( Ajzen, 1991; Stone et al., 2010). Applying TPB to Ethiopian higher education helps illuminate the social and psychological drivers of academic misconduct. The theory emphasizes that a student’s decision to cheat is not solely based on personal inclination, but also significantly influenced by cultural norms and perceived expectations. For instance, when students believe that “everyone cheats” (subjective norm) and that the risk of getting caught is minimal (high perceived behavioral control), they are more likely to form the intention to cheat—even if they initially hold negative attitudes toward dishonest behavior ( Stone et al., 2010). This insight underscores the importance of situating academic dishonesty within a broader theoretical framework that incorporates both individual cognition and contextual influences. Thus, integrating TPB into the study provides a structured lens to examine the multidimensional nature of students’ cheating tendencies.

The topic of propensity to cheat (PTC) among students is a significant concern in higher education institutions. A student’s propensity is the likelihood that they will engage in a specific action or behavior. Propensity is defined in the literature as the tendency or predisposition to participate in a particular behavior, in this case, cheating. Cheating tendencies are the inclinations of an individual or a group of individuals to commit crimes prior to, during, or following an exam with the intention of unfairly benefiting from an unfair advantage over other students ( Offor, 2009). The Propensity to Cheat (PTC) refers to the likelihood that students will engage in dishonest behaviors to gain unfair advantages in academic settings. This propensity is measured by the variety of self-reported cheating methods that students have employed, indicating their tendency towards such behaviors. PTC encompasses a range of unethical practices, including cheating on exams, plagiarizing assignments, falsifying research data, and misusing library resources. It highlights the broader issue of academic dishonesty, which not only includes cheating during exams but also extends to dishonest actions related to assignments, research work, and the misuse or theft of educational materials.

The tendency to cheat is closely related to academic dishonesty, which encompasses various unethical behaviors in educational settings. When individuals are more inclined to cheat, it often indicates a higher likelihood of engaging in dishonest practices that undermine the integrity of their academic efforts. Cheating can take many forms, such as plagiarism—where students copy another person’s work without giving proper credit—and falsification of information, which involves altering or fabricating data or sources to misrepresent one’s work or findings ( McCabe and Trevino, 1996). For the objectives of this study, understanding this broader concept of cheating propensity is essential for developing effective strategies to prevent academic misconduct.

The study intended to validate the Propensity to Cheat (PTC) scale and examine its relationship with students’ Cumulative Grade Point Average (CGPA) and academic self-concept. The PTC serves as a measure of students’ likelihood to engage in academic dishonesty, and understanding its correlation with academic performance and self-perception is crucial for addressing issues of academic integrity.

Recent literature has explored how students’ academic self-concept and academic performance (often operationalized as GPA) relate to their propensity to cheat. Academic accomplishment is one of the most often utilized constructs in educational research and assessment in higher education. Academic achievement is usually measured by students’ grades and overall performance in their courses. CGPA is a commonly used measure of student’s academic performance in higher education, reflecting their overall academic achievement across all semesters. In this study, CGPA was used as an indicator of academic achievement.

Some academics believe that academic cheating is linked to a student’s GPA. The study highlighted the importance of academic achievement as a determinant of students’ propensity to cheat. For example, academic dishonesty was negatively related to students’ GPA. Those with higher GPAs, according to Tang and Zuo (1997), are less inclined to cheat than students with lower GPAs. The researchers also expected that GPA and self-esteem have an interaction. Students who have a high GPA and a high sense of self-worth are less prone to cheat.

To put it another way, students with a high GPA are less likely to cheat, whereas students with a low GPA are more likely to cheat ( Baird, 1980; Lipson & McGavern, 1993; Olafson et al., 2013). McCabe et al. (2012) found evidence that students who looked to be particularly competitive at the top of the rankings also showed a higher likelihood of cheating. In this study, CGPA was used to assess the discriminant validity of the measurement of propensity toward academic misconduct.

Self-concept is a multidimensional construct with one general facet and numerous specific facets, one of which is “academic self-concept.” The multi-dimensional model of self-concept shows that, along with social, emotional, and physical self-concept, intellectual self is one of the essential elements of self that contribute to an individual’s global self-concept ( Marsh, 2006). Furthermore, this multidimensional model of self-concept distinguishes between general self-concept, which encompasses cognitive, affective, and behavioural elements, and academic self-concept, which refers to a person’s view of their academic competence ( Bandura, 1993).

Academic self-concept refers to students’ perceptions of their academic abilities. It plays a significant role in their learning and academic performance. Academic self-concept, according to Trautwein et al. (2006), is an individual’s assessment of their academic ability. Academic self-concept is described as students’ assessment of their competency, dedication to, involvement in, and interest in classroom work, according to a study by Liu et al. (2005).

According to some scholars, academic misbehavior is linked to university students’ academic self-concept. Self-concept has an impact on examination misconduct perception, according to a study that looked into the relationship between students’ academic self-concept and examination misconduct perception ( Marsh, 1990; Anderman & Murdock, 2007). Students with low self-esteem are more likely to engage in risky behaviors, such as cheating on exams, according to Kehinde (2004), whereas achievement-related beliefs, such as academic self-concept, will almost certainly influence students to engage in academic cheating behaviors, according to Rinn et al. (2014). In summary, multiple studies have established a relationship among GPA, academic self-concept, and the tendency to cheat, although the evidence remains mixed.

In this study, academic self-concept was evaluated to measure students’ confidence and motivation regarding their academic abilities. To assess the discriminant validity of the measurement concerning the propensity toward academic misconduct, we utilized an academic self-concept scale. This approach allowed us to examine the relationship between students’ self-perception of their academic capabilities and their tendencies toward engaging in dishonest academic behaviors. By ensuring that the academic self-concept scale is distinct from measures of academic misconduct, we aimed to establish a clear differentiation between positive self-perception in academics and the likelihood of misconduct.

### 1.2 Limitations of existing instruments and research gap

While academic dishonesty has long been recognized as a global concern, many existing measurement instruments—primarily developed in Western contexts—fail to reflect the socio-cultural realities of academic misconduct in Ethiopian public universities. These tools often emphasize overt, individualistic cheating behaviors and overlook culturally embedded practices such as communal cheating and context-specific forms of dishonesty. Consequently, they lack the cultural sensitivity required for valid measurement in non-Western educational systems.

Scholars such as Hair et al. (2010) and DeVellis (2016) emphasize the importance of developing culturally adapted and psychometrically robust instruments tailored to specific educational contexts. This highlights the need to create and validate the Propensity to Cheat Scale (PCS) for use in Ethiopian higher education. Specifically, validation efforts should focus on establishing both convergent validity—ensuring that the scale accurately measures the construct of cheating propensity—and discriminant validity—demonstrating that the scale distinctly measures this construct apart from related variables such as academic self-concept and GPA.

Moreover, many widely used instruments rely solely on exploratory factor analysis (EFA), without employing robust confirmatory methods. This practice weakens the evidence base for construct validity, leaving substantial gaps in psychometric rigor ( Hair et al., 2010). Highlighting the relationships among academic self-concept, cumulative GPA (CGPA), and cheating behavior using updated literature can further enrich the theoretical foundation of such instruments.

### 1.3 Rationale for a Locally Developed Propensity to Cheat Scale (PCS)

Psychometric validation plays a central role in ensuring that research tools are both reliable and contextually relevant. Recent African-based studies (e.g., Faye et al., 2024; Padmanabhanunni & Pretorius, 2024; Ford et al., 2025) have strongly advocated for culturally aligned validation procedures in educational and psychological assessment. These findings affirm the relevance of developing a PCS tailored specifically for the Ethiopian university setting.

A locally validated PCS offers several advantages over imported instruments. First, it ensures that scale items are culturally grounded and reflect the realities of students’ experiences and attitudes. Second, anchoring the scale in Ajzen’s (1991) Theory of Planned Behavior provides a robust conceptual framework by capturing the role of attitudes, perceived norms, and behavioral control in shaping students’ propensity to cheat. This theoretically informed structure enhances the scale’s construct validity by directly linking it to well-established psychological constructs and separating it from confounding variables like self-concept and GPA.

Convergent and discriminant validity are fundamental components of construct validity, originally conceptualized by Cronbach and Meehl (1955) and further operationalized by Campbell and Fiske (1959). In the current study, the Propensity to Cheat (PTC) scale was assessed for convergent validity by analyzing the internal consistency of its indicators measuring the same latent construct. Convergent validity refers to the extent to which multiple items intended to assess a common construct exhibit strong inter-correlations, thereby forming a cohesive and theoretically meaningful factor. High internal consistency among these items indicates that the scale reliably captures the intended construct ( Cronbach & Meehl, 1955; Campbell & Fiske, 1959).

In Bagozzi’s (1981) definition of convergent validity, the emphasis is on the internal consistency of indicators measuring the same construct. Researchers utilized reliability measures as one of the criteria to assess convergent validity ( Fornell & Larcker, 1981). However, they recognized that evaluating convergent validity just on the basis of reliability is insufficient. To further assess convergent validity, a measurement model was estimated in which all indicators were related to the constructs they were intended to measure and not directly related to constructs they were not intended to measure ( Fornell & Larcker, 1981; Hair et al., 2009). All indicators converge well on their own construct when the predicted measurement model sufficiently matches the data. Since model fit does not ensure measurement quality, researchers have argued that an appropriate model fit is insufficient to support convergent validity ( Fornell & Larcker, 1981). As a result, additional criteria have been proposed to ensure that the indicators truly measure the intended construct.

The first condition for discriminant validity is establishing convergent validity ( Bagozzi, 1981). This means ensuring that a construct is sufficiently represented by its indicators.

The objective of this stage was to establish further evidence based on relationships to other variables, i.e., evidence pertaining to the construct validity of the instrument ( Balkin, 2017). Construct validity is a vital aspect of validating a tool, as it includes establishing the theoretical link between the variables being measured and other relevant constructs ( DeVellis, 2016). One of the five main forms of validity evidence that focuses on showing connections between assessment scores and crucial criteria is evidence based on linkages to other variables (AERA, APA, & NCME, 2014). The strength and direction of the association between theoretically pertinent constructs are determined using discriminant validity, a kind of construct validity ( DeVellis, 2016). The degree to which there are insignificant correlations between measures of theoretically unrelated constructs is referred to as discriminant validity.

Discriminant validity evaluates the extent to which a measurement instrument distinguishes between constructs that are theoretically unrelated. It serves to confirm that the scale does not inadvertently measure concepts outside the intended construct domain, thereby safeguarding against construct contamination ( Cronbach & Meehl, 1955; Pike, 1992, 2006).

Discriminant validity is demonstrated, in accordance with Gefen and Straub (2005), “when each measurement item weakly correlates with another construct other than those to which it is theoretically associated.” In summary, discriminant validity is established when measures of theoretically unrelated constructs exhibit low correlations, thereby confirming that the instrument assesses a distinct construct. For example, a weak relationship between the Propensity to Cheat Scale (PCS) and academic self-concept supports the claim that the PCS is not capturing unintended dimensions. Simultaneously, the evaluation of convergent validity ensures that all items designed to measure propensity to cheat consistently reflect a single underlying construct. Together, these assessments help confirm the precision and construct validity of the PCS ( Cronbach & Meehl, 1955; Campbell & Fiske, 1959).

### 1.4 Prevalence of academic dishonesty

Various studies indicate that academic dishonesty is more prevalent than ever, and actions need to be taken by universities to educate students as well as faculty members about academic integrity and ethical professionalism ( Diego, 2017; Feday, 2017; Jepngetich et al., 2017; Mebratu, 2014; Nelson et al., 2012). According to studies, dishonest behavior that began in high school can continue in college, and similarly, college students who engage in academic dishonest behavior are more likely to engage in dishonest behavior outside of the classroom ( Harding et al., 2004). This suggests that there is a chance that dishonest behavior will continue over time and in different circumstances.

Academic cheating is a widespread problem around the world, with high rates of cheating reported among students in many countries. In the United States, surveys have revealed that up to 86 percent of college students have engaged in dishonesty in the classroom ( McCabe et al., 2006). Bernardi et al. (2008) conducted a study to compare the perspectives of students from industrialized countries on cheating behavior. They discovered that 51% of the people in the samples admitted to cheating. According to Yussof and Ismail (2018), high rates of Malaysian students have cheated, primarily in assignments and quizzes that carry a lower weight in the final grade and are subject to less supervision and punishment. In South Korea, Ahmed (2018) discovered that 65 percent of pupils misbehaved using electronic media, and 80 percent of students committed academic fraud.

In Africa, academic dishonesty is equally prevalent. According to Umar (2004), cheating on exams was almost a standard practice in Nigeria. According to Munachonga (2015), examination misconduct has become increasingly common in Ghana. This is mostly because candidates are afraid of failing, lack confidence, are lazy, don’t prepare well, and, most importantly, are unable to apply themselves to their studies. Munachonga (2015) noted that officials of the Test Council of Zambia, the nation’s supervisory organization for exams, have occasionally been involved in examination malpractices.

When compared to various other studies carried out worldwide, the prevalence rate found in Ethiopia is high. Even though there is little to no evidence on the subject, cheating has from time to time increased in Ethiopia ( Tefera & Kinde, 2009). Chala (2021) discovered that, with an incidence a rate ranging from 53 to 96 percent, academic dishonesty is a severe problem impacting university students in Ethiopia. Research conducted locally has, in one form or another, verified the growing epidemic of academic cheating in Ethiopian education at all levels ( Feday, 2017; Hailu, 2015; Mebratu, 2014; Tefera & Kinde, 2009; Wondifraw, 2021). Furthermore, according to the Ethiopian Education Development Roadmap (2018–30), students frequently cheat on tests, and teachers are afraid that aggressive student evaluation may lead to an inaccurate assessment of their work and inflated grades ( MoE, 2018).

Academic dishonesty can be justified in various ways in an academic setting. The majority of the time, in Ethiopia, the behaviors take the form of plagiarism and exam cheating (papers and assignments). To mention a few results of research done in the Ethiopian setting, Feday (2017) and Mebratu (2016) found that plagiarism in written work and exam cheating are the most common forms of academic dishonesty. Furthermore, several lecturers have expressed concern about the increasing incidence of plagiarism, assignment cheating, and exam dishonesty in Ethiopia’s public universities. This suggests that these universities share the researchers’ concerns based on their observations and teaching experiences. Specifically, some students at Hawassa University and Wolkite University College of Science and Engineering have been found to engage in academic dishonesty, as reported by researchers with several years of teaching experience in these courses. Furthermore, some students oppose the growing practice of cheating on exams and plagiarism in coursework. Based on our observations, a major contributing factor to exam cheating is a student’s lack of academic proficiency in a particular area. In this sense, the chance of earning a high cumulative grade point average (CGPA) is increased by cheating. Closing the gap and validating a scale that might possess the required psychometric properties to specify measurement accuracy was, thus, the primary objective of this work.

### 1.5 Problem statements

Numerous measures have been developed to assess university students’ tendencies toward academic dishonesty, as highlighted in a review of previous studies on the subject. Academic cheating is a multidimensional issue that affects individuals globally ( Imran & Nordin, 2013; Iberahim et al., 2013; Nazir & Aslam, 2010; Thomas, 2017; Tadesse & Getachew, 2010; Saidin & Isa, 2013; Yang et al., 2013). Among these measures, the Academic Practices Survey stands out as a two-dimensional construct that encompasses plagiarism—referring to written work—and cheating, which pertains to class tests and exams, as identified by Roig and DeTommaso (1995) and Ferrari (2005). Furthermore, Adesile et al. (2016) introduced a three-dimensional scale for measuring academic integrity that includes plagiarism, cheating, and research misconduct. Given these varying dimensions of academic dishonesty, this study focuses specifically on the dimensions of the Academic Practices Survey, examining cheating on tests, assignments, research work (plagiarism), and the mutilation or theft of library resources.

The main issue with measuring the PTC in environments where academic dishonesty is prevalent is its legitimacy. While a few studies have addressed the PTC, none have utilized a well-developed and validated questionnaire. Furthermore, aside from a brief mention of the reliability coefficient, little is known about the confirmatory component analysis ( Adesile et al., 2016). As Imran and Nordin (2013) noted, the dimensionalities of most measures have not been carefully examined, and there is insufficient evidence to suggest that they possess meaningful psychometric properties. Additionally, discriminant validity—the ability of a scale to differentiate between the relevant concept and other related constructs—is lacking in previous scales. This failure to demonstrate differentiation has led to confusion in interpretation ( Bashir & Bala, 2018). Researchers can address these challenges and recommend further examination into the PTC by assessing the validity and reliability of existing measures.

This study aimed to assess the construct validity of a locally developed Propensity to Cheat Scale (PCS) among science and engineering students, focusing particularly on its convergent and discriminant validity. Establishing a valid and culturally reliable PCS is essential for accurately measuring the constructs related to cheating behavior. To ensure that psychological and behavioral constructs are properly defined and evaluated, it is crucial for instruments to undergo rigorous validation and assessment ( Hair et al., 2010). Additionally, Hair et al. emphasized the importance of continuously confirming the validity and unidimensionality of conceptual frameworks, even when using well-established instruments. In this context, the study sought to analyze the convergent and discriminant validity of the PCS specifically. Although a Multimethods Multitraits (MMMT) investigation was employed to determine these validity measures, there was insufficient evidence to substantiate the findings. Ultimately, this research contributes to the body of data supporting the construct validity of the PCS, highlighting the necessity of validating instruments that measure the constructs of interest.

This study arises from the lack of an instrument to measure the tendency for academic dishonesty in Ethiopian higher education institutions. Despite the researchers’ experience in this field, there has been no prior research on the psychometric qualities and validity of the PCS used by these institutions. To address this gap in the literature, the researchers aimed to evaluate the psychometric properties of the PCS within the context of Ethiopian higher education.

### 1.6 Research questions

The researchers concluded that it would be appropriate to validate the locally developed PCS in order to assess its convergent and discriminant validity. Two distinct research questions were formulated in order to achieve the research objectives:

1. To what extent does the PCS demonstrate convergent validity?
2. To what extent do the items in the instrument of PCS show discriminant validity?

## 2. Methods

### 2.1 Research design

According to Creswell and Plano (2007, p. 58), a research design is the “procedures for collecting, analysing, interpreting, and reporting data in research studies.” It is the overarching strategy for linking the theoretical research issues with relevant (and doable) empirical research. In other words, the study design determines how the necessary data will be collected, how it will be analysed, and how it will be used to address the research question ( Grey, 2014). Three different types of study designs can be used: exploratory, descriptive, and explanatory ( Robson, 2002). His classification system is based on the goal of the research field because each design has a distinct ultimate goal. To understand the relationship between students’ reported tendency to cheat and their academic self-concept and academic accomplishment, this study uses a quantitative approach and an explanatory research design. A correlational research methodology used for explanatory purposes allows the researcher to assess the degree of association between two or more variables. Additionally, using this style of research design enables the researcher to gather data all at once ( Creswell, 2015).

### 2.2 Population of the study

The study focused on fourth-year science and engineering regular undergraduate students attending Ethiopia’s public universities. The researchers selected this specific group from the target population of first- to fifth-year science and engineering undergraduate students in the participating universities. The target population consisted of 6,524 students, including 4,901 males and 1,623 females.

### 2.3 Samples and sampling procedure

In this study, the sample selection was conducted systematically through purposive sampling. Out of a pool of 50 public universities in Ethiopia, three institutions—Hawassa University, Ambo University, and Wolkite University—were deliberately chosen to represent different stages of institutional maturity. Specifically, Hawassa University was selected as an “established” institution due to its early founding in the 1970s, well-developed academic infrastructure, large enrollment in science and engineering disciplines (exceeding 2,946 students), and a strong research tradition. Conversely, Ambo and Wolkite Universities were identified as “newer” institutions, having been established more recently—Wolkite University in 2012 and Ambo University at a similarly recent date. These newer institutions exemplify an evolving academic environment, emerging research initiatives, and rapid expansion of academic practices. The selection process was guided intentionally by expert judgment rather than random sampling. As Airasian and Gay (2003) note, purposive sampling involves selecting institutions based on credibility and reputational factors—such as research output, enrollment size, and institutional history—to ensure the inclusion of entities most likely to yield rich, discriminant data regarding academic misconduct.

 Next, a purposive sampling strategy was applied to select the colleges within the participating universities, focusing on the Colleges of Natural and Computational Sciences and Engineering/Institute of Technology. The College of Sciences and Technology was included in the study due to its significant role in influencing the quality of education. Additionally, these science and engineering colleges were chosen because of a curriculum shift towards a focus on science and technology, aligned with the policy of a 70:30 graduate mix. The emphasis on these fields is connected to social transformations and economic development (Ministry of Science and Technology, 2015-2025). Furthermore, according to the Ethiopian Education Development Roadmap (2017-2030), the curriculum aims to enhance higher-order thinking skills by teaching science, technology, engineering, and mathematics (STEM) subjects at appropriate educational levels.

After selecting the colleges, 30 departments from the engineering college and 17 departments from the College of Natural and Computational Sciences were randomly chosen. This random selection ensured a diverse representation among the departments within the selected colleges. Finally, a simple random selection procedure was employed to choose students from these departments for the study, ensuring that the student sample accurately represented the larger student population within the universities. Of the 550 students invited to participate in the study, only 500 completed the questionnaire correctly. The universities, their respective colleges, and the study participants are summarized in  Table 1.

**Table 1.** Universities, Colleges, and Number of Fourth Year Undergraduate Students Included in the Study.

Name of your University			Gender		
			Male	Female	Total
Ambo University	Name of College/Institute	College of natural and computational science	60	26	86
		Engineering	51	26	77
	Total		111	52	163
Wolkite university	Name of College/Institute	College of natural and computational science	35	16	51
		Engineering	61	14	75
	Total		96	30	126
Hawassa University	Name of College/Institute	College of natural and computational science	64	36	100
		Engineering	96	15	111
	Total		160	51	211
Total	Name of College/Institute	College of natural and computational science	159	78	237
		Engineering	208	55	263
	Total		367	133	500

The researcher converted Gomez’s (2013) Sloven’s Formula into a formula to compute the sample size for this study. The sample size was determined using the following formula. The following is how this formula is laid out:

$$n=\frac{N}{\left(1+\left(N\right)\left(e^{2}\right)\right)}$$

Where, *N* is the target population size

*n* is the sample size and

*e* is the level of precision (acceptable margin of error at 5% (95% confidence level), P = .5 are assumed for equation

For this study the target population ( *N* = 6524) then the required sample size is calculated as

$$N=\frac{6524}{\left(1+6524\right)\;{\left(0.05\right)}^{2}}=377$$

When selecting a sample size for the study, several criteria need to be considered. These include the size of the population, the level of confidence, the margin of error (confidence interval), the number of variables used in the study, the statistical analysis technique to be employed, as well as time, money, and effort constraints. In this regard, Ellen (2014) asserts that a formula must be employed to account for the margin of error and confidence level when a sample is drawn from a population. Further, research claims that Sloven’s formula should be used when there is little information available about how a population will behave (such as in the case of this study polling college students to assess their opinions and likelihood to cheat online), other than its size ( Ellen, 2014; Kavai, 2014; Tanty & Rahayu, 2014; Abun & Cajindos, 2012). This equation enables the researcher to accurately sample the desired population ( Ellen, 2014). It is thought reasonable to select a sample size using Slovin’s formula, which was developed in 1960. This is true in particular when there is uncertainty regarding the behavior of the population ( Isip, 2014).

Moreover, a carefully determined sample size is essential for obtaining reliable results from an exploratory factor analysis (EFA). Researchers have offered varying recommendations: DeVellis (1991) suggests a minimum of 300 cases, whereas Comrey and Lee (1992) classify 100 cases as poor and 500 or more as very good. Bentler and Chou (1987) advocate for a 5:1 participant-to-variable ratio, and Gorsuch (1983) advises that there should be at least five cases per variable. Furthermore, recent work by Hair et al. (2010) and Kline (2016) supports the use of sample sizes ranging between 300 and 500 for factor analysis, particularly when considering the complexity and number of parameters involved. Based on these guidelines, the present study’s sample of 500 student responses is deemed appropriate, ensuring that the derivation of a four-factor solution from 26 items is both statistically robust and methodologically sound.

Of the 550 students invited to participate in the study, only 500 completed the questionnaire correctly. The universities, their respective colleges, and the study participants are summarized in  Table 1.

The validation of the Propensity to Cheat Scale (PCS) followed a structured approach, which included data collection, Confirmatory Factor Analysis (CFA), and validation. The timeline and sample size are illustrated in the workflow chart below.

**Table:** Workflow chart.

Phase	Activity	Timeline	Sample size	Description
Data collection	Questionnaire administered to participants	November 2022 - January 2023	550	Gathering data from sample population.
CFA	CFA Analysis	November 2022 - January 2023	500	Conduct CFA to validate the factor structure.
Validation	Reliability and validity assessments	April 2023 - May 2023	500	Assess internal consistency, discriminant and convergent validity of the PCS.
Final draft	Prepare draft	June 2023 - July 2023	500	The final report was prepared.

### 2.4 Research instruments

The Students’ Perceived PCS was used to measure the students’ views of their propensity for academic misconduct. The measure has thirty items scored on a Likert-type scale with a maximum of five points, ranging from 1 (Strongly Disagree) to 5 (Strongly Agree).

The study focused on the latent construction of students’ propensity for academic misconduct in higher education settings in Ethiopia, examining four dimensions of cheating behavior: (1) cheating on tests and examinations, (2) cheating on assignments, (3) cheating on research work (plagiarism), and (4) theft and mutilation of library materials. A total of thirty items were included in the study, with the distribution as follows: six items measured cheating on exams, eight items assessed cheating on assignments, nine items evaluated cheating on research papers, and seven items addressed stealing and mutilating library materials. Higher scores on each subscale indicated a greater tendency to engage in academic misconduct. The internal consistency reliability of the subscales was established, yielding alpha values of .93 for cheating on exams, .85 for cheating on assignments, .90 for cheating on research work, and.96 for theft and mutilation of library resources.

This study employed a self-administered questionnaire for the measurement of PTC by four first-order factors: theft and mutilation of library materials, cheating on tests or exams, cheating on assignments, and cheating on research work. All of the dimensions and their subdomains have been constructed, and this study included the reliability and validity (convergent and discriminant validity) of the scale. Notion validity in this context refers to how well a scale’s items are suited to measuring a certain theoretical construct ( DeVon et al., 2007; Kane, 2001). Discriminant and convergent validity are two fundamental features of construct validity, according to most arguments ( Cronbach & Meehl, 1955; Campbell & Fiske, 1959; Pike, 1992, 2006). The construct validity of the PCS was then investigated in this study by examining both its convergent and discriminant validity.

To assess academic self-concept, Liu and Wang (2005) developed a scale consisting of 20 items. These items are designed to measure students’ confidence and motivation in their academic abilities within a university environment. Participants rated each item on a five-point Likert scale, ranging from strongly disagree (1) to stronglyagree (5). The internal consistency reliability of this scale is 0.85. The academic self-concept scale was used to assess discriminant validity of the measurement of propensity toward academic misconduct.

**2.4.1 Validation of the measures**

Following the pilot study, which established face validity, content validity, and reliability, further testing of various types of validity and reliability is required before fully implementing the measures and considering them during the PCS validation process. This study involves an adequate number of participants to ensure robustness.

To assess the construct validity and dimensional structure of the PCS, we employed both Exploratory Factor Analysis (EFA) and Confirmatory Factor Analysis (CFA). EFA was initially used to explore the underlying factor structure of the PCS. This involved identifying the number of factors that best represent the data and determining which items load significantly onto each factor. Items that did not load significantly on any factor or that exhibited cross-loading onto multiple factors were removed based on the EFA results.

Study 1 represents the initial phase of the research focused on developing and preliminarily validating the Propensity to Cheat Scale (PCS). This phase included an exploratory factor analysis (EFA) aimed at identifying the underlying factor structure related to students’ propensity to cheat.

The EFA was conducted using the Maximum Likelihood (ML) method for factor extraction, and oblique rotation was applied to permit correlated factors, which is particularly suitable for research involving human behavior. The analysis revealed a four-factor solution, utilizing Maximum Likelihood analysis with Oblimin and Kaiser Normalization rotation.

The identified factors had eigenvalues exceeding one, with the first factor accounting for the majority of variance. Specifically, Factor One had an eigenvalue of 13.42 and explained 46.28% of the variance. Factor Two had an eigenvalue of 2.17, contributing 7.46%; Factor Three had an eigenvalue of 2.02, accounting for 6.98%; and Factor Four had an eigenvalue of 1.22, explaining 4.20%. Collectively, these four factors accounted for an average of 64.92% of the total variance.

The details of the factor loadings and communalities are as follows: Factor One: Comprising seven items (MOL55-MOL61) with loadings ranging from .58 to .93 and communalities from .72 to .87. Factor Two: Comprised six items (COT5-COT11) with loadings between .61 and .81 and communalities from .60 to .73. Factor Three: Included seven items (COR45-COR52) with loadings from .51 to .71 and communalities ranging from .41 to .69. Finally, Factor Four: Consisted of six items (COA22-COA30) with loadings between .52 and .66 and communalities from .37 to .53.

Subsequently, CFA was conducted to test the hypothesized factor structure identified in the EFA. This step involved specifying a model based on the EFA findings and evaluating the model’s fit to the data. To assess model fit, we utilized goodness-of-fit indices such as the Comparative Fit Index (CFI) and the Root Mean Square Error of Approximation (RMSEA).

The final step in the validation process involved confirming that the model fits the data well and that the items reliably measure the intended construct. This assessment also included evaluating the internal consistency of the scale using Cronbach’s alpha. Through this comprehensive approach, we aimed to ensure that the PCS is a valid and reliable measure for our study.

In a validation study, 550 students from three public universities were given a questionnaire to determine their PTC and their perception of their academic self-concept. Out of the initial 550 participants, 500 students fully answered the PTC and academic self-concept items and were included in the analysis.

In this study, questionnaire responses were carefully reviewed, and incomplete responses (50 out of 550) were removed via listwise deletion, resulting in 500 complete cases for all subsequent analyses. This approach ensured that each statistical procedure—whether Exploratory Factor Analysis (EFA) or Confirmatory Factor Analysis (CFA)—was conducted on a consistent, complete dataset. The minimal proportion of missing data met the assumption of Missing Completely at Random (MCAR), meaning that the likelihood of a response being missing was unrelated to either observed or unobserved variables. Given that the missing data per item were less than 10%, listwise deletion was considered appropriate and did not introduce systematic bias ( Allison, 2001; Little & Rubin, 2019). Consequently, this method assured that the validation of the Propensity to Cheat Scale (PCS) was based on a robust and coherent dataset, thereby maintaining the study’s overall methodological rigor.

The gender distribution in the sample mirrors the actual enrollment patterns of fourth-year science and engineering students in Ethiopian public universities. In these disciplines, enrollment is characterized by pronounced male dominance; for example, among approximately 1,377 fourth-year students, only about 334 are female—a disparity largely attributable to prevailing sociocultural factors. Because the study employed simple random sampling, the sample naturally reproduced this demographic imbalance. Simple random sampling was chosen to ensure that every student in the selected departments had an equal chance of selection; however, this method does not control for inherent subgroup imbalances when the target population is skewed. The documented imbalance—367 males versus 133 females—accurately reflects the real-world context and is not a flaw in the sampling procedure. For future research requiring more balanced subgroup representation, alternative sampling methods, such as stratified random sampling, may be considered to ensure proportional representation of key demographics (e.g., gender).

Prior to data processing, the frequency distribution and the minimum and maximum scores for each item were used to verify that the database had been entered accurately. Additionally, each measure’s underlying assumptions for CFA were examined, and they were confirmed to be valid for the analysis.

### 2.5 Data collection procedures

In November 2022, data collection commenced immediately following the enrolment of fourth-year students for the first semester. Prior to distribution, the research instrument underwent a rigorous, two-way translation process to ensure both semantic equivalence and cultural appropriateness. Initially, well-qualified translators from the English and Psychology Departments of Wolkite University translated the instrument from English into Amharic. Subsequently, English-language professionals back-translated the Amharic version into English. This bidirectional procedure helped confirm that the key concepts of the Propensity to Cheat Scale (PCS) were accurately reflected in the translated version—a process consistent with established translation guidelines ( Brislin, 1970; Beaton et al., 2000).

To further assure the construct validity of the translation, four subject matter experts—drawing on their expertise in Measurement and Evaluation, Educational Psychology, and Special Needs—reviewed the translated instrument. In addition, three language professionals attended to linguistic clarity and grammatical accuracy during the translation. The back-translation reinforced that the final Amharic version faithfully preserved the original content.

A pilot study with 100 students was subsequently conducted to evaluate both the validity and reliability of the translated instrument. Specifically, the pilot testing assessed face and content validity—ensuring that the items were clear, culturally relevant, and effectively conveyed the intended meaning—and established initial internal consistency measures. The positive outcomes from the pilot study provided strong evidence that the translated Amharic version retained the necessary psychometric properties, confirming its overall validity and reliability before being administered to the full sample.

Approval was obtained from each college dean before the commencement of data collection for this study. The college deans informed the department heads and instructors of the individual institutions about the study’s objectives. Subsequently, arrangements were made for the researchers and data collectors to recruit participants in the classroom. Teachers at the universities were tasked with informing and motivating students to participate in the study and scheduling times for the researchers to speak with them during regular class hours. Before delivering the questionnaires, the fourth-year students selected for the study provided their verbal informed consent.

The study began with a thorough briefing for the participants, which included the goal and objective of the research, the focus of the questionnaire, and the potential benefits of the study for society and individuals. The participants were then asked for their consent to participate voluntarily, with the assurance that they could withdraw at any time without consequences and that all information provided would be kept confidential. Following the briefing, the researchers distributed the Amharic revised version questionnaires to students at Hawassa University, Ambo University, and Wolkite University from November to January 2022 during regular class time.

### 2.6 Methods of data analysis

The data processing procedures began by analyzing incomplete questionnaire responses and discarding any that were not complete. The remaining questionnaires were then coded and entered into a computer for further analysis. The data was cleaned to remove any errors that may have occurred during the coding process. Additional checks were conducted to ensure the accuracy of the data entry and measurement scales. The data was then examined for outliers, normality, skewness, and kurtosis to determine if it was at a normal level.

Confirmatory Factor Analysis (CFA) is a type of measurement-related structural equation modeling (SEM) that is essential for validating the relationships between observed items and their corresponding latent factors ( Brown, 2015). CFA provides metrics to evaluate how well a proposed theoretical model fits the collected data, allowing researchers to refine these relationships within the measurement model ( Stevens, 2012). In the context of social and behavioral sciences, CFA is considered a critical tool for validation ( Brown, 2015).

A CFA was conducted for this purpose in order to strengthen the validity of the suggested model of PTC and validate the 4-factor solution obtained from the EFA. Several model fit indices were used to assess the goodness of fit of the CFA model. Brown (2015) divided model fit indices into three categories as a result: “absolute fit, fit adjusting for model parsimony, and comparative or incremental fit” (p. 71). According to Brown (2015), absolute fit indices, which include the chi-square statistic (χ ^2^) and the standardized root mean square residual (SRMR) indices, evaluate model fit at an absolute level. Collier (2020) offers a relative chi-square test, which is the chi-square value divided by the degrees of freedom (χ ^2^/df ), as a better method to utilize because the chi-square statistic is more sensitive to sample size. According to Collier (2020), a relative chi-square value (χ ^2^/df ) that is considered acceptable may range from 3 to 5. Any number below 3 denotes an excellent model fit, while values between 3 and 5 demonstrate an acceptable level. As a result, the first-order CFA model’s outcomes demonstrated that it had a chi-square statistic (χ ^2^ = 3.877, p = .000).

Parsimony correction indices, sometimes also referred to as absolute fit indices, are the second category of model fit indices ( Brown, 2015). The root-mean-square error of approximation (RMSEA) is the most widely used and advised index in this area, according to Brown (2015). The RMSEA is referred to as an error of approximation index since it measures how well a model matches the population ( Brown, 2015). The purpose of this study is not to determine whether the model applies to the population exactly. The RMSEA is hence “sensitive to the number of model parameters” but “relatively insensitive to sample size” ( Brown, 2015, p. 71). Comparative fit indices, which are also referred to as incremental fit indices and assess the fit of a user-specified solution against a nested baseline model, are the third category of model fit indices, according to Brown’s (2015) taxonomy. The CFI and TLI are the most popular and suggested indices in this category, according to Brown (2015).

In this study, CFA was employed to verify both the measurement and structural models. Specifically, a confirmatory factor analysis was conducted to assess the applicability of the scale and to evaluate the convergent and discriminant validity of students’ propensity for academic fraud. The research utilized a sample of public university students in Ethiopia, marking the first investigation of its kind in this context. By examining the effectiveness of the Propensity for Cheating Scale (PCS) in measuring tendencies toward academic dishonesty among Ethiopian higher education students, this study aims to contribute to the existing body of knowledge and address a notable research gap in this area.

The analysis of the data was conducted using the Statistical Package for the Social Sciences (SPSS) and STATA 14. Initially, data were entered into SPSS version 25, where missing data were validated before being transferred to STATA version 14 for confirmatory factor analysis (CFA). Cronbach’s alpha was employed to assess the internal consistency and reliability of the constructs. Furthermore, to evaluate the construct validity of the newly developed Propensity to Cheat Scale (PCS), both convergent and discriminant validity were examined.

The CFA was specifically utilized to determine whether the sample data aligned with the established propensity to cheat (PTC) measurement model. This model’s factorial structure was initially identified through exploratory factor analysis (EFA) with a sample size of n = 550.

To assess the fit of the established PTC measurement model to the data, CFA was employed along with various metrics: composite reliability (CR), average variance extracted (AVE), maximum shared squared variance (MSV), and average shared squared variance (ASV). These metrics also served to address specific research questions, such as the extent to which the PCS demonstrates convergent validity. Additionally, the discriminant validity of the newly constructed PTC instrument was evaluated in relation to other validated factors, such as academic self-concept, using Pearson correlation analysis.

Data collected from participants were analyzed based on IBM SPSS 25 and STATA 14.

**Table:** 

N o	Objectives	Statistics
1	To identify the underlying variables of propensity towards cheating.	Exploratory Factor Analysis (EFA)
2	To validate the factor model developed from EFA.	Confirmatory Factor Analysis (CFA)
3	To assess the internal consistency of the PTC scale.	Reliability Analysis
4	To determine the extent to which the newly developed PTC differs from the measurements of students’ academic self-concept.	Discriminant validity
5	To identify whether PTC is significantly different from students’ academic achievement.	Discriminant validity
6	To determine the extent to which the newly developed PTC shows convergent validity.	Convergent validity

## 3. Results

### 3.1 Confirmatory Factor Analysis (CFA) and overall construct validity

Before conducting Confirmatory Factor Analysis (CFA) on the PTC measure, an item analysis was performed. This included evaluating item means, inter-item correlations, corrected item-total correlations, and the alpha value if any item was eliminated. Inter-item correlations varied across subscales, with ranges as follows: 0.01 to 0.65 for plagiarism, 0.07 to 0.67 for cheating on tests, 0.11 to 0.71 for cheating on assignments, and 0.48 to 0.77 for library theft. Item-total correlation scores were satisfactory, falling between 0.34 and 0.61 for cheating on tests, 0.39 and 0.67 for assignments, 0.37 and 0.79 for plagiarism, and 0.68 and 0.82 for library theft.

Items were selected based on inter-item correlations above 0.30, item-total correlations greater than 0.50, and mean values centered, following guidelines from Field (2009). The determinant of the correlation matrix was above 0.0001, indicating no multicollinearity issues. An inter-correlation matrix showed sufficient coefficients among certain items, and Pearson’s correlation coefficients were calculated between factors.

Exploratory Factor Analysis (EFA) was performed to examine the underlying factor structure of the items. Items with high cross-loadings, low factor loadings, or those incorrectly loaded on the wrong factors were removed. After the EFA, the final scale consisted of 30 items. These items were further validated using CFA to ensure they accurately measured the intended constructs of the PTC measurement instrument.

Following the exploratory factor analysis conducted in Study 1, the CFA established the dataset’s factor structure. The exploration of factor structure—how variables are related and grouped based on inter-variable correlations—in the CFA confirms the factor structure that was extracted in the EFA ( Brown, 2015). To evaluate model parameters and fit indices across the clustering of independent variables, a CFA was also conducted for all datasets to assess the general construct validity ( Chuang, Weng, & Huang, 2015).

**Figure 1.**
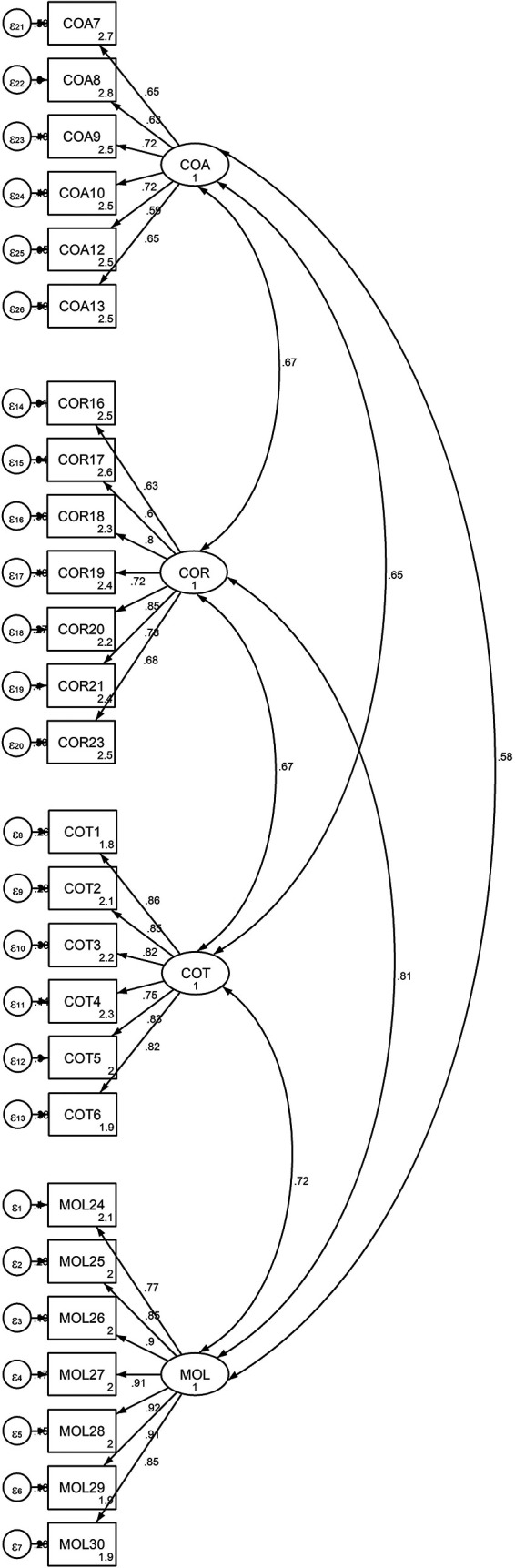
Output of the measurement model for Latent Variable “Four-Factor of PTC” and Observed Indicators.

Both the first-order (measurement model) and the second-order (structural model) were used to validate the model. The findings, as shown in  Figure 1 and  Table 3, strongly support the measurement of PTC using four first-order factors: cheating on tests or examinations, cheating on assignments, cheating on research work, and theft and mutilation of library materials, with a total of 26 items based on the PSC. These items were designed to elicit responses from university students regarding how they perceived their academic cheating.

This figure demonstrates the measurement model for the latent variable Propensity to Cheat (PTC) with four first-order factors: Cheating on Tests (COT), Cheating on Assignments (COA), Cheating on Research Work (COR), and theft and Mutilation of Library Materials (MOL). Each factor is represented by multiple observed indicators (items) that measure the students’ perceived cheating behaviors.

### 3.2 Testing the measurement model

The measurement model was put to the test, and then the structural model was examined using the standards outlined in the literature (see Hair et al., 2016). The measurement models assess the fit of the data to the model and the link between the latent variable and its indicators ( Henseler et al., 2009). The first-order CFA model has been developed to measure the relationships between the four dimensions (cheating on tests, cheating on assignments, cheating on research work, and theft and mutilation of library materials) and the twenty-six associated items that fit the empirical data well. The measurement models for the unobservable (latent) variables were generated and validated using the CFA approach in this study. Because the observed variables of the PTC subscales were determined in advance based on a review of the literature and interviews with experts, a CFA was used to assess the link between cheating on tests, cheating on assignments, cheating on research work, and theft and mutilation of library materials and each of their observed variables in order to develop a measurement model that fit the empirical data well.  Figure 1 below shows the measurement models for the four-factor PTC.  Figure 1 is available in the data repository ( Gizaw, 2024).

Multiple fit indices were employed to assess the appropriateness of the CFA model’s fit to the data, including the χ ^2^/df ratio, Tucker Lewis Index (TLI), comparative fit index (CFI), and root mean square error of approximation (RMSEA). The goodness-of-fit indices of the CFA model for the PCS are shown in  Table 2. Despite the fact that the chi-square statistic has a significant value (χ ^2^/df = 3.877, p =.000), alternative fit indices are encouraging due to the highly sensitive nature of this statistic to large samples ( Bentler & Bonett, 1980; Stevens, 2002). The CFA results demonstrate that the model had fit statistics such as RMSEA = 0.076, RMR = 0.045, TLI = 0.907, and CFI = 0.916. According to the literature, the chi-square value of less than 5 is acceptable, and less than 3 is good ( Marsh & Hocevar, 1985). Hu and Bentler (1999) and Browne and Cudeck (1992) proposed (RMSEA less than.08, RMR less than .05, TLI and CFI greater than .90) as the recommended values for this fit statistic. Based on the indices obtained after CFA, the results indicated that each dimension’s factor model fit each dimension well.

As part of the confirmatory factor analysis (CFA) process, modification indices (MIs) were systematically examined to evaluate potential areas for improving model fit. Although no modifications were made, the review of these indices confirmed that the initial four-factor model was appropriately specified. This practice is consistent with the recommendations of Brown (2015) and Hu and Bentler (1999), and reinforces the transparency and rigor of our analysis. Finally, the coefficient of determination for the entire model is extremely high (CD = 1.000).

**Table 2.** Fit Indices Statistics Output for Measurement and Structural Model Analyses of PCS.

Goodness fit indices	Recommended value	Source(s)	Obtained value of First-order factor model	Obtained value of second-order factor model
Comparative Fit Index (CFI)	>0.90	Bentler, 1990	0.916	0.913
Tucker-Lewis Index (TLI)	>0.90	Tucker and Lewis, 1973 Hair et al., 2010	0.907	0.904
Root mean square error of approximation (RMSEA)	between 0.05 and 0.08		0.076	0.077
Standardized Root-Mean Square Residual (SRMR)	<0.05	Hu and Bentler, 1999	0.045	0.049
Coefficient of determination (CD)			1.000	0.912

### 3.3 Testing the structural model

It is required to move on to the structural model after developing the measurement model. A structural model explains how the many constructs in a model relate to one another. The four measurement models become structural models in the second-order CFA model by linking them collectively or through PTC, as shown below. In order to validate the four-factor structure developed by the exploratory factor analysis (EFA), a second-order CFA was applied. The next phase in structural equation modeling (SEM) analysis is to examine the goodness of fit of the full structural equation model and determine if an acceptable full structural model is obtained after acquiring an acceptable measurement model that fits the empirical data. A set of goodness-of-fit analyses was carried out for this purpose.

**Figure 2.**
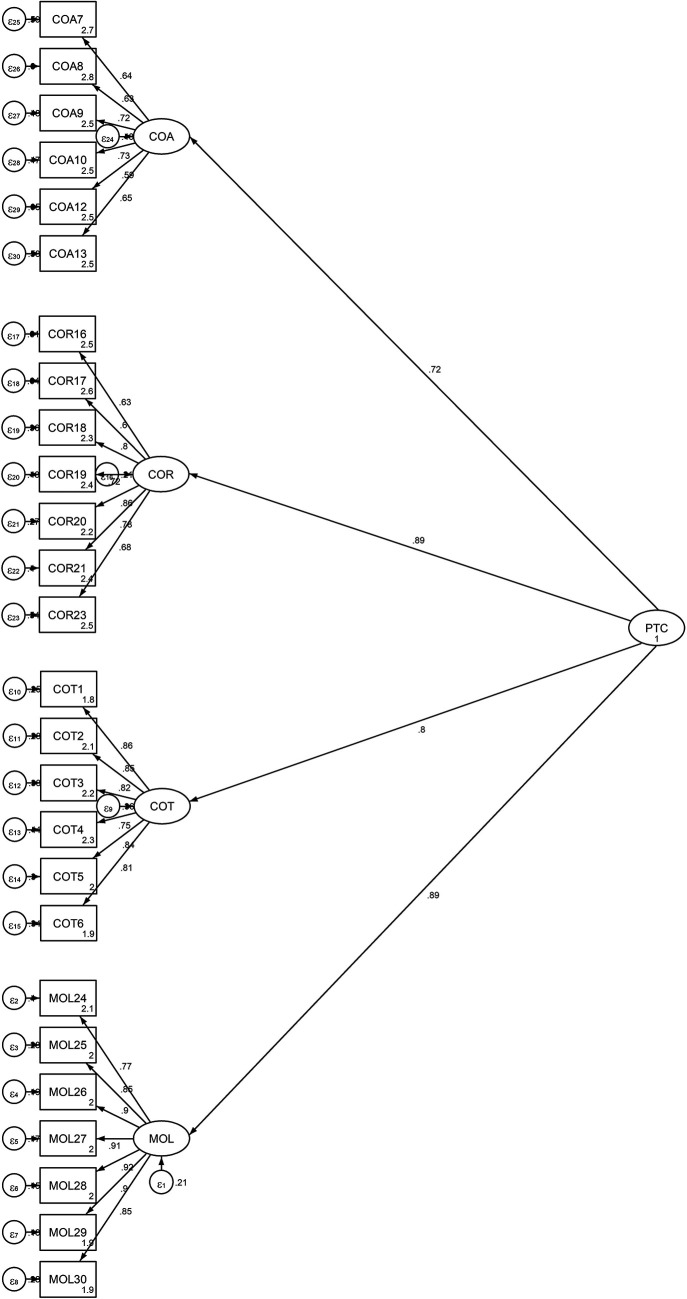
Output of the Structural Model for the Students’ Propensity to Cheat.

Figure 2 is shows the factor distributions and values obtained from the second-order CFA model.  Figure 2 available in the data repository ( Gizaw, 2024).

This figure illustrates the structural model for the student’s propensity to cheat, validated using a second-order Confirmatory Factor Analysis (CFA). The model includes the four first-order factors (COT, COA, COR, and MOL) and their relationships with the overall latent variable PTC. The standardized factor loadings and the coefficients of determination (R ^2^ values) for each observed indicator are displayed, indicating the strength of the relationships between the latent variables and their respective indicators.

To examine the relationships, a structural equation model created with STATA 14 was utilized. A good fitting model is acceptable if the CMIN/df value is less than 5; the Tucker Lewis Index (TLI) ( Tucker and Lewis, 1973); and the confirmatory fit index (CFI) ( Bentler, 1990) is greater than 0.90 ( Hair et al., 2010). In addition, an adequate fitting model was accepted if the standardized Root-Mean Square Residual (SRMR) estimated by STATA was less than 0.05 and the Root Mean Square Error of Approximation (RMSEA) was between 0.05 and 0.08 ( Hair et al., 2010). The alternative fit indices are encouraging due to the sensitivity of the chi-square statistic to sample size: χ ^2^/df = 3.966, CFI = 0.913, TLI = 0.904, RMSEA = 0.077 and RMR = 0.049. The chi-square statistic has a significant value, χ ^2^ (295) = 1170.069, p <0.00. In this regard, the second-order CFA model was suitable since all of the indices met the threshold values of the requirements and because their values were comparable to those of the first-order CFA model. Finally, the total model’s coefficient of determination (CD = 0.912) is very high.

The study used CFA to explore the underlying latent variable structure of the PCS from the same sample on which the EFA was administered in order to evaluate the goodness of fit of the four-factor structure produced from the EFA.

Table 3 and  Figure 2 demonstrate that each factor loading was significant at p<.05. Additionally, the results of the standardized factor loading coefficients showed that the subscales measuring cheating on exams and tests ranged from .75 to .86, the subscales measuring cheating on assignments and research work from .59 to .72, and the subscale measuring theft and mutilation of library materials from .77 to .92.

**Table 3.** Confirmatory Factor Analysis Results for Propensity to Cheat (N = 500).

Latent variable	coding of items	Observed and unobserved variables	Standardized factor loadings	Standard errors	R ^2^ values
Cheating on tests	COT5	I rely on bribes with an instructor to get test information.	.86 *	.01	.74
	COT6	I copy answers from a classmate’s test paper during an exam while the instructor is not looking.	.85 *	.01	.72
	COT8	I use “signals” to ask my classmates for answers during a test.	.82 *	.02	.67
	COT9	I utilize “signal’ to share answers to classmates during an examination.	.75 *	.02	.56
	COT10	I cheat by writing the answers to questions on a soft or handkerchief and pretending to cough while using the soft.	.83 *	.02	.70
	COT11	I personally exchange test papers with someone during a test.	.82 *	.02	.67
Cheating on assignments	COA22	I would cheat on an assignment if I can get an opportunity	.65 *	.03	.42
	COA23	I am likely to cheat on an assignment in the future.	.63 *	.03	.40
	COA26	I submit the project/assignment/paper in my name after getting it prepared by my friends.	.72 *	.03	.52
	COA27	I am resubmitting an assignment from a previous subject in a new subject.	.72 *	.03	.52
	COA29	I provide false justifications to get an extension of a deadline for submitting an assignment.	.59 *	.03	.35
	COA30	I copy a homework assignment from a different portion of the class.	.65 *	.03	.42
Cheating on research work	COR45	I copy and modify a few phrases or sentences from a published work for inclusion in a written research paper without providing acknowledgment to the author.	.63 *	.03	.39
	COR46	I create or falsifying research data, using a secondary source as a primary source.	.60 *	.03	.36
	COR47	I fabricate or falsifying a bibliography.	.80 *	.02	.64
	COR48	I am working on a research paper for another student.	.72 *	.02	.52
	COR49	I pay for a research paper to be written for me.	.85 *	.02	.73
	COR50	I submit a research paper prepared by someone else as my own work, in part or in whole.	.78 *	.02	.60
	COR52	I present a study paper that I got from a "Web site, or online sources," as my own work.	.68 *	.03	.47
Theft and Mutilation of library Material	MOL55	I take out library books so that my classmates do not get required content.	.77 *	.02	.60
	MOL56	I take material from the library without first checking them out.	.85 *	.01	.72
	MOL57	I cut pages out of journals or books in the university library.	.90 *	.01	.81
	MOL58	I eliminate a reference from the library shelf to prevent other students from gaining access to the information.	.91 *	.01	.83
	MOL59	I hide library material in my pocket, handbag, and exercise book.	.92 *	.01	.85
	MOL60	Confusing/diverting the attention of people at the circulation desk.	.91 *	.01	.82
	MOL61	Smuggling it out of the library with the help of library workers.	.85 *	.01	.72
PTC	COT	Cheating on tests	.89 *	.02	.64
	COA	Cheating on assignments	.72 *	.03	.52
	COR	Cheating on research work	.89 *	.02	.79
	MOL	Theft and mutilation of library material	.89 *	.02	.79

* p < 0.05.

Furthermore, the squared multiple correlation coefficients also provide the coefficient of determination (R2), which illustrates the extent to which a factor may account for the variance in an item. In this regard, the COT5 item has the highest R2 (.74), meaning that the latent variable test-cheating accounts for 74% of the variation in the COT5 item.

The model was able to explain 64 percent, 52 percent, 79 percent, and 79 percent, respectively, of the factors related to cheating on tests, cheating on assignments, cheating on research work, and theft and mutilation of library materials (  Table 3). The results showed that, after exam cheating, the two most important factors were theft of library items and exam cheating. Cheating on exams, assignments, research work, and mutilating library materials collectively account for 91% of the variance in the PTC, as indicated by the squared multiple correlation for the PTC of 0.91 (see Figure 2).

## 4. Validity assessment

Analyzing both convergent validity and discriminant validity allows for the examination of construct validity. The construct validity of a scale is examined after its dimensionality and reliability have been confirmed to be appropriate. The CFA also considered the validity of both convergent and divergent findings.

### 4.1 Convergent validity

The standardized factor loading, extracted average variance (AVE), and composite reliability (CR) tests were used to assess convergent validity. Numerous studies have proposed evaluating convergent validity by looking at the statistical significance of standardized factor loadings (e.g., Dunn et al., 1994). For instance, Hair et al. (2009) claimed that all standardized factor loadings should be at least 0.5 and, ideally, at least 0.7, while Stevens (2002) suggested that the value of a factor loading should be more than 0.4 for interpretation purposes. The factor loadings were all greater than 0.59, and some loadings were greater than 0.70 (see  Table 3). Some studies have utilized the Fornell and Larcker (1981) criterion for evaluating convergent validity in addition to looking at the standardized factor loadings (for instance, Yu et al., 2021; Zahoor et al., 2022). According to Fornell and Larcker (1981), convergent validity is demonstrated when a latent construct explains at least half of the variance in the indicators it is related to. To indicate the average amount of variance that a construct explains in its indicators relative to the sum variance of its indicators, they suggested using the average variance extracted (AVE).

The value of AVE for the PTC subscale equals the sum of the average square of factor loadings across all of its indicators divided by the number of items. In order to determine whether the items logged under each facet or domain were estimating the same concept, composite reliability (CR) values of 0.7 or higher and average variance extracted (AVE) values of 0.5 or greater were employed ( Hair et al., 2019). Convergent validity, according to Hair et al. (2009), is seen when the CR is greater than the AVE and the AVE is greater than 0.5.

According to the table, all of the AVE and the items’ total standardized factor loading were both greater than 0.59, which is a sign of good convergent validity ( Hair, Sarstedt, Ringle, & Gudergan, 2017). For this reason, the value of each PTC subscale’s composite reliability was higher than the value of the average variance extracted (see  Table 4). The cheating on research work and cheating on assignments variables’ AVE values (0.37 and 0.38) are below but around the suggested cutoff limit of 0.50 ( Malhotra, 2019), as shown in  Table 4. The construct has acceptable convergent validity if AVE is less than 0.5 but the composite reliability is greater than 0.6 ( Fornell & Larcker, 1981). Exam cheating and library material theft value components show acceptable AVEs of 0.56 and 0.61, respectively, exceeding the 0.5 cutoff and confirming the convergence validity of their latent construct.

**Table 4.** CR, AVE, MSV, and ASV Values for PTC Subscales.

Propensity to Cheat Subscale	CR	AVE	MSV	ASV
Cheating on tests	0.88	0.56	0.37	0.29
Cheating on assignment	0.78	0.38	0.20	0.27
Cheating on research work	0.80	0.37	0.41	0.31
Theft and Mutilation of library Material	0.92	0.61	0.37	0.33

Convergent validity is also demonstrated by the fact that the Maximum Shared Variance for all three variables is less than the pertinent Average Variance Extracted. Our variables have a high level of internal consistency, as seen by the table’s composite reliability of all factors, which is greater than 0.70 ( Gefen et al., 2000).

### 4.2 Discriminant validity of the propensity to cheat scale

Discriminant validity is conducted to ensure that the PCS measures a construct that is distinct from other constructs. Scales that measure theoretically unrelated constructs should, according to discriminant validity, have a low correlation ( DeVellis, 2003; DeVon et al., 2007). Discriminant validity testing is done primarily to demonstrate how different an item or set of items is from others in this study. In other words, the objective of this study is to demonstrate low correlations between items measuring various constructs or variables. This study examined the association between the PCS and other established measures, such as the academic self-concept scale. By assessing these associations, the researchers purpose to reveal the unique contribution of the PCS in measuring PTC in academic settings.

The perceived propensity of students to cheat and their academic self-concept were examined using a bivariate correlational analysis. Bivariate correlation was also used to assess the link between academic achievement and students’ perceived PTC. For the purposes of data analysis, the continuous composite scores on all scales were regarded as interval levels.

Based on a review of the literature, it was predicted that there would be little link between the PTC and academic self-concept. Table 5 displays the relationships between the PTC subscales (MOL, COT, COR, and COA) and academic self-concept (ASC), with the bolded red items denoting discriminant validity. The correlation between the ASC and the subscales (MOL, COT, COR, and COA, respectively) was found to be lower than the correlation between the items of the same construct, such as the respective PTC subscales for MOL, COT, COR, and COA were.678,.741,.586,.520,.571, and.578. This indicates a strong link between items on the PTC subscale alone, but weak correlations between those same items and academic self-concept. To sum up, the results indicate that there is a low relationship between the PTC and academic self-concept, as expected.

**Table 5.** Bivariate Correlations between Academic Self-Concept, PTC Scores, and their Respective Subscales.

PTC Subscales and Academic Self-concept	MOL	COT	COR	COA	ASC
MOL	1				
COT	.678 **	1			
COR	.741 **	.586 **	1		
COA	.520 **	.571 **	.578 **	1	
ASC	.067	.051	.088 *	.121 **	1

Note. MOL = Mutilation of Library Material, COT = Cheating on Test, COR = Cheating on Research Work, COA = Cheating on Assignment, ASC = Academic Self-concept.

** p < 0.01.

* p < 0.05.

Furthermore, research on cheating has shown that there is only a weak negative correlation ( Cizek, 1999) between cheating and grade point average (GPA). Higher GPA students are less likely to report or cheat, but students with lower grades are more likely to do both. For this reason,  Table 6 displays that the correlations between the students’ cumulative grade point averages (CGPA) and their PTC subscales (MOL, COT, COR, and COA) are lower compared to the correlations between the items of the same construct (PTC subscales). This indicates discriminant validity, which is represented by the red-bolded items, as the relationships between the CGPA and the PTC subscales are not as strong as the relationships within the PTC subscales themselves.  Table 6 indicates that there is a lower negative relationship between the CGPA and the MOL, COT, COR, and COA subscales than there is between the items of the same construct, such as the PTC subscales, which have correlations of .678, .741, .586, .520, .571, and.578 (MOL, COT, COR, and COA, respectively).

**Table 6.** Bivariate Correlations between Cumulative Grade Point Average, PTC Scores, and their Corresponding Subscales.

PTC Subscales and Cumulative Grade Point Average	MOL	COT	COR	COA	CGPA
MOL	1				
COT	.678 **	1			
COR	.741 **	.586 **	1		
COA	.520 **	.571 **	.578 **	1	
CGPA	-.004	-.043	-.106 *	-.055	1

Note. MOL = Mutilation of Library Material, COT = Cheating on Test, COR = Cheating on Research Work, COA = Cheating on Assignment, CGPA = Cumulative Grade Point Average.

** p < 0.01.

* p < 0.05.

In short, the PTC and academic self-concept showed a positive, although weak, relationship, indicating discriminant validity. In addition to this, the PCS’s discriminant validity is supported by the low, negative correlations between students’ academic achievement and PTC.

### Discriminant validity

Discriminant validity was assessed using the Fornell–Larcker criterion ( Fornell & Larcker, 1981), which compares the square root of each construct’s average variance extracted (AVE) with its correlations to other constructs ( Malhotra, 1996). As shown in  Table 6a, every construct’s √AVE exceeds its inter-construct correlations, with one exception: the correlation between cheating on research work (COR) and misuse of library materials (MOL) (r = .741) slightly exceeds COR’s √AVE (.61). While this indicates marginal overlap—understandable given that both behaviors involve information-related academic dishonesty—exploratory and confirmatory factor analyses confirmed distinct item loadings for each construct. Moreover, composite reliability (CR) values for all constructs exceeded the.70 benchmark, and all AVE values met recommended thresholds (see  Table 4). Minimal cross-loadings and acceptable model-fit indices (CFI = .916; RMSEA = .076) further support the PCS’s overall construct validity. Despite the modest COR–MOL overlap, the Propensity to Cheat Scale demonstrates acceptable discriminant validity. Consequently, the validated four-factor CFA model can serve as a reliable tool for measuring higher-education students’ propensity to cheat.

**Table 6a.** Correlation between Propensity to Cheat Subscales and Square Root of Average Variance Extracted (√AVE) (n = 500).

PTC Subscales	MOL	COT	COR	COA
**MOL**	**.78**			
**COT**	.678	**.75**		
**COR**	.741	.586	**.61**	
**COA**	.520	.571	.578	**.62**

Note. The bold diagonal values are the square root of average variance extracted (√AVE) for each subscale. The lower off-diagonal values are the correlation between subscales.

## 5. Discussion

### 5.1 Confirmatory factor analysis and model fit

In this study, the models were assessed to determine if they accurately reflected the data using a number of model fit indices. The measurement model outputs of the first-order CFA model-based fit indices in this analysis were CFI = .916, TLI = 0.907, SRMR = .045, and RMSEA = .076; these values suggest that the model had a good fit to the observed data (χ ^2^/df = 3.88, p = .000). In regard to this, the CFI value was 0.92 indicated a satisfactory fit, whereas CFI values are 0.90 and higher as considered acceptable ( Kline, 2023; Thompson, 2004). RMSEA = .077, CFI = .913, RMR = .049, and TLI = .904 are the results of the second-order CFA model run on the scale structure consisting of four factors and 26 items (χ ^2^/df = 3.966, p = .000). The PCS has a valid and reliable factor structure as a result of the structural model’s adequate fit to the sample data. Thus, both the structural and measurement models demonstrated good fit to the empirical data, indicating that the PCS has a valid and reliable factor structure.

### 5.2 Construct validity assessment of propensity to cheat scale

Discriminant validity and convergent validity were used to investigate construct validity. Convergent validity is usually examined first, followed by discriminant validity. According to Collier (2020), this type of construct validity describes the extent to which all indicators of a certain construct are measuring the same thing (construct) that they are supposed to measure. “Evidence that different indicators of theoretically similar or overlapping constructs are strongly interrelated” ( Brown, 2015, p. 2) serves as the basis for this determination.

The term “convergent validity” refers to the closely connected variables inside a single component ( Kaplan & Saccuzzo, 1993). The convergent validity was investigated using factor loading, average variance extracted (AVE), and composite reliability (CR) ( Hair et al., 2017). All factor loadings were higher than 0.59, as shown in the result section. The standard for an item to be included within a factor is having factor loads above .40 ( DeVellis, 2014; Hair, 2009). In addition, if the factor loading was greater than 0.5, it was considered acceptable ( Hidayat et al., 2018). The AVE abbreviation stands for the “average of the squared standardized pattern coefficients for indicators that depend on the same factor but are specified to measure no other factors” ( Kline, 2023, p. 313). All CR was greater than.85, while AVE was over 0.37.

Accordingly, Hair et al. (2010) proposed that convergent validity is achieved when composite reliability is more than average variance extracted (AVE), with both values exceeding .70 and .50, respectively. The AVE values for plagiarism and theft of library resources are 0.56 and 0.61, respectively, which are both higher than the cutoff value of 0.5 and indicate the convergence validity of their latent construct. However, the AVE values for plagiarism in research work and cheating on assignments are slightly below but near the recommended cutoff of 0.50, at 0.37 and 0.38 ( Malhotra, 2019). The construct has adequate convergent validity if the mean variance is less than 0.5 and the composite reliability is greater than 0.6 ( Fornell & Larcker, 1981). In light of this, all subscales in this study satisfied the convergent validity of the constructs ( Fornell & Larcker, 1981). Therefore, these results demonstrate that the scale has achieved convergent validity.

The test of discriminant validity in this study showed that the PCS, academic self-concept, and CGPA are distinct constructs, as evidenced by the low correlations between them. As demonstrated by Gholami et al. (2013), discriminant validity is reached when a construct measures how many indicators are used to indicate only one construct while being genuinely unique from the other constructs. Two latent variables representing various theoretical concepts are statistically different when they have discriminant validity.

In this study, the correlations between the PCS, academic self-concept, and CGPA were tested. A low negative correlation between the CGPA and the PCS would be indicative of discriminant validity. Similarly, it was supposed that evidence of discriminant validity would come from a low correlation between academic self-concept and PCS. The correlation between the PTC subscale, academic self-concept, and CGPA is less than 0.12, as indicated in the results section, indicating that discriminant validity was demonstrated. The findings of this study align with previous research suggesting that Comas-Forgas and Sureda-Negre (2010) suggest that students who struggle academically are more likely to commit plagiarism. As one of the most significant types of PTC, plagiarism, this evidence can be used to demonstrate the validity of the discriminant. Similarly, studies have indicated that students who perform well academically or have higher cumulative grade point averages are less likely to plagiarize ( Guo, 2011).

Discriminant validity was evaluated using the Fornell–Larcker criterion, which was met for all four PTC subscales except for Cheating on Research Work (COR) and Library Material Theft and Mutilation (MOL). Specifically, the inter-construct correlation between COR and MOL (.741) exceeded the square root of COR’s average variance extracted (AVE = .61), indicating a marginal overlap ( Fornell & Larcker, 1981). This convergence likely reflects their shared focus on academic dishonesty in the management of information and institutional resources. Nevertheless, confirmatory factor analysis (CFA) affirmed that all item loadings were strong and distinct (λs ≥ .70) and that composite reliability values exceeded the .70 benchmark; overall model-fit indices also fell within accepted thresholds (e.g., CFI ≥ .916; RMSEA ≤ .076) ( Hair, Black, Babin, & Anderson, 2009; Hu & Bentler, 1999). To enhance conceptual separation, future research should consider mixed-methods designs or refine item wording to more sharply distinguish COR from MOL.

Based on the CFA’s findings, the updated versions of the PCS’s four subscales had high support for the scale’s reliability, discriminant validity, and convergent validity. This study provides evidence that the PCS is a valuable instrument for measuring different aspects of PTC.

The study highlights the validation of the Propensity to Cheat Scale (PCS) through confirmatory factor analysis (CFA), providing higher education institutions with a reliable tool to assess academic dishonesty systematically. The practical benefits of implementing PCS include enabling administrators and instructors to track trends over time, customize classroom interventions to reduce cheating, and identify specific units—such as departments or programs—that exhibit higher risks of academic dishonesty.

These applications align with recent calls within African higher education for adopting data-driven approaches to ethics policies ( Ford, Rothmann, & van Zyl, 2025). Additionally, establishing a standardized measurement like the PCS facilitates benchmarking academic integrity across various institutions, regions, and disciplines. This standardization supports comparative research efforts and aids in developing evidence-based policies to promote academic honesty and integrity.

## 6. Conclusions and practical implications

This scale may be beneficial to assess students’ intent to cheat and to enable further intervention for the widespread practice of cheating in universities. On the basis of examining the fit index values obtained from the first-order CFA, the scale can be considered to have adequate fit values (χ ^2^/df = 3.877, RMSEA = .076, CFI = .916, SRMR = .045) and TLI = 0.907, whereas in the second-order CFA, the scale can be considered to have adequate fit values (χ ^2^/df = 3.966, CFI = 0.913, TLI = 0.904, RMSEA = 0.077, and RMR = 0.049).

In this study, the convergent and discriminant validity of the four PCS factors’ were examined. Convergent validity was assessed based on the factor loading, extracted average variance (AVE), and composite reliability (CR) values of the scale. The AVE values for the factors were as follows: The first factor had a value of 0.61, the second factor had a value of 0.56, the third factor had a value of 0.38, and the fourth factor had a value of 0.37, the CR values were.92, .88, .78, and.80 for the first, second, third, and fourth factors respectively. The scale demonstrated acceptable convergent validity based on these results.

Discriminant validity was assessed by exploring the relationship between the PTC subscale scores, academic self-concept, and grade point average (GPA). In connection to this, the correlation coefficients between the PTC subscales, students’ academic self-concept, and cumulative grade point average (CGPA) were, respectively, (.678, .741, .586, .520, .571 & .578), (.067, .051, .088 & .121), and (.004, .043, .106 & .055). It was observed that there were low correlations between the PTC subscale items, academic self-concept, and GPA. However, there were significant inter-factor correlations within the PTC subscale items.

Based on the findings, the discriminant validity of the PCS was confirmed. Post-analysis, it is clear that the scale’s convergent and discriminant validity were both supported by the data.

The reliability of the scale was examined using Cronbach’s alpha reliability coefficients. Cronbach’s alpha coefficients were calculated for the scale (total score) and subscales of the PTC in order to evaluate the internal consistency. The scale has a high level of internal consistency, as seen by its overall Cronbach’s alpha (.96) ( Field, 2013). The PTC subscales of cheating on tests, cheating on assignments, cheating on research, and theft and mutilation of library materials all had reliability analyses that were, respectively, .93, .85, .90, and.96.

All four factors of PTC have demonstrated satisfactory validity in terms of convergent validity, discriminant validity, and model fit in the final PCS. A high degree of estimation reliability has also been demonstrated by the PCS.

The final PCS now includes 26 and 4 reliable and valid items and factors that may be used to evaluate various PTC aspects.

In summary, this study provides preliminary evidence that the Propensity to Cheat Scale (PCS) exhibits acceptable reliability and validity within Ethiopian public higher education. However, these findings should be interpreted with caution, as the study employed purposive sampling of fourth-year science and engineering students. While purposive sampling effectively targets specific subgroups, it inherently introduces selection bias when the sample does not accurately reflect the broader student population ( Ahmad & Wilkins, 2025). Consequently, this bias may limit the external validity and generalizability of our results. To strengthen future research, it is recommended to replicate these analyses using independent samples and more representative sampling methods—such as stratified random sampling—across diverse student cohorts.

According to the study’s practical implications, academic measurement and evaluation researchers and practitioners can measure the extent of four common PTC types in Ethiopian higher education contexts, especially among university students, using the PCS. This could help professionals accurately assess issues of academic misconduct related to PTC. The PCS was designed and validated because of the study’s contributions to our understanding of academic and professional environments, cheating behavior, and prevention and intervention measures. The study also emphasizes the significance of cultural context in validating psychological scales. The successful validation of the PCS in Ethiopia highlights the necessity for culturally sensitive research practices and the adaptation of existing measures to accommodate diverse educational environments.

The validated PCS provides a strong framework for evaluating and tackling academic dishonesty in Ethiopian public higher education institutions. By using this tool, stakeholders can apply evidence-based strategies to encourage ethical behavior and improve the overall quality of education.

## 7. Limitations and recommendations for future research

Although this study offers academics, practitioners, and students working in the measuring and assessment sector practical advantages, it also has significant limitations that future researchers should address.

Ethiopian public universities serve as the setting for the development and validation of this PCS. Therefore, we suggest researchers at other Ethiopian private universities to examine the validity and reliability of our instrument in relation to their respective academic environments.

Moreover, the purpose of the study to validate an existing instrument was initially to assess a student’s PTC during assessments. Unfortunately, the study did not include this demographic data (family status and socioeconomic status). It is believed that these factors may have an influence on an individual’s PTC. Lastly, in order to measure PTC effectively, researchers and educators need more valid and reliable tools to study and assess PTC.

Finally, a methodological limitation of this study is the use of the same sample for both exploratory factor analysis (EFA) and confirmatory factor analysis (CFA). Although this approach was driven by logistical constraints during data collection, we acknowledge that it may lead to inflated model fit estimates and reduce the generalizability of the findings. Future research should aim to validate the Propensity to Cheat Scale (PCS) using independent samples or split-sample methods to enhance robustness and generalizability.

## Consent of participants

To begin the process of obtaining participants’ willingness to participate in the study, the researchers introduced themselves and provided further explanations about the study. This included details about the participants’ degree of involvement, the objective of the study, the focus of the questionnaire, and the potential benefits of the research for society and individuals.

After receiving verbal consent from the participants, the researchers explained that participation was voluntary. Participants were informed that they could choose to leave the study at any time without facing any penalties or losing any advantages. Additionally, participants were assured that all information provided would be kept confidential. To sum up, before giving students a self-reported questionnaire, researchers got permission from universities. They provided an explanation of the study’s objectives, procedures, and confidentiality safeguards to guarantee anonymity and voluntary participation. This method reduced response bias and addressed ethical issues, leading to more trustworthy data collection.

Following this verbal consent, written informed consent was then obtained from all participants. The written consent form is available in the data repository ( Gizaw, 2024). This process ensured that participants understood and agreed to their participation in the study, while also emphasizing the importance of confidentiality and voluntary participation. Consent of participants available in the data repository ( Gizaw, 2024).

### Ethical issues

The study was conducted in compliance with pertinent standards and regulations and was approved by Hawassa University. After the PhD dissertation proposal was approved and ethical clearance was obtained from Hawassa University’s Office of Vice President for Research and Technology Transfer (VPRTT), the study was carried out. The School of Teacher Education granted approval in this regard on 09/02/2014, with reference number COE-REC/017/24. The data repository contains the ethical approval certificate ( Gizaw, 2024).

## Data availability

### Underlying data

OSF: Propensity to Cheat and Academic Self-concept. 10.17605/OSF.IO/7G9JN ( Gizaw, 2024).

This project comprises the following:

• ** Figure 1.JPEG** output of the measurement model for Latent Variable "Four-Factor of PTC" and Observed Indicators
• ** Figure 2.JPEG** output of the Structural Model for the Students’ Propensity to Cheat.
• SPSS data.xlsx

### Extended data

OSF: Data and materials for propensity to cheat scale.

This project comprises the following:

• Questionnaire.docx
• Informed Consent.docx
• Participation Consent Form
• Permission Certificate
• Ethical Approval Certificate
• Acknowledgement Certifiicate

Data are accessible under the terms of the Creative Commons Attribution 4.0 International license (CC-BY 4.0)
