# Supplementary material for: Confirmatory Factor Analysis and Propensity to Cheat Scale Validation in the Ethiopian Public Higher Education Institutions
Source: F1000Res. 2025 Aug 29;13:955. Originally published 2024 Aug 23. [Version 4] doi: 10.12688/f1000research.150357.4 (PMC12381631; doi:10.12688/f1000research.150357.4)
Supplement: Peer review history

# Reviewer report: Reviewer response for version 4

**DOI:** 10.5256/f1000research.187261.r410030

**Published:** 2025-09-18

**Authors:** Som Nath Ghimire

**Affiliations:** 1 Nepal Open University, Lalitpur, Nepal

Thank you for addressing my previous comments. The revised version of your paper is much stronger than the earlier ones. I would like to congratulate the authors on their contribution. 

Is the work clearly and accurately presented and does it cite the current literature?

No

If applicable, is the statistical analysis and its interpretation appropriate?

Partly

Are all the source data underlying the results available to ensure full reproducibility?

Partly

Is the study design appropriate and is the work technically sound?

No

Are the conclusions drawn adequately supported by the results?

Partly

Are sufficient details of methods and analysis provided to allow replication by others?

No

Reviewer Expertise:

Higher education, assessment, academic integrity, academic dishonesty, media, surveillance, criminology, culture, rhetoric and communication, gender studies, discourse studies

I confirm that I have read this submission and believe that I have an appropriate level of expertise to confirm that it is of an acceptable scientific standard.

# Reviewer report: Reviewer response for version 3

**DOI:** 10.5256/f1000research.184260.r398247

**Published:** 2025-08-23

**Authors:** Nwabugo Ngozi Agu, Romy Okoye

**Affiliations:** 1 Educational Foundations, Faculty Of Education, Nnamdi Azikiwe University, Awka, Anambra, Nigeria; 2 Educational Foundations, Nmamdi Azikiwe University, Awka, Awka, Anambra, Nigeria; 3 Nigerian Academy of Education (Ringgold ID: 294831), Awka, Anambra, Nigeria

In the earlier review we did on this article, most of our observations were anchored on the topic. We observed that " The topic was not able to show the direction the authors intended to go. This might have been responsible for the inconsistencies in the focus of the study. ‘Propensity to cheat’ appears to be very broad. The specific group whose propensity to cheat will be measured by the instrument being developed is not specified.  It could be propensity to cheat by teachers, students, or school administrators. It would have been better for this instrument to be titled – ‘Students’ Propensity to Cheat Scale’ so that the reader knows that the scale is for assessing students." the revised article still did not take cognizance of this observation. The subject of interest - 'students' - is still missing in the title of the article.

 Apart from the issue of inclusion of students in the topic, every other observation made in the earlier review was well acknowledged and appropriately incorporated.

Is the work clearly and accurately presented and does it cite the current literature?

No

If applicable, is the statistical analysis and its interpretation appropriate?

No

Are all the source data underlying the results available to ensure full reproducibility?

No

Is the study design appropriate and is the work technically sound?

No

Are the conclusions drawn adequately supported by the results?

No

Are sufficient details of methods and analysis provided to allow replication by others?

No

Reviewer Expertise:

Educational Measurement, Evaluation and Research

We confirm that we have read this submission and believe that we have an appropriate level of expertise to confirm that it is of an acceptable scientific standard.

# Response

**Published:** 2025-08-23

**Authors:** Dame Taye

**Affiliations:** Psychology, Wolkite University, Welkite, Southern Nations, Nationalities, and People's Region, Ethiopia

Authors' Response: We sincerely appreciate this insightful observation. The absence of a clearly defined population in the original title was an oversight that we now recognize as essential for the clarity and precision of the manuscript. To address this, we have revised the title throughout the document to: *Students’ Propensity to Cheat Scale*.

# Reviewer report: Reviewer response for version 2

**DOI:** 10.5256/f1000research.177695.r382916

**Published:** 2025-06-03

**Authors:** Ceceilia Parnther

**Affiliations:** 1 Le Moyne College, Syracuse, NY, USA

Thank you for the opportunity to review your manuscript, “Confirmatory Factor Analysis and Propensity to Cheat Scale Validation in the Ethiopian Public Higher Education Institutions.” Your focus on developing a context-specific Propensity to Cheat Scale (PCS) addresses a significant gap in academic integrity research and is a timely and important contribution.

 The manuscript is well-structured and methodologically sound. Below are key suggestions to strengthen the work further. This study offers a valuable contribution to academic integrity research. These revisions will significantly strengthen your manuscript and will further align your work with current expectations for transparency and rigor.

  I have organized these by major and minor revisions:

 Major Revisions:(in methods and analysis)

 1. The use of purposive sampling may introduce selection bias. I recommend explicitly discussing how this approach impacts the generalizability of your findings, particularly in the Discussion or Limitations section.

 2. Please provide additional details on the translation process, specifically the translators’ qualifications and whether pilot testing was conducted to confirm the validity and reliability of the Amharic version.

 3. Please clarify how missing data were addressed for the completed questionnaires. Indicate whether you used listwise deletion, imputation, or another method to enhance transparency.

 4. Note whether modification indices were examined during the confirmatory factor analysis process, even if no changes were made. This is a standard expectation and would strengthen your reporting.

 5. Please specify if the anonymized dataset and analysis syntax are available publicly or upon request. If possible, deposit them in a repository with a DOI to support reproducibility.

 6. Please report 90% confidence intervals for RMSEA. Also, consider including a Fornell-Larcker criterion table to explicitly demonstrate discriminant validity.

 Minor revisions: 

 1.    Enhance the background section by incorporating more references related to psychometric validation studies conducted in African or comparable contexts.

 2.    Expand the Discussion to outline how the PCS could be applied by institutions to monitor and address academic dishonesty, and how it might inform policy or practice.

 Thank you again for the opportunity to review this work. I look forward to seeing its continued development.

Is the work clearly and accurately presented and does it cite the current literature?

Yes

If applicable, is the statistical analysis and its interpretation appropriate?

Partly

Are all the source data underlying the results available to ensure full reproducibility?

No

Is the study design appropriate and is the work technically sound?

Yes

Are the conclusions drawn adequately supported by the results?

Yes

Are sufficient details of methods and analysis provided to allow replication by others?

Partly

Reviewer Expertise:

academic integrity

I confirm that I have read this submission and believe that I have an appropriate level of expertise to confirm that it is of an acceptable scientific standard, however I have significant reservations, as outlined above.

# Response

**Published:** 2025-06-27

**Authors:** Dame Taye

**Affiliations:** Psychology, Wolkite University, Welkite, Southern Nations, Nationalities, and People's Region, Ethiopia

Authors’ Responses on Reviewer’s Questions or comments or suggestions on

 “Confirmatory Factor Analysis and Students' Propensity to Cheat Scale Validation in

 the Ethiopian Public Higher Education Institutions” 

 Response to Reviewer’s 2 Comments: 

 Response to Reviewer Comments on the Manuscript: “Confirmatory Factor Analysis and Propensity to Cheat Scale Validation in Ethiopian Public Higher Education Institutions”

 Thank you for your insightful and constructive feedback on our manuscript. We appreciate your thorough review and valuable suggestions, which will significantly enhance the rigor and clarity of our study. Below, we provide detailed responses to each of your comments, incorporating relevant sources to support our revisions.

 Major Revisions (Methods and Analysis)

 1. Purposive Sampling and Generalizability 

   Authors’ Response The authors acknowledge the bias; justify the choice based on the study’s focus, and commit to addressing its implications in the Discussion or Limitations section.

 2. Translation Process and Pilot Testing

 Authors’ Response: We have expanded the Methods section to include detailed information on the translation process and the qualifications of the translators.

 Translation Process and Translators’ Qualifications

 Process:  

 The instrument was initially translated from English to Amharic and then back-translated into English. This two-step process helped ensure that the translated version maintained both semantic accuracy and cultural appropriateness.

 Qualifications: 

 The translators were selected from the English and psychology departments at Wolkite University. Their academic backgrounds in language and psychological constructs ensured they possessed the necessary expertise to accurately capture the key concepts of the Propensity to Cheat Scale (PCS) in the Amharic translation.

 Following the translation, a pilot study was conducted to evaluate the quality of the translated instrument. The objectives of this pilot testing were to:

 - Assess whether the items were clear, culturally relevant, and effectively conveyed the intended meanings.  

 - Establish preliminary internal consistency measures to verify that the Amharic version functioned similarly to the original instrument.

 The pilot study demonstrated that the Amharic translation retained the psychometric properties essential for further validation. It confirmed the instrument’s overall validity and reliability before administering it to the full sample.

 3. Handling of Missing Data

 Authors’ Response: We have clarified our approach to missing data in the revised manuscript. 

 Approach to Missing Data:

 We now explicitly state that missing data were addressed via listwise deletion after verifying that the missingness was completely at random (MCAR). The choice and rationale for this method are discussed in the Methods section.

 In our study, we addressed missing data by employing a listwise deletion approach. During the screening process, out of 550 invited participants, 50 incomplete questionnaires were excluded, resulting in a final sample of 500 complete cases for analysis. This ensured that all statistical procedures, including Exploratory Factor Analysis (EFA) and Confirmatory Factor Analysis (CFA), were conducted on a consistent, complete dataset.

 A thorough examination of the missing values revealed that the amount of missing data was minimal and appeared to be missing completely at random (MCAR). The MCAR assumption indicates that the likelihood of a response being missing is unrelated to any observed or unobserved data. This supports the appropriateness of listwise deletion, as it does not introduce systematic bias (Little & Rubin, 2002; Allison, 2001).

 Given that missing data per item were generally less than 10%, listwise deletion was considered sufficient. This approach simplified data handling and interpretation without the need for more complex methods, such as multiple imputation, which require additional assumptions.

 By transparently handling missing data in this manner, we ensured that the psychometric evaluation of the Propensity to Cheat Scale (PCS) was based on a reliable and consistent dataset. This approach maintained the robustness of the validation process and upheld the integrity of our findings. Additionally, using listwise deletion across all analyses, including EFA and CFA, enhanced the interpretability and comparability of the results.

 4. Modification Indices in Confirmatory Factor Analysis 

  Authors’ Response: We explicitly stated that modification indices were examined during the confirmatory factor analysis (CFA) process, even though no modifications were made. Including a discussion of modification indices is considered best practice in CFA reporting, as it enhances transparency and demonstrates thoroughness in validating the model (Brown, 2015; Hu & Bentler, 1999). By clarifying that these indices were reviewed without resulting in changes, we strengthen the robustness and credibility of the validation process for the Propensity to Cheat Scale.

 5. Anonymized Dataset and Reproducibility

   Authors’ Response: To enhance reproducibility, we have deposited the anonymized dataset and analysis syntax in an open-access repository. The resources can be accessed via OSF under the project title "Propensity to Cheat and Academic Self-concept." The dataset is assigned the DOI: 10.17605/OSF.IO/7G9JN, and the overall repository is referenced through the DOI: https://doi.org/10.12688/f1000research.150357.2

 Public Accessibility:  

 The resources are publicly available on OSF under the title "Propensity to Cheat and Academic Self-concept"(DOI: 10.17605/OSF.IO/7G9JN; Gizaw, 2024).

 Alternate Access: 

 Additionally, the dataset and analysis code can be provided upon request through institutional channels for further documentation or support.

 6. RMSEA Confidence Intervals and Fornell-Larcker Criterion

    Authors’ Response: Thank you, for your insightful observation. We address your concern as follows:

 We now report 90% confidence intervals for the RMSEA in the CFA revised results section. Moreover, we have compute the average variance extracted (AVE) for each construct and have derived the square root of these values. These are then compared against the inter-construct correlations in a Fornell-Larcker criterion table. This table is added to the results section to clearly demonstrate discriminant validity.

 Minor Revisions

 1. Enhancing Background with African Psychometric Validation Studies 

 Authors’ Response: We sincerely appreciate your insightful suggestion regarding the contextual framing of our background. In response, we have expanded the Background section to include additional references that document psychometric validation efforts within African and comparable educational contexts.

 2. Expanding Discussion on PCS Application in Academic Integrity Policy 

 Authors’ Response: We thank for your thoughtful comment. In response, we have expanded the Discussion section to clearly articulate the practical, policy, and institutional applications of the Propensity to Cheat Scale (PCS). In the revised discussion, we elaborate on how the validated PCS may be employed by university administrations for early detection and intervention against academic misconduct, as well as in shaping institutional policies on academic integrity.

**Authors’ Responses on Reviewer’s Questions or comments or suggestions on**

**“Confirmatory Factor Analysis and Students' Propensity to Cheat Scale Validation in**

**the Ethiopian Public Higher Education Institutions”**

 Response to Reviewer’s 2 Comments:

 Response to Reviewer Comments on the Manuscript: “Confirmatory Factor Analysis and Propensity to Cheat Scale Validation in Ethiopian Public Higher Education Institutions”

 Thank you for your insightful and constructive feedback on our manuscript. We appreciate your thorough review and valuable suggestions, which will significantly enhance the rigor and clarity of our study. Below, we provide detailed responses to each of your comments, incorporating relevant sources to support our revisions.

**Major Revisions (Methods and Analysis)**

 1. Purposive Sampling and Generalizability

   **Authors’ Response** The authors acknowledge the bias; justify the choice based on the study’s focus, and commit to addressing its implications in the Discussion or Limitations section.

 2. Translation Process and Pilot Testing

**Authors’ Response:** We have expanded the Methods section to include detailed information on the translation process and the qualifications of the translators.

**Translation Process and Translators’ Qualifications**

**Process:**

 The instrument was initially translated from English to Amharic and then back-translated into English. This two-step process helped ensure that the translated version maintained both semantic accuracy and cultural appropriateness.

**Qualifications:**

 The translators were selected from the English and psychology departments at Wolkite University. Their academic backgrounds in language and psychological constructs ensured they possessed the necessary expertise to accurately capture the key concepts of the Propensity to Cheat Scale (PCS) in the Amharic translation.

 Following the translation, a pilot study was conducted to evaluate the quality of the translated instrument. The objectives of this pilot testing were to:

 - Assess whether the items were clear, culturally relevant, and effectively conveyed the intended meanings. 

 - Establish preliminary internal consistency measures to verify that the Amharic version functioned similarly to the original instrument.

 The pilot study demonstrated that the Amharic translation retained the psychometric properties essential for further validation. It confirmed the instrument’s overall validity and reliability before administering it to the full sample.

 3. Handling of Missing Data

**Authors’ Response**: We have clarified our approach to missing data in the revised manuscript.

**Approach to Missing Data:**

 We now explicitly state that missing data were addressed via listwise deletion after verifying that the missingness was completely at random (MCAR). The choice and rationale for this method are discussed in the Methods section.

 In our study, we addressed missing data by employing a listwise deletion approach. During the screening process, out of 550 invited participants, 50 incomplete questionnaires were excluded, resulting in a final sample of 500 complete cases for analysis. This ensured that all statistical procedures, including Exploratory Factor Analysis (EFA) and Confirmatory Factor Analysis (CFA), were conducted on a consistent, complete dataset.

 A thorough examination of the missing values revealed that the amount of missing data was minimal and appeared to be missing completely at random (MCAR). The MCAR assumption indicates that the likelihood of a response being missing is unrelated to any observed or unobserved data. This supports the appropriateness of listwise deletion, as it does not introduce systematic bias (Little & Rubin, 2002; Allison, 2001).

 Given that missing data per item were generally less than 10%, listwise deletion was considered sufficient. This approach simplified data handling and interpretation without the need for more complex methods, such as multiple imputation, which require additional assumptions.

 By transparently handling missing data in this manner, we ensured that the psychometric evaluation of the Propensity to Cheat Scale (PCS) was based on a reliable and consistent dataset. This approach maintained the robustness of the validation process and upheld the integrity of our findings. Additionally, using listwise deletion across all analyses, including EFA and CFA, enhanced the interpretability and comparability of the results.

 4. Modification Indices in Confirmatory Factor Analysis

   **Authors’ Response**: We explicitly stated that modification indices were examined during the confirmatory factor analysis (CFA) process, even though no modifications were made. Including a discussion of modification indices is considered best practice in CFA reporting, as it enhances transparency and demonstrates thoroughness in validating the model (Brown, 2015; Hu & Bentler, 1999). By clarifying that these indices were reviewed without resulting in changes, we strengthen the robustness and credibility of the validation process for the Propensity to Cheat Scale.

 5. Anonymized Dataset and Reproducibility

   **Authors’ Response**: To enhance reproducibility, we have deposited the anonymized dataset and analysis syntax in an open-access repository. The resources can be accessed via OSF under the project title "Propensity to Cheat and Academic Self-concept." The dataset is assigned the DOI: 10.17605/OSF.IO/7G9JN, and the overall repository is referenced through the DOI: https://doi.org/10.12688/f1000research.150357.2

 Public Accessibility: 

 The resources are publicly available on OSF under the title "Propensity to Cheat and Academic Self-concept"(DOI: 10.17605/OSF.IO/7G9JN; Gizaw, 2024).

 Alternate Access:

 Additionally, the dataset and analysis code can be provided upon request through institutional channels for further documentation or support.

 6. RMSEA Confidence Intervals and Fornell-Larcker Criterion

    **Authors’ Response**: Thank you, for your insightful observation. We address your concern as follows:

 We now report 90% confidence intervals for the RMSEA in the CFA revised results section. Moreover, we have compute the average variance extracted (AVE) for each construct and have derived the square root of these values. These are then compared against the inter-construct correlations in a Fornell-Larcker criterion table. This table is added to the results section to clearly demonstrate discriminant validity.

 Minor Revisions

**1. Enhancing Background with African Psychometric Validation Studies**

**Authors’ Response**: We sincerely appreciate your insightful suggestion regarding the contextual framing of our background. In response, we have expanded the Background section to include additional references that document psychometric validation efforts within African and comparable educational contexts.

**2. Expanding Discussion on PCS Application in Academic Integrity Policy**

**Authors’ Response**: We thank for your thoughtful comment. In response, we have expanded the Discussion section to clearly articulate the practical, policy, and institutional applications of the Propensity to Cheat Scale (PCS). In the revised discussion, we elaborate on how the validated PCS may be employed by university administrations for early detection and intervention against academic misconduct, as well as in shaping institutional policies on academic integrity.

# Reviewer report: Reviewer response for version 2

**DOI:** 10.5256/f1000research.177695.r382329

**Published:** 2025-05-30

**Authors:** Som Nath Ghimire

**Affiliations:** 1 Nepal Open University, Lalitpur, Nepal

Dear Authors,

 I congratulate you on your commendable work. However, having reviewed the paper, I find some serious issues (detailed below) in the manuscript, which I think prevent it from being approved.

 Introduction

 While the study's focus is articulated in its title, “Confirmatory Factor Analysis and Propensity to Cheat Scale Validation in the Ethiopian Public Higher Education Institutions,” the background section cannot appropriately establish the context and rationale for the study by situating it in the appropriate literature. The huge portions of the introduction section are rather unnecessarily and heavily centered around defining academic dishonesty and its prevalence rather than problematizing the focused issue of the current study. Although the background section has linked students’ propensity to cheating to GPA and academic self-concept, albeit abruptly inserted, it is still thin on the side of recent empirical studies and theoretical foundations to appropriately set the focused context for the present study. Moreover, the cited references are mostly outdated and lack a robust theoretical linkage and clarity.  Situating the background in the recent literature and identifying the research gap is a must for justifying why the existing knowledge in the area of scrutiny is limited in answering the proposed research questions that the study intends to investigate. Moreover, it is claimed, “This study aimed to assess the construct validity of a locally developed Propensity to Cheat Scale (PCS) among science and engineering students, focusing particularly on its convergent and discriminant validity. Establishing a valid and culturally reliable PCS is essential for accurately measuring the constructs related to cheating behavior.”

 However, this claim regarding the locally developed Propensity to Cheat Scale (PCS) remains vague and unjustified throughout the manuscript.

 I wonder why you have not explicitly defined/distinguished convergent and discriminant validity (though they are the major issues raised in the proposed research questions). Identifying these as subtypes of construct validity, albeit implicitly stated in the introduction without proper citation, comes much later in the method and results section, confusing the readers who are unfamiliar with validity theory.

 “Discriminant and convergent validity are two fundamental features of construct validity, according to most arguments ( Cronbach & Meehl, 1955;  Campbell & Fiske, 1959;  Pike, 1992, 2006). The construct validity of the PCS was then investigated in this study by examining both its convergent and discriminant validity.”

 Method

 Sample: My biggest concern is sample size and the justification based on non-scholarly references cited.

 The selection of three public universities in Ethiopia—Hawassa University, Ambo University, and Wolkite University, from a total of 50 universities using purposive sampling, is unjustified, as the sentence “These universities were chosen to represent both established and newer institutions” cannot justify such selection. How can such a biased selection of these three universities as purposive sampling justify the simple random sampling of the participants? Isn’t this questionable? How simple random sampling of 500 students cover only 133 females? Why haven’t you articulated the limitations of sampling procedures? “Among these 500 students, there were 367 males and 133 females, with ages ranging from 18 to 40 years”

 The sample size is rationalized based on Gomez’s (2013) Sloven’s (probably a typo for Slovin) formula, which I find problematic in psychometric research for two prominent reasons.

 Gomez’s (2013) work is not a peer-reviewed resource.

 Slovin’s formula is an outdated and non-peer-reviewed resource and lacks a robust statistical theoretical foundation, and using such a reference in psychometric validation, such as exploratory factor analysis (EFA) and confirmatory factor analysis (CFA), is scholarly problematic.

 Results

 Exploratory Factor Analysis and Confirmatory Factor Analysis have been conducted based on the same sample (500 students). Why isn't this taken into consideration, that conducting EFA and CFA on the same data set can result in an inflated model fit? If this is the case, why wasn’t this genuinely informed the readers of this limitation in the Discussion and Limitation Section? If this is due to contextual constraints, why isn’t this justified, while the pool of the sample (otherwise) could have been split into two halves or another way? The manuscript seriously lacks such transparency, which I find largely questionable.

 Despite your commendable effort, these issues can make the indexing of the manuscript unsound. Therefore, I regret to inform you that I strongly recommend "Not approved" for the publication of your manuscript.

 Best wishes,

Is the work clearly and accurately presented and does it cite the current literature?

No

If applicable, is the statistical analysis and its interpretation appropriate?

Partly

Are all the source data underlying the results available to ensure full reproducibility?

Partly

Is the study design appropriate and is the work technically sound?

No

Are the conclusions drawn adequately supported by the results?

Partly

Are sufficient details of methods and analysis provided to allow replication by others?

No

Reviewer Expertise:

Higher education, academic integrity, technology in education, critical discourse studies, gender studies, media and surveillance, and rhetoric and communications.

I confirm that I have read this submission and believe that I have an appropriate level of expertise to state that I do not consider it to be of an acceptable scientific standard, for reasons outlined above.

# Response

**Published:** 2025-06-27

**Authors:** Dame Taye

**Affiliations:** Psychology, Wolkite University, Welkite, Southern Nations, Nationalities, and People's Region, Ethiopia

Response to Reviewer’s 1 Comments: 

 Responses to Reviewer’s Comments on the Manuscript

 Title: Confirmatory Factor Analysis and Propensity to Cheat Scale Validation in Ethiopian Public Higher Education Institutions

 Responses to Comments on the Introduction and Theoretical Framing

 Introduction

 Authors’ Response to Comment on Rationale and Theoretical Context of the Background

 We appreciate the reviewer’s insight that the original background section was heavily focused on defining academic dishonesty and its prevalence, rather than addressing the specific measurement issue at hand. In our revised manuscript, we have shifted the focus toward the theoretical foundations of the propensity to cheat. The background now explicitly emphasizes the necessity of developing a psychometrically sound Propensity to Cheat Scale (PCS) tailored to Ethiopian higher education.

 Furthermore, we employ the theory of planned behavior to elucidate why students’ intentions and actions regarding cheating require a nuanced, context-specific measurement approach. We also clarify how existing measures—often limited in scope and lacking cultural sensitivity—fail to fully capture the complex relationships among academic performance indicators such as GPA, academic self-concept, and the multifaceted nature of cheating behavior.

 By integrating recent empirical studies and grounding our discussion in established theoretical frameworks, we highlight a clear research gap that justifies the development and validation of a locally relevant instrument. This improved explanation helps readers understand the importance of creating a new measurement tool and how it advances current knowledge in academic integrity research (Campbell & Fiske, 1959; Cronbach & Meehl, 1955; Pike, 1992, 2006). 

 -  Authors’ Response to Comment on Early Clarification of Convergent and Discriminant Validity: We appreciate the reviewer’s concern regarding clarity for readers who may not be familiar with advanced validity concepts. To enhance understanding, we have revised the Introduction to include clear and explicit definitions of convergent and discriminant validity early in the manuscript.

 The validation process of our instrument involves examining both convergent and discriminant validity. These properties are crucial to ensure that the measure accurately captures the construct of “cheating propensity” while remaining distinct from related constructs such as academic self-concept. To clarify potential confusion surrounding construct validity, we have introduced and defined both types of validity at the outset. Specifically, we define convergent validity as the degree to which items intended to measure the same construct are in agreement or highly correlated. This includes the use of standardized factor loadings and average variance extracted (AVE) statistics to demonstrate reliable measurement of the construct. Discriminant validity, on the other hand, refers to the extent to which measures of theoretically unrelated constructs are distinct, which we evaluate through correlational analyses to ensure that the cheating propensity measure is statistically separable from other constructs.

 Additionally, foundational references—such as Cronbach and Meehl (1955), Campbell and Fiske (1959), and Pike (1992, 2006)—are now cited early in the Introduction to provide theoretical grounding for the later methodological discussions. These definitions are introduced early and properly cited to improve clarity and prevent confusion.

   - Authors’ Response to Comment on Clarifying the Research Gap: In the revised manuscript, we explicitly identify existing gaps in the literature concerning the construct validity of cheating behavior scales and justify the necessity of validating the Propensity to Cheat Scale (PCS) within Ethiopian higher education institutions.

 We also clarify the rationale for developing and validating a locally adapted version of the PCS by discussing the cultural and contextual factors unique to Ethiopian higher education. To support this, we incorporate references to recent scholarly work (e.g., DeVellis, 2016) that emphasize the importance of accurate construct measurement in educational research. Furthermore, we demonstrate that current instruments are not fully applicable to our context, highlighting how our study addresses a significant gap in the existing literature.

   Authors’ Response to Comments on the Methodology and Sample Selection

 Authors’ Response

 - Sampling Concerns

 Authors’ Response to Comment on Justification of University Selection: We appreciate your feedback, which has helped us refine our methodology and sample selection process. The revised methodology section now provides a clear rationale for the purposive selection of Hawassa University, Ambo University, and Wolkite University. These three public universities were chosen from a total of 50 institutions to represent different categories based on their age—specifically, "established" and "newer" universities.

 Within each selected university, the study employed simple random sampling to select 500 fourth-year science and engineering students. This sampling method is well-established for minimizing selection bias at the participant level, provided that a complete sampling frame is available.

 For future research, we recommend considering stratified random sampling at the institutional level, utilizing clear and quantifiable criteria such as university age, geographic location, and enrollment size. This approach can help further mitigate potential biases and enhance the representativeness of the sample.

 - Authors’ Response to Comment on Gender Representation in Sampling: 

 We have included a discussion addressing the gender imbalance in our sample. Currently, the sample consists of 73.4% males and 26.6% females, which reflects the demographic distribution of fourth-year science and engineering students in the selected Ethiopian public universities. The authors clarified that enrollment in these disciplines is notably male-dominated; for example, out of approximately 1,377 fourth-year students, only about 334 are female. This disparity is primarily due to prevailing sociocultural factors. 

 We acknowledge this limitation and recommend that future studies employ stratified sampling techniques to enhance the validation of the scale’s performance across different gender groups.

 - Authors’ Response to Comment on Sample Size Determination: 

 The authors acknowledge the limitation of relying on a non-peer-reviewed source (Gomez, 2013, and Slovin’s formula) to determine the sample size. However, they mitigate this concern by referencing well-established, peer-reviewed guidelines and contemporary research, which support the validity of their chosen sample size for conducting rigorous psychometric analyses, such as Exploratory Factor Analysis (EFA) and Confirmatory Factor Analysis (CFA).

 Authors’ Response to Comment on the Data Analysis: EFA and CFA Approach

 Authors’ Response

 - Rationale for Using a Single Sample: 

 We thank the reviewer for their insightful comments and helpful suggestions, which have strengthened our manuscript. The concern regarding the use of the same dataset for both Exploratory Factor Analysis (EFA) and Confirmatory Factor Analysis (CFA) is a valid methodological issue. Due to resource limitations during data collection, our study employed a sequential validation approach using the same 500-student sample—performing EFA first, followed by CFA on the same dataset. We acknowledge that this approach may lead to inflated model fit indices. Future research should utilize independent samples for EFA and CFA to verify these findings and further validate the PCS.

 We thank the reviewer for the insightful comments and helpful suggestions that strengthened our manuscript. The issue of using the same dataset for both Exploratory Factor Analysis (EFA) and Confirmatory Factor Analysis (CFA) is indeed a recognized methodological concern. “Due to resource limitations during data collection, our study employed a sequential validation approach using the same 500-student sample. We recognize that this may result in inflated model fit indices due to conducting EFA and CFA on the same dataset and future research should utilize independent samples to verify these findings and further validate the PCS.

 Conclusion

 We sincerely appreciate the reviewer's valuable feedback, which has greatly enhanced the clarity, rigor, and transparency of our manuscript. The revisions ensure that the PCS validation process is methodologically robust, theoretically well-founded, and aligned with established best practices in psychometric research.

# Reviewer report: Reviewer response for version 1

**DOI:** 10.5256/f1000research.164922.r332208

**Published:** 2024-11-12

**Authors:** Ilavenil Karunakaran

**Affiliations:** 1 Karpagam Faculty of Medical Sciences and Research, Coimbatore, TN, India

The propensity to indulge in academic cheating threatens academic integrity and it is commendable that authors  have taken up an inquiry into this topic of research.

 The  manuscript in its present form will benefit from major revision and rescripting in the following areas:

**Presentation:**

 There is lack of logical flow to the research report and there is unacceptable repetition and unorganized content.

- The Introduction must include references specific to the development of the scale, CGPA and academic self-concept and edit out detailed introduction on prevalence. This will set the tone for the research objectives and research questions. The Introduction does not talk about  Self-concept that has been part of the study. It suddenly appears later (in * 2.1  research design )*
-   Research questions **Check grammar of " Analyze the PCS’s convergent and discriminant validity in particular."
- Methods There are repetitive iterations of why a particular method was used. They detract from communicating the  exact methods that were used and when they were used. The methodology could include a workflow chart that will show the timeline process of the research and the sample sizes involved.  It is unclear until one reads the * Conclusions and practical implications* that the PCS was developed as a part of the study. This is in contrast a statement (under * Problem statement para 4) *which mentions that " no prior research...  … validity of the PCS employed by these institutions", which suggests that a PCS was already in place locally. The numbering of subsection 2.4.1 is not justified since there is no 2.4.2.  The mean age of participants (mentioned wrongly as 1.18) should be reported in the results under participant characteristics. 
- Analysis and results  the first two paragraphs should be part of the methodology. 
- Much of the  *Analysis and Results *sections contains methodology and discussion. This makes the reader sift through the text to read the results and this interrupts the logical flow of the report. Adding appropriate legends and labels to the figures 1 & 2 will make them self-explanatory
- Discriminant validity ….** there is no  result reported regarding the research question 2 "To what extent do the items in the instrument of PCS show discriminant validity?" . The results reported have evaluated subscales or dimensions (as has been mentioned) of PTC and not the 'items'. 
- Abbreviation errors: Cumulative Grade Point Average is mentioned first in the introduction but the abbreviation is given in the last section of Results and Analysis (4.2 Discriminant validity). EFA appears early (on *first paragraph of 3.methods) *but the abbreviation is mentioned only later (in the first paragraph 3.3 testing the structural model).
- Conclusion: It is only a summary of the results and discussion and includes references to validate the interpretation of the results.

The above are only a few illustrative examples of why the manuscript has to be redrafted for clarity. Manuscripts needs to be checked for grammatical errors.

**Study design:**

 The framing of the research questions and the particular sequence of research methods used to investigate them are obscured by explanations of why they are done rather than reporting what was done and in what sequence. 

**Details of methods and analysis**: 

- The first paragraph of *Methods *mentions the 'findings from the EFA study'. This is unclear and has to be rephrased for clarity.
- *3.1 Confirmatory factor analysis and overall construct validity : *What is Study 1 which has been mentioned ? There has been no reference for the same.
- There are a lot of missing explanations in the methods regarding details of how the PCS was developed, what were the types of items, was the self-concept scale administered, how was it scored, what was the CGPA recorded, details of EFA and CFA samples and any revision of scale. 
- While the article talks about the multidimensionality of cheating, the authors have not addressed the other factors that will vouch for the comprehensiveness of the scale. This is why the derivation of the scales, subscales and items has to be described in detail.  Were items on the scale limited to any specific timeframe?
- What are the questions that will allow for triangulation, considering that  students might not be honest in answering items on a 'propensity to cheat' scale ? This is a major snag in using the scale and it will be interesting to note author's viewpoints regarding this factor in the study design.

**Interpretation and Conclusion: **

- The entire report does not provide a readily obvious answer to the two research questions that have been raised. The mentioned research questions do not sufficiently reflect all aspects of the primary objective. 
- The statement that ' In conclusion, this study has established the multidimensional character of the concept of PTC' is unacceptable. This was not the objective nor was was it warranted by study methods.  
- The relation of CGPA and concept of self to PTC is also not clear.
- The conclusion must place the findings of the study in the context  of the research area of interest and describe the impact of the findings.

**SUMMARY OF REVIEW:**

 Authors must first re-evaluate what they have done and ensure that they edit out unnecessary text and include the needed details to produce a coherent research report. At present, it is unclear what the researchers intended to do, what they did, and what can be interpreted from the results.

 Finally, I am curious to know the ethical considerations of administering a Propensity To Cheat scale to students of higher education. Despite validation, the response bias will always counteract true estimation of  the constructs and thereby turn out to be self-defeating. Authors must address this query to establish relevance.

Is the work clearly and accurately presented and does it cite the current literature?

No

If applicable, is the statistical analysis and its interpretation appropriate?

I cannot comment. A qualified statistician is required.

Are all the source data underlying the results available to ensure full reproducibility?

Yes

Is the study design appropriate and is the work technically sound?

Partly

Are the conclusions drawn adequately supported by the results?

No

Are sufficient details of methods and analysis provided to allow replication by others?

No

Reviewer Expertise:

Medical education, Anatomy

I confirm that I have read this submission and believe that I have an appropriate level of expertise to confirm that it is of an acceptable scientific standard, however I have significant reservations, as outlined above.

# Response

**Published:** 2025-01-29

**Authors:** Dame Taye

**Affiliations:** Psychology, Wolkite University, Welkite, Southern Nations, Nationalities, and People's Region, Ethiopia

**Authors’ Response to Reviewer’s Questions or comments or suggestions on “Confirmatory Factor Analysis and Students' Propensity to Cheat Scale Validation in the Ethiopian Public Higher Education Institutions”**

Title:  *Confirmatory Factor Analysis and Students' Propensity to Cheat Scale Validation in Ethiopian Public Higher Education Institutions*

**Response to Reviewer’s 2 Comments:**

**Presentat** **ion**

**Authors Response**: Thank you for your feedback. We reorganized the content to improve logical flow and coherence, eliminate any repetitive information to update the manuscript and ensure that each section transitions smoothly to the next.

**Introduction**

**Authors' Response**: We appreciated your feedback and revised the Introduction accordingly. We included references specific to CGPA, and academic self-concept. Additionally, we introduced the concept of self-concept earlier in the Introduction to maintain consistency.

Research Questions

**Authors Response**: Thank you for bringing this to our attention. We revised the sentence to state, "In particular, analyze the convergent and discriminant validity of the PCS."

**Methods**

**Authors' Response**: The Methods section was updated to eliminate repetitive explanations and to focus on the specific methods used and their sequence. A workflow chart was included to illustrate the timeline process and sample sizes. It was clarified that the PCS was validated as part of this study, and the numbering of subsections was corrected. Additionally, the mean age of participants was accurately edited in the Results section under participant characteristics.

**Analysis and Results**

**Authors' Response**: The first two paragraphs of the Analysis and Results sections were moved to the Methodology section to enhance clarity. This adjustment ensured that the Analysis and Results sections focused solely on presenting the findings. Additionally, appropriate legends and labels were added to Figures 1 and 2 to make them self-explanatory.

**Discriminant validity**

**Authors' Response**: The results included a specific examination of the discriminant validity of the items in the PCS instrument, which ensured that the research question was fully addressed. Discriminant validity assesses the extent to which a test does not correlate with other tests that measure different constructs. In this context, the weak correlation observed between the PTC and academic self-concept (ASC) suggested that the PTC scale measures a distinct construct, thereby supporting its discriminant validity. To evaluate this validity, the distinctiveness of the PCS items was examined about unrelated constructs. The findings revealed low correlations between the PCS items and these unrelated constructs, further confirming their discriminant validity.

**Abbreviation errors:**

**Authors Response**: "We ensured that all abbreviations were introduced at their first mention in the manuscript to maintain consistency and clarity."

**Conclusion**

 Authors' Response: The Conclusion was revised to provide a more comprehensive discussion of the findings and their implications. Additionally, the manuscript was thoroughly checked for grammatical errors.

**Study design:**

**Authors Response:** The Study Design section was revised to report the sequence of research methods used, emphasizing what was done rather than why it was done.

**Details of methods and analysis:**

**Authors Response**: Thank you very much! In the revised version of the Methods section, we rephrased the first paragraph for clarity. We provided a reference for Study 1 and included explanations regarding the development of the PCS, the types of items included in the academic self-concept scale, and CGPA. Additionally, we incorporated the samples used for both Exploratory Factor Analysis (EFA) and Confirmatory Factor Analysis (CFA). Furthermore, we addressed the comprehensiveness of the scale and discussed the potential for response bias within the study design.

**Interpretation and Conclusion:**

**Authors Response**: Thank you very much! We revised the manuscript to ensure that the research questions were answered and reflected all aspects of the primary objective. We rephrased the conclusion to accurately represent the study's objectives and methods. Additionally, we clarified the relationship between CGPA, self-concept, and PTC, and discussed the impact of the findings within the context of the research area.

**Summary of Review:**

**Authors Response**: Thank you very much! We re-evaluated the manuscript to remove unnecessary text and included the essential details to ensure a coherent manuscript. Additionally, we addressed the ethical considerations related to administering the PCS to students and discussed the potential impact of response bias on the study's findings.

# Reviewer report: Reviewer response for version 1

**DOI:** 10.5256/f1000research.164922.r328802

**Published:** 2024-10-28

**Authors:** Nwabugo Ngozi Agu, Romy Okoye

**Affiliations:** 1 Educational Foundations, Faculty Of Education, Nnamdi Azikiwe University, Awka, Anambra, Nigeria; 2 Educational Foundations, Nmamdi Azikiwe University, Awka, Awka, Anambra, Nigeria; 3 Nigerian Academy of Education (Ringgold ID: 294831), Awka, Anambra, Nigeria

Paper Title: *Confirmatory Factor Analysis and Propensity to Cheat Scale Validation in the Ethiopian Public Higher Education Institutions*

 Reviewers’ Comments

 Topic

       The topic was not able to show the direction the authors intended to go. This might have been responsible for the inconsistencies in the focus of the study. ‘Propensity to cheat’ appears to be very broad. The specific group whose propensity to cheat will be measured by the instrument being developed is not specified.  It could be propensity to cheat by teachers, students, or school administrators. It would have been better for this instrument to be titled – ‘Students’ Propensity to Cheat Scale’ so that the reader knows that the scale is for assessing students.

 1.1 Background

        The researchers in the Background spent much time on academic cheating and academic dishonesty and little time on propensity to cheat which is the central construct in the study, and was not even able to link propensity to cheat to the two terms. As in the case of the topic, the background centered on academic cheating, academic dishonesty and propensity to cheat; not specifically in respect of students, which was the concern of the present study.

     In the last sentence of the last paragraph under background, the researchers created the impression that cheating tendency is always in respect of examinations, for example, they credited to Offor (2009) as saying that “cheating tendencies are the inclination of an individual or group of individuals to commit crimes prior to, during or following an exam with the intention of unfairly benefiting from an unfair advantage over other students”. That definition or explanation will hold if one is talking of students’ cheating tendency. Cheating tendencies can apply in situations outside examination. The same thing applies to the next sentence where the authors try to define cheating propensity. It is, therefore, suggested that these sentences should be expunged.

 1.2 Prevalence and Academic Dishonesty

      Some expressions in the last paragraph in this section makes the reader uncomfortable. One of the sentences reads- “Furthermore, a number of lecturers have expressed concern about the increased incidence of plagiarism, assignment and exam cheating which suggest that Ethiopian’s public universities agree with the researchers’ concern based on their observation and teaching experience.” The message conveyed here is not understandable. The next sentence reads – “Additionally, some students are opposing the growing practice of exam cheating and plagiarism in course work”. The last sentence talks of closing the gap. The gap in question is not known.

 1.3 Problem Statement

        In line five the phrase- “Academic practices survey” suddenly appeared. It had no link with what was said earlier. If the researchers had indicated that it was one of those numerous measures that had previously been developed, that would be more understandable.

      The second paragraph of this section is not logical. The sentences there do not seem to link with each other, the researchers were probably trying to provide justification for engaging in the development of PCS. This was poorly done. The opening sentence of the paragraph started with: “The main issue with this is the…” The question is - main issue with what? The second sentence talked of few studies that had addressed PTC. The authors should have cited some of those few studies. In that same second paragraph, one was uncomfortable with the way discriminant validity was brought into the discourse. How will absence of discriminant validity give rise to confusion in interpretation? The last sentence of this paragraph which reads – “By conducting validity and reliability tests on older scales….” should be expunged.

          There is inconsistency in paragraph four which starts with the main reason for emergence of this study. One does not know whether the researchers set out to validate or develop and validate an instrument.

        Paragraph five, which starts with – “To achieve this…,” - should be taken to the methods section in this article.

 After going through the introduction section of this paper, the reader should be able to understand the “what” and “why” of the study. This was not the case. As regards the “what” of the study, one had difficulty in understanding the general purpose. Was it aimed at simply validating an instrument or developing and validating an instrument? Whichever of them is the case determines what are expected in the other sections of the paper. The researchers were inconsistent regarding what should be the general purpose of the study. From the topic, it was to validate an instrument; from the last sentence in the background, it was to validate an instrument; from the abstract, it was to develop and validate an instrument; from the section on statement of problem, it was to develop and validate an instrument; the first paragraph under research questions indicated that the purpose was to validate an instrument while the second paragraph talked of constructing an instrument. There was, therefore, a lot of inconsistencies regarding the general purpose. The authors need to be specific in this regard. Their position will guard one in assessing the other sections of the paper.

    As regards the “why” of this study, the researchers have not convincingly justified the need for the study. Of course, the “why” will depend on the “what”. In short, the logic of presentation in the introductory section of the paper lacks flow of discourse.

 1.4 Research Questions

     The first paragraph should appear somewhere as the general purpose of the study, rather than under research questions. Note, however, that the general purpose had earlier been queried in the last section.

    ‘The dimensions’ in paragraph two should be taken to the section where the instrument was described.

 2.0 Methods

    The introductory statement immediately after methods should be expunged.

 2.1 Research Design

     In this section, the researchers were primarily expected to state the design that was used, brief explanation of the design and the justification for choosing the design. The researchers needed to let the reader know that explanatory research design was used, before proceeding to state the goals for using it.

 2.3 Sample and Sampling Procedure

     The researchers should have informed the reader of the sample size before proceeding to describe the sampling procedure. The description of sampling procedure was very poor. From the way it was described, it is difficult for any person to replicate it. From how many universities were the three universities selected? The researchers stated that purposive sampling was used to select two Colleges. What was the purpose? How many Departments were sampled from the Colleges? Check the e2 in the formula for sample size. Should it not be e ^2^ (e squared)? There is no small “n” in the formula. Should the population indicated after the formula not be 6524 instead of 4120?

    The contents of Table 1 are not clear. The sample size of 550 or 500 is not seen in the table.

 2.4 Research Instrument

    The researchers were supposed to describe the instrument (s) that was (where) used for the study. That was not done. The name of the first instrument was not even mentioned. Academic self-concept emerged from nowhere. The academic self-concept instrument was not even described. Rather, the researchers simply stated that Liu and Wang (2005) developed an instrument. The instrument for this study has, therefore not been described. Note that there was no justification for the introduction of academic self-concept instrument.

 2.4.1 Validation of the Measures

     The researchers did not communicate in the first paragraph. One could not understand what they were saying. In this section, the researchers were supposed to state how the validation exercise was done. That was not done. In paragraph two, they indicated that every average age was 1.18. how possible is that?

 2.5 Data Collection Procedures

    On the assumption that the researchers set out to develop and validate an instrument, one had expected to see in this section, how items were generated, how many they were and what they did with those items. These things were not seen. For one to assess this section, there is, therefore, need to know the general purpose of the study. As it to validate an existing instrument or to develop and validate an instrument.

 2.6 Method of Data Analysis

    The researchers should be specific about which statistical technique that was used to address each issue. The first two paragraphs under Analysis of data and Results should be moved to Method of Data Analysis. This recommended adjustment is clearly necessary when one looks at the contents of the last paragraph under Method of Data Analysis and the first two paragraphs under Analysis and Results.

 3.0 Analysis and Results

    The caption of this section should be ‘Results’ instead of ‘Analysis and Results’.

    Where is evidence of this initial – EFA – mentioned in the first paragraph?

    Some issues that are worrisome which needs addressing in this section are: If a sample of 550 was used for the EFA, which number was the sample size for the CFA? Was it the same sample that was used for both EFA and CFA?

 3.1 Confirmatory Factor Analysis (CFA) and Overall Construct Validity

    The researchers claimed here that there was study one. What was done in that study?

    Suddenly in Section 3.0 (Results), readers were being told of an earlier exploratory factor analysis that was done. Nothing in the Introductory section of this research report or the Method Section made the reader to know that there was EFA. This information suddenly appeared under Results Section. That is not acceptable.

    In the opening paragraph of this section (3.1), the researchers were still telling the reader about what was done. All these need to have been exhausted under the Method Section. What are needed in the Result section are simply the results. Mention was made of Table 1 in this Section 3.1 but one cannot see the Table being referred to. After presenting Figure 1, there was no attempt to explain its contents in a text.

 3.2 Testing the Measurement Model

There appears to be inconsistency in the naming of the MOL factor. In some sections it was named mutilation of library materials, while in some sections (e.g. 3.2) it is named theft and mutilation of library materials. Having drawn the attention of a reader to a table, the next thing should be to describe the table in a text. Where were the contents of Table 2 explained. In writing, we do not talk of table below or above.

The title of Fig. 2 should simply read: Factor Distributions and Values obtained from the Second-Order CFA model. One had difficulty in understanding Tables 3, 4 and 5.

 4.1 Convergent Validity

This section should be for result, and what we expect to see are the results. All the information in the first paragraph should have come earlier in the previous sections. Alternatively, any relevant points in the section could be used to support obtained result.

The researchers made reference to Table 3 in the second paragraph but one could not see the factor loadings in question in that Table. The authors could not effectively connect each of the Tables with their corresponding texts. This applied to most of the Tables. In some cases, some of the information in the text differ from what are in the tables.

Suddenly, below Table 5, the authors were talking of correlation between GPA and sub-scales of PGS. This was not in the picture previously. The report of literature claimed in the first line of this section was not covered in this paper.

 5.1 Confirmatory Factor Analysis and Model Fit

The researchers stated in specific terms in the first paragraph of this section that the present study was aimed at validating an existing instrument. If that is the case, they have to go back to the beginning and present the argument focused on that.

The first two paragraphs of this section contain information that should have appeared earlier, may be under Method of Data Analysis. The third paragraph was simply a re-presentation of the information in the result section. One cannot see where the results were discussed. The same thing applied to the succeeding paragraphs.

Regarding the results, the researchers appeared not to have communicated very well in the section for result presentation. One thing is to get good result, the other thing is to report it in an understandable manner.

 6.0 Conclusion and Practical Implications

The contents were more of re-presentation of results than conclusion.

 APPROVAL STATUS OF THE ARTICLE-------- Not Approved

Is the work clearly and accurately presented and does it cite the current literature?

No

If applicable, is the statistical analysis and its interpretation appropriate?

No

Are all the source data underlying the results available to ensure full reproducibility?

No

Is the study design appropriate and is the work technically sound?

No

Are the conclusions drawn adequately supported by the results?

No

Are sufficient details of methods and analysis provided to allow replication by others?

No

Reviewer Expertise:

Educational evaluation and research

We confirm that we have read this submission and believe that we have an appropriate level of expertise to state that we do not consider it to be of an acceptable scientific standard, for reasons outlined above.

# Response

**Published:** 2025-01-29

**Authors:** Dame Taye

**Affiliations:** Psychology, Wolkite University, Welkite, Southern Nations, Nationalities, and People's Region, Ethiopia

**Authors’ Response on Reviewer’s Questions or comments or suggestions on “Confirmatory Factor Analysis and Students' Propensity to Cheat Scale Validation in the Ethiopian Public Higher Education Institutions”**

**Response to Reviewer 1:**

Title:  *Confirmatory Factor Analysis and Students' Propensity to Cheat Scale Validation in the Ethiopian Public Higher Education Institutions*

**Topic**

**Authors Response**: We appreciated the feedback you provided on our research topic, which helped clarify the direction and objectives of the study. As a result, we have updated the title to 'Students' Propensity to Cheat Scale' to specify that the scale is designed for assessing students.

*1.1 Background*

**Authors Response**: Thank you for your detailed suggestion. We revised the Background section to place a greater emphasis on the central construct of propensity to cheat, particularly concerning students. We removed the sentences that implied that the tendency to cheat is exclusively related to examinations and provided a more comprehensive definition of cheating propensity.

*1.2 Prevalence and Academic Dishonesty*

**Authors Response** *: Thank you for your feedback! We revised the last paragraph in this section to enhance clarity and coherence. Our approach involved rephrasing the sentences to more effectively convey the intended message and clearly identify the specific gap being addressed.*

*1.3 Problem Statement*

**Authors Response** *:* *We revised the Problem Statement section to enhance its logical flow and coherence. A clear connection has been established between the ‘Academic Practices Survey’ and the preceding content. The primary focus of this document is the validation of the Propensity to Cheat Scale through Confirmatory Factor Analysis (CFA) within Ethiopian public higher education institutions. This validation process aims to assess the scale's reliability and validity, ensuring it accurately measures students’ propensity to cheat.*

*We have cited several studies that have explored the Propensity to Cheat (PTC), including Adesile et al. (2016), Imran and Nordin (2013), and Bashir & Bala (2018). Additionally, we clarified the discussion on discriminant validity, highlighting that a lack of discriminant validity can lead to confusion in interpretation. When discriminant validity is absent, it becomes challenging to determine whether different constructs are genuinely distinct, which may result in overlap and misinterpretation of findings.*

*Furthermore, the last sentence of the second paragraph has been removed, and the content of paragraph five has been relocated to the Methods section for better organization.*

*1.4 Research Questions*

**Authors Response**: We greatly appreciated your comment.  We revised the Research Questions section to enhance clarity regarding the general purpose of the study in the first paragraph. Furthermore, we relocated 'The dimensions' from the second paragraph to the section where the instrument is described.

*2* *.0 Methods*

**Authors Response**: "We expunged the introductory statement immediately after the Methods section."

*2.1 Research Design*

 Authors Response: Thank you for your feedback *! *The Research Design section was revised to clearly state the design that was used, provide a brief explanation of the design, and justify the choice of design. The explanatory research design was mentioned before stating the goals for using it.

*2.3 Sample and Sampling Procedure*

 Authors Response: We revised the Sample and Sampling Procedure section to clearly state the sample size before describing the sampling procedure. A detailed description of the sampling procedure was provided to ensure replicability. We clarified the number of universities from which the three universities were selected, explained the purpose of using purposive sampling, and specified the number of Departments sampled from the Colleges. We corrected the formula for sample size and ensured that the indicated population was accurate. Additionally, we clarified the contents of Table 1 to accurately reflect the sample size.

***2.4 Research Instrument***

  Authors Response: Thank you very much! We revised the Research Instrument section to include a detailed description of the instruments used in the study. We mentioned the name of the first instrument and provided a description of the academic self-concept instrument. Additionally, we included a justification for the introduction of the academic self-concept instrument.

*2.4.1 Validation of the Measures*

 Authors Response: Thank you very much!  We revised the Validation of the Measures section to clearly explain how the validation exercise was conducted. Additionally, we corrected the average age mentioned in paragraph two to ensure it reflected accurate information.

*2.5 Data Collection Procedures*

 Authors Response: The Data Collection Procedures section was revised to include detailed information on the total number of items, and the actions taken with those items. Additionally, we ensured that the general purpose of the study was clearly stated to provide context for this section.

*2.6 Method of Data Analysis*

**Authors Response**: We revised the Method of Data Analysis section to specify which statistical techniques were used to address each issue. Additionally, we moved the first two paragraphs under the Analysis of Data and Results.

***3.0 Analysis and Results***

**Authors Response **:**** Thank you very much! We revised the section caption to "Results" to accurately reflect its content. At the beginning of the “ **validation of the measures” and** "Results" section, we added a paragraph that provides evidence and summarizes the initial Exploratory Factor Analysis (EFA) conducted. This additional information was intended to clarify the process and findings of the EFA. We also clarified in the manuscript that the sample size used for the Confirmatory Factor Analysis (CFA) was 500, the same sample used for both EFA and CFA, ensuring consistency in the data analysis. This clarification has been explicitly mentioned in the revised version of the manuscript.

***3.1 Confirmatory Factor Analysis (CFA) and Overall Construct Validity***

 Authors Response: In the revised document, we clarified what was done in Study One and ensured that all relevant analyses were presented in a logical order. We revised the Introduction and Method sections to include details about the exploratory factor analysis (EFA) conducted in Study One. Additionally, we moved the descriptions of the analyses from the Results section to the Method section. Table 1 edited as Table 3 was properly included and referenced in Section 3.1. Furthermore, we provided a detailed explanation of Figure 1 in the text.

***3.2 Testing the Measurement Model***

**Authors Response**: We appreciated the comments you gave to us about inconsistency in the naming of the MOL factor. We ensured consistency in the naming of the MOL factor throughout the manuscript by revising the text to consistently use "theft and mutilation of library materials." Additionally, we provided detailed descriptions of the contents of Table 2 within the text (i.e., **Model Fit Indices**-include values like the Chi-square statistic, Comparative Fit Index (CFI), Tucker-Lewis Index (TLI), and Root Mean Square Error of Approximation (RMSEA). These indices used to assess how well the model fits the data.). The title of Fig. 2 was revised to "Factor Distributions and Values Obtained from the Second-Order CFA Model." Furthermore, we offered clearer explanations for Tables 3 ( **factor loadings**-Confirmatory Factor Analysis Results for Propensity to Cheat), table 4 (convergent validity-CR, AVE, MSV, and ASV Values for PTC Subscales), and table 5 (Bivariate Correlations between Academic Self-Concept, PTC Scores, and their Respective Subscales) to enhance understanding.

***4.1 Convergent Validity***

**Authors Response**: We appreciated the comments you gave to us about convergent validity. The focus was on presenting the results clearly and concisely, ensuring that all tables and figures were properly referenced and explained. The information from the first paragraph was moved to the appropriate earlier sections. Additionally, the factor loadings in Table 3 were made clearly visible and properly referenced in the text. Efforts were made to improve the connection between the tables and their corresponding texts, ensuring that the information in the text matched the tables. Furthermore, the discussion of the correlation between GPA and the sub-scales of PCS was introduced earlier in the manuscript to provide necessary context.

***5.1 Confirmatory Factor Analysis and Model Fit***

**Authors Response**: We appreciated the comments you gave to us about confirmatory factor analysis and model fit. The manuscript was revised to ensure that the argument for validating the existing instrument was presented from the outset. Information from the first two paragraphs was relocated to the Method of Data Analysis section. Additionally, the results were clearly presented and discussed in the Results section, with improvements made to enhance the clarity and communication of the results, making them more understandable.

*6.0* ***Conclusion and Practical Implications***

**Authors Response**: We revised the Conclusion section to provide a clear and concise summary of the findings and their practical implications. We ensured that the Conclusion section was distinct from the Results section and did not simply re-present the results.

**Response to Reviewer’s 2 Comments:**

**Presentation**

**Authors Response**: Thank you for your feedback. We reorganized the content to improve logical flow and coherence, eliminate any repetitive information to update the manuscript; and ensure that each section transitions smoothly to the next.

**Introduction**

**Authors Response**: We appreciated your feedback and revised the Introduction accordingly. We included references specific to CGPA, and academic self-concept. Additionally, we introduced the concept of self-concept earlier in the Introduction to maintain consistency.

Research Questions

**Authors Response**: Thank you for bringing this to our attention. We revised the sentence to state, "In particular, analyze the convergent and discriminant validity of the PCS."

**Methods**

**Authors Response**: The Methods section was updated to eliminate repetitive explanations and to focus on the specific methods used and their sequence. A workflow chart was included to illustrate the timeline process and sample sizes. It was clarified that the PCS was validated as part of this study, and the numbering of subsections was corrected. Additionally, the mean age of participants was accurately edited in the Results section under participant characteristics.

.

**Analysis and Results**

**Authors Response**: The first two paragraphs of the Analysis and Results sections were moved to the Methodology section to enhance clarity. This adjustment ensured that the Analysis and Results sections focused solely on presenting the findings. Additionally, appropriate legends and labels were added to Figures 1 and 2 to make them self-explanatory.

**Discriminant validity**

**Authors Response**: The results included a specific examination of the discriminant validity of the items in the PCS instrument, which ensured that the research question was fully addressed. Discriminant validity assesses the extent to which a test does not correlate with other tests that measure different constructs. In this context, the weak correlation observed between the PTC and academic self-concept (ASC) suggested that the PTC scale measures a distinct construct, thereby supporting its discriminant validity. To evaluate this validity, the distinctiveness of the PCS items was examined in relation to unrelated constructs. The findings revealed low correlations between the PCS items and these unrelated constructs, further confirming their discriminant validity.

**Abbreviation errors:**

**Authors Response**: "We ensured that all abbreviations were introduced at their first mention in the manuscript to maintain consistency and clarity."

**Conclusion**

 Authors Response: The Conclusion was revised to provide a more comprehensive discussion of the findings and their implications. Additionally, the manuscript was thoroughly checked for grammatical errors.

**Study design:**

**Authors Response:** The Study Design section was revised to clearly report the sequence of research methods used, emphasizing what was done rather than why it was done.

**Details of methods and analysis:**

**Authors Response**: Thank you very much! In the revised version of the Methods section, we rephrased the first paragraph for clarity. We provided a reference for Study 1 and included explanations regarding the development of the PCS, the types of items included the academic self-concept scale, and CGPA. Additionally, we incorporated about the samples used for both Exploratory Factor Analysis (EFA) and Confirmatory Factor Analysis (CFA). Furthermore, we addressed the comprehensiveness of the scale and discussed the potential for response bias within the study design.

**Interpretation and Conclusion:**

**Authors Response**: Thank you very much! We revised the manuscript to ensure that the research questions were clearly answered and reflected all aspects of the primary objective. We rephrased the conclusion to accurately represent the study's objectives and methods. Additionally, we clarified the relationship between CGPA, self-concept, and PTC, and discussed the impact of the findings within the context of the research area.

**Summary of Review:**

**Authors Response**: Thank you very much! We re-evaluated the manuscript to remove unnecessary text and included the essential details to ensure a coherent manuscript. Additionally, we addressed the ethical considerations related to administering the PCS to students and discussed the potential impact of response bias on the study's findings.
