# Supplementary material for: Confirmatory Factor Analysis and Propensity to Cheat Scale Validation in the Ethiopian Public Higher Education Institutions
Source: F1000Res. 2025 Jul 11;13:955. Originally published 2024 Aug 23. [Version 3] doi: 10.12688/f1000research.150357.3 (PMC12381631; doi:10.12688/f1000research.150357.3)
Supplement: Peer review history

# Response

**Published:** 2025-01-29

**Authors:** Dame Taye

**Affiliations:** Psychology, Wolkite University, Welkite, Southern Nations, Nationalities, and People's Region, Ethiopia

**Authors’ Response to Reviewer’s Questions or comments or suggestions on “Confirmatory Factor Analysis and Students' Propensity to Cheat Scale Validation in the Ethiopian Public Higher Education Institutions”**

Title: *Confirmatory Factor Analysis and Students' Propensity to Cheat Scale Validation in Ethiopian Public Higher Education Institutions*

**Response to Reviewer’s 2 Comments:**

**Presentat** **ion**

**Authors Response**: Thank you for your feedback. We reorganized the content to improve logical flow and coherence, eliminate any repetitive information to update the manuscript and ensure that each section transitions smoothly to the next.

**Introduction**

**Authors' Response**: We appreciated your feedback and revised the Introduction accordingly. We included references specific to CGPA, and academic self-concept. Additionally, we introduced the concept of self-concept earlier in the Introduction to maintain consistency.

Research Questions

**Authors Response**: Thank you for bringing this to our attention. We revised the sentence to state, "In particular, analyze the convergent and discriminant validity of the PCS."

**Methods**

**Authors' Response**: The Methods section was updated to eliminate repetitive explanations and to focus on the specific methods used and their sequence. A workflow chart was included to illustrate the timeline process and sample sizes. It was clarified that the PCS was validated as part of this study, and the numbering of subsections was corrected. Additionally, the mean age of participants was accurately edited in the Results section under participant characteristics.

**Analysis and Results**

**Authors' Response**: The first two paragraphs of the Analysis and Results sections were moved to the Methodology section to enhance clarity. This adjustment ensured that the Analysis and Results sections focused solely on presenting the findings. Additionally, appropriate legends and labels were added to Figures 1 and 2 to make them self-explanatory.

**Discriminant validity**

**Authors' Response**: The results included a specific examination of the discriminant validity of the items in the PCS instrument, which ensured that the research question was fully addressed. Discriminant validity assesses the extent to which a test does not correlate with other tests that measure different constructs. In this context, the weak correlation observed between the PTC and academic self-concept (ASC) suggested that the PTC scale measures a distinct construct, thereby supporting its discriminant validity. To evaluate this validity, the distinctiveness of the PCS items was examined about unrelated constructs. The findings revealed low correlations between the PCS items and these unrelated constructs, further confirming their discriminant validity.

**Abbreviation errors:**

**Authors Response**: "We ensured that all abbreviations were introduced at their first mention in the manuscript to maintain consistency and clarity."

**Conclusion**

 Authors' Response: The Conclusion was revised to provide a more comprehensive discussion of the findings and their implications. Additionally, the manuscript was thoroughly checked for grammatical errors.

**Study design:**

**Authors Response:** The Study Design section was revised to report the sequence of research methods used, emphasizing what was done rather than why it was done.

**Details of methods and analysis:**

**Authors Response**: Thank you very much! In the revised version of the Methods section, we rephrased the first paragraph for clarity. We provided a reference for Study 1 and included explanations regarding the development of the PCS, the types of items included in the academic self-concept scale, and CGPA. Additionally, we incorporated the samples used for both Exploratory Factor Analysis (EFA) and Confirmatory Factor Analysis (CFA). Furthermore, we addressed the comprehensiveness of the scale and discussed the potential for response bias within the study design.

**Interpretation and Conclusion:**

**Authors Response**: Thank you very much! We revised the manuscript to ensure that the research questions were answered and reflected all aspects of the primary objective. We rephrased the conclusion to accurately represent the study's objectives and methods. Additionally, we clarified the relationship between CGPA, self-concept, and PTC, and discussed the impact of the findings within the context of the research area.

**Summary of Review:**

**Authors Response**: Thank you very much! We re-evaluated the manuscript to remove unnecessary text and included the essential details to ensure a coherent manuscript. Additionally, we addressed the ethical considerations related to administering the PCS to students and discussed the potential impact of response bias on the study's findings.

# Response

**Published:** 2025-01-29

**Authors:** Dame Taye

**Affiliations:** Psychology, Wolkite University, Welkite, Southern Nations, Nationalities, and People's Region, Ethiopia

**Authors’ Response on Reviewer’s Questions or comments or suggestions on “Confirmatory Factor Analysis and Students' Propensity to Cheat Scale Validation in the Ethiopian Public Higher Education Institutions”**

**Response to Reviewer 1:**

Title: *Confirmatory Factor Analysis and Students' Propensity to Cheat Scale Validation in the Ethiopian Public Higher Education Institutions*

**Topic**

**Authors Response**: We appreciated the feedback you provided on our research topic, which helped clarify the direction and objectives of the study. As a result, we have updated the title to 'Students' Propensity to Cheat Scale' to specify that the scale is designed for assessing students.

*1.1 Background*

**Authors Response**: Thank you for your detailed suggestion. We revised the Background section to place a greater emphasis on the central construct of propensity to cheat, particularly concerning students. We removed the sentences that implied that the tendency to cheat is exclusively related to examinations and provided a more comprehensive definition of cheating propensity.

*1.2 Prevalence and Academic Dishonesty*

**Authors Response** *: Thank you for your feedback! We revised the last paragraph in this section to enhance clarity and coherence. Our approach involved rephrasing the sentences to more effectively convey the intended message and clearly identify the specific gap being addressed.*

*1.3 Problem Statement*

**Authors Response** *:* *We revised the Problem Statement section to enhance its logical flow and coherence. A clear connection has been established between the ‘Academic Practices Survey’ and the preceding content. The primary focus of this document is the validation of the Propensity to Cheat Scale through Confirmatory Factor Analysis (CFA) within Ethiopian public higher education institutions. This validation process aims to assess the scale's reliability and validity, ensuring it accurately measures students’ propensity to cheat.*

*We have cited several studies that have explored the Propensity to Cheat (PTC), including Adesile et al. (2016), Imran and Nordin (2013), and Bashir & Bala (2018). Additionally, we clarified the discussion on discriminant validity, highlighting that a lack of discriminant validity can lead to confusion in interpretation. When discriminant validity is absent, it becomes challenging to determine whether different constructs are genuinely distinct, which may result in overlap and misinterpretation of findings.*

*Furthermore, the last sentence of the second paragraph has been removed, and the content of paragraph five has been relocated to the Methods section for better organization.*

*1.4 Research Questions*

**Authors Response**: We greatly appreciated your comment.  We revised the Research Questions section to enhance clarity regarding the general purpose of the study in the first paragraph. Furthermore, we relocated 'The dimensions' from the second paragraph to the section where the instrument is described.

*2* *.0 Methods*

**Authors Response**: "We expunged the introductory statement immediately after the Methods section."

*2.1 Research Design*

 Authors Response: Thank you for your feedback *! *The Research Design section was revised to clearly state the design that was used, provide a brief explanation of the design, and justify the choice of design. The explanatory research design was mentioned before stating the goals for using it.

*2.3 Sample and Sampling Procedure*

 Authors Response: We revised the Sample and Sampling Procedure section to clearly state the sample size before describing the sampling procedure. A detailed description of the sampling procedure was provided to ensure replicability. We clarified the number of universities from which the three universities were selected, explained the purpose of using purposive sampling, and specified the number of Departments sampled from the Colleges. We corrected the formula for sample size and ensured that the indicated population was accurate. Additionally, we clarified the contents of Table 1 to accurately reflect the sample size.

***2.4 Research Instrument***

  Authors Response: Thank you very much! We revised the Research Instrument section to include a detailed description of the instruments used in the study. We mentioned the name of the first instrument and provided a description of the academic self-concept instrument. Additionally, we included a justification for the introduction of the academic self-concept instrument.

*2.4.1 Validation of the Measures*

 Authors Response: Thank you very much!  We revised the Validation of the Measures section to clearly explain how the validation exercise was conducted. Additionally, we corrected the average age mentioned in paragraph two to ensure it reflected accurate information.

*2.5 Data Collection Procedures*

 Authors Response: The Data Collection Procedures section was revised to include detailed information on the total number of items, and the actions taken with those items. Additionally, we ensured that the general purpose of the study was clearly stated to provide context for this section.

*2.6 Method of Data Analysis*

**Authors Response**: We revised the Method of Data Analysis section to specify which statistical techniques were used to address each issue. Additionally, we moved the first two paragraphs under the Analysis of Data and Results.

***3.0 Analysis and Results***

**Authors Response **:**** Thank you very much! We revised the section caption to "Results" to accurately reflect its content. At the beginning of the “ **validation of the measures” and** "Results" section, we added a paragraph that provides evidence and summarizes the initial Exploratory Factor Analysis (EFA) conducted. This additional information was intended to clarify the process and findings of the EFA. We also clarified in the manuscript that the sample size used for the Confirmatory Factor Analysis (CFA) was 500, the same sample used for both EFA and CFA, ensuring consistency in the data analysis. This clarification has been explicitly mentioned in the revised version of the manuscript.

***3.1 Confirmatory Factor Analysis (CFA) and Overall Construct Validity***

 Authors Response: In the revised document, we clarified what was done in Study One and ensured that all relevant analyses were presented in a logical order. We revised the Introduction and Method sections to include details about the exploratory factor analysis (EFA) conducted in Study One. Additionally, we moved the descriptions of the analyses from the Results section to the Method section. Table 1 edited as Table 3 was properly included and referenced in Section 3.1. Furthermore, we provided a detailed explanation of Figure 1 in the text.

***3.2 Testing the Measurement Model***

**Authors Response**: We appreciated the comments you gave to us about inconsistency in the naming of the MOL factor. We ensured consistency in the naming of the MOL factor throughout the manuscript by revising the text to consistently use "theft and mutilation of library materials." Additionally, we provided detailed descriptions of the contents of Table 2 within the text (i.e., **Model Fit Indices**-include values like the Chi-square statistic, Comparative Fit Index (CFI), Tucker-Lewis Index (TLI), and Root Mean Square Error of Approximation (RMSEA). These indices used to assess how well the model fits the data.). The title of Fig. 2 was revised to "Factor Distributions and Values Obtained from the Second-Order CFA Model." Furthermore, we offered clearer explanations for Tables 3 ( **factor loadings**-Confirmatory Factor Analysis Results for Propensity to Cheat), table 4 (convergent validity-CR, AVE, MSV, and ASV Values for PTC Subscales), and table 5 (Bivariate Correlations between Academic Self-Concept, PTC Scores, and their Respective Subscales) to enhance understanding.

***4.1 Convergent Validity***

**Authors Response**: We appreciated the comments you gave to us about convergent validity. The focus was on presenting the results clearly and concisely, ensuring that all tables and figures were properly referenced and explained. The information from the first paragraph was moved to the appropriate earlier sections. Additionally, the factor loadings in Table 3 were made clearly visible and properly referenced in the text. Efforts were made to improve the connection between the tables and their corresponding texts, ensuring that the information in the text matched the tables. Furthermore, the discussion of the correlation between GPA and the sub-scales of PCS was introduced earlier in the manuscript to provide necessary context.

***5.1 Confirmatory Factor Analysis and Model Fit***

**Authors Response**: We appreciated the comments you gave to us about confirmatory factor analysis and model fit. The manuscript was revised to ensure that the argument for validating the existing instrument was presented from the outset. Information from the first two paragraphs was relocated to the Method of Data Analysis section. Additionally, the results were clearly presented and discussed in the Results section, with improvements made to enhance the clarity and communication of the results, making them more understandable.

*6.0* ***Conclusion and Practical Implications***

**Authors Response**: We revised the Conclusion section to provide a clear and concise summary of the findings and their practical implications. We ensured that the Conclusion section was distinct from the Results section and did not simply re-present the results.

**Response to Reviewer’s 2 Comments:**

**Presentation**

**Authors Response**: Thank you for your feedback. We reorganized the content to improve logical flow and coherence, eliminate any repetitive information to update the manuscript; and ensure that each section transitions smoothly to the next.

**Introduction**

**Authors Response**: We appreciated your feedback and revised the Introduction accordingly. We included references specific to CGPA, and academic self-concept. Additionally, we introduced the concept of self-concept earlier in the Introduction to maintain consistency.

Research Questions

**Authors Response**: Thank you for bringing this to our attention. We revised the sentence to state, "In particular, analyze the convergent and discriminant validity of the PCS."

**Methods**

**Authors Response**: The Methods section was updated to eliminate repetitive explanations and to focus on the specific methods used and their sequence. A workflow chart was included to illustrate the timeline process and sample sizes. It was clarified that the PCS was validated as part of this study, and the numbering of subsections was corrected. Additionally, the mean age of participants was accurately edited in the Results section under participant characteristics.

.

**Analysis and Results**

**Authors Response**: The first two paragraphs of the Analysis and Results sections were moved to the Methodology section to enhance clarity. This adjustment ensured that the Analysis and Results sections focused solely on presenting the findings. Additionally, appropriate legends and labels were added to Figures 1 and 2 to make them self-explanatory.

**Discriminant validity**

**Authors Response**: The results included a specific examination of the discriminant validity of the items in the PCS instrument, which ensured that the research question was fully addressed. Discriminant validity assesses the extent to which a test does not correlate with other tests that measure different constructs. In this context, the weak correlation observed between the PTC and academic self-concept (ASC) suggested that the PTC scale measures a distinct construct, thereby supporting its discriminant validity. To evaluate this validity, the distinctiveness of the PCS items was examined in relation to unrelated constructs. The findings revealed low correlations between the PCS items and these unrelated constructs, further confirming their discriminant validity.

**Abbreviation errors:**

**Authors Response**: "We ensured that all abbreviations were introduced at their first mention in the manuscript to maintain consistency and clarity."

**Conclusion**

 Authors Response: The Conclusion was revised to provide a more comprehensive discussion of the findings and their implications. Additionally, the manuscript was thoroughly checked for grammatical errors.

**Study design:**

**Authors Response:** The Study Design section was revised to clearly report the sequence of research methods used, emphasizing what was done rather than why it was done.

**Details of methods and analysis:**

**Authors Response**: Thank you very much! In the revised version of the Methods section, we rephrased the first paragraph for clarity. We provided a reference for Study 1 and included explanations regarding the development of the PCS, the types of items included the academic self-concept scale, and CGPA. Additionally, we incorporated about the samples used for both Exploratory Factor Analysis (EFA) and Confirmatory Factor Analysis (CFA). Furthermore, we addressed the comprehensiveness of the scale and discussed the potential for response bias within the study design.

**Interpretation and Conclusion:**

**Authors Response**: Thank you very much! We revised the manuscript to ensure that the research questions were clearly answered and reflected all aspects of the primary objective. We rephrased the conclusion to accurately represent the study's objectives and methods. Additionally, we clarified the relationship between CGPA, self-concept, and PTC, and discussed the impact of the findings within the context of the research area.

**Summary of Review:**

**Authors Response**: Thank you very much! We re-evaluated the manuscript to remove unnecessary text and included the essential details to ensure a coherent manuscript. Additionally, we addressed the ethical considerations related to administering the PCS to students and discussed the potential impact of response bias on the study's findings.
